# Photoswitchable Fluorescence of Peptide-Based Hemipiperazines Inside of Living Cells

**DOI:** 10.1021/jacs.5c07013

**Published:** 2025-07-14

**Authors:** Peter Gödtel, Anna Rösch, Susanne Kirchner, Rabia Elbuga-Ilica, Angelika Seliwjorstow, Olaf Fuhr, Ute Schepers, Zbigniew Pianowski

**Affiliations:** 1 Institute of Organic Chemistry, 150232 Karlsruhe Institute of Technology, Karlsruhe 76131, Germany; 2 Institute of Functional Interfaces, 150232 Karlsruhe Institute of Technology, Eggenstein-Leopoldshafen 76344, Germany; 3 Institute of Nanotechnology and Karlsruhe Nano Micro Facility (KNMFi), 150232 Karlsruhe Institute of Technology, Eggenstein-Leopoldshafen 76344, Germany; 4 Institute of Biological and Chemical Systems − FMS, Karlsruhe Institute of Technology, Eggenstein-Leopoldshafen 76344, Germany

## Abstract

Photoswitchable fluorophores that can be toggled with visible light are extremely useful for applications in super-resolution imaging. However, most small-molecule photoswitches suffer from poor aqueous solubility and limited biocompatibility and require UV-light activation. Here, we report a novel class of biocompatible, visible-light-responsive fluorophores based on hemipiperazine (HPI) scaffolds with annulated π-systems—indolo-hemipiperazines (IndHPIs) and pyrrolo-hemipiperazines (PyrHPIs). These compounds display large Stokes shifts (up to 135 nm), reversible photoisomerization with up to 620 nm wavelength of light, and, in particular examples, enhanced fluorescence quantum yields and switching thereof, augmented by internal H-bonding. Selected compounds demonstrated excellent thermal stability of their E-isomers, with half-lives of up to ∼13,000 h at 50 °C, and high fatigue resistance under repeated switching cycles. Notably, certain IndHPIs are efficiently internalized by living cells and exhibit a reversible modulation of fluorescence upon irradiation. Further mechanistic studies revealed that *in vitro* regeneration of the brighter isomer is mediated by glutathione (GSH) likely *via* a nucleophile-assisted isomerization pathway, providing a possible insight into the cellular behavior of these switches. The exceptional photophysical properties of IndHPIs position them as promising candidates for photoswitchable compounds for application in biological sciences and components of next-generation optical materials.

## Graphical abstract

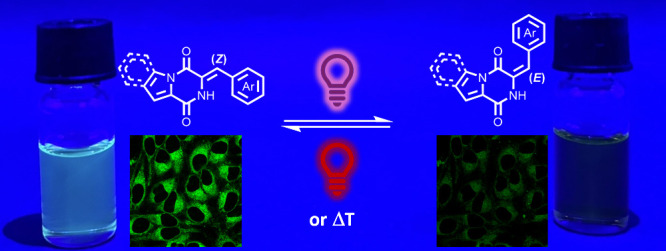

## Introduction

Photoswitchable fluorophores are molecules that reversibly change their emission characteristics upon irradiation with light.^1^ They are applied in high-density optical data storage^2–5^ and in super-resolution microscopy.^6–8^ Switching at room temperature has been observed for fluorescent proteins, such as GFP mutants,^9^ or Dronpa,^10,11^ which can be genetically encoded and expressed in living cells. Fluorescence photoswitching of small molecules was demonstrated for Cy5,^12^ Alexa dyes, and classical molecular photoswitches^13^ such as spiropyrans,^14^ spirooxazines,^15^ thioindigo,^16^ fulgides,^17^ or azobenzenes.^18^ The mechanism usually involves *E*/*Z*-photoisomerization or light-induced ring opening, which, in turn, strongly influences the emissive properties. Among them, fluorescent diarylethenes^6,16^ in particular have proven to be broadly applicable due to their thermal stability and fatigue resistance. However, as most of the photochromic small molecules are hydrophobic, it is often challenging to solubilize them in aqueous media for bioimaging experiments. Elaborate formulation of the fluorophore is not seldom needed.^19^ In addition, the cellular uptake of the fluorophores is often challenging. Finally, many reported systems are triggered by UV light, which does not penetrate biological tissue efficiently.^20^ Developing new inherently polar, visible-light-activated photoswitchable fluorophores that function in aqueous media and are efficiently internalized by living cells is crucial for advancing high-resolution optical bioimaging.^21^

We have recently reported on biocompatible peptide-derived molecular photoswitches, called hemipiperazines (HPIs).^22–25^ Due to the polar nature of a central diketopiperazine ring, which exhibits numerous hydrogen bonding sites, HPIs are operational under aqueous conditions with visible-light frequencies, not degraded in the reducing environment occurring inside of living cells, and thus directly suitable for intracellular application. Furthermore, we demonstrated that the activity of plinabulin—a low-nM inhibitor of microtubule dynamics, which contains the HPI chromophore inside of its structure—can be strongly modulated with light *in vitro* ^22^ and *in vivo* (Figure 1a).^25^

**Figure 1:**
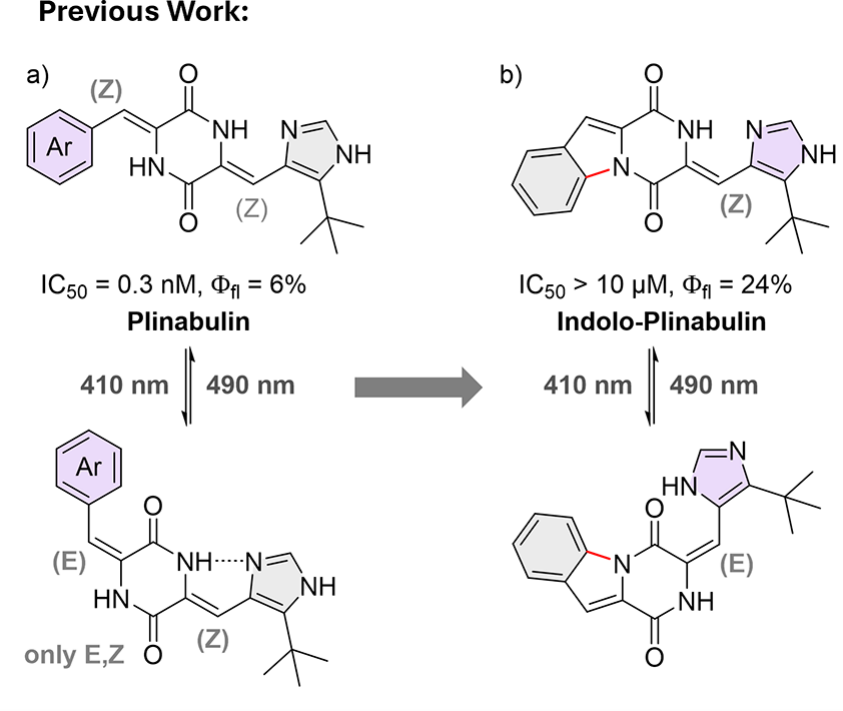
(a) Initial discovery of photoisomerism in plinabulin and derivatives, as investigated in ref 22. (b) σ-bound phenyl substituent on plinabulin to yield indolo-plinabulin.

Contrary to plinabulin, where only the carbocyclic arylidene substituent undergoes isomerization, the “locked” indolo-plinabulin (IndPlin, Figure 1b) exhibited reversible photochromism of the heteroarylidene substituent (93% *E*-isomer with violet light). While the cytotoxic activity of its parent compound was significantly diminished in IndPlin (IC_50_ > 10 μM), its fluorescence quantum yield increased 6-fold (Φ_fl_ = 0.24).^22^ Additionally, the fluorescence intensity could be reversibly modulated by a factor of >3 upon photoisomerization over multiple switching cycles, without any noticeable degradation. These results prompted us to synthesize a small collection of compounds bearing a π-system annulated to the DKP-core structure of HPIs, and to investigate their properties as biocompatible molecular fluorescent photoswitches (Figure 2).

**Figure 2:**
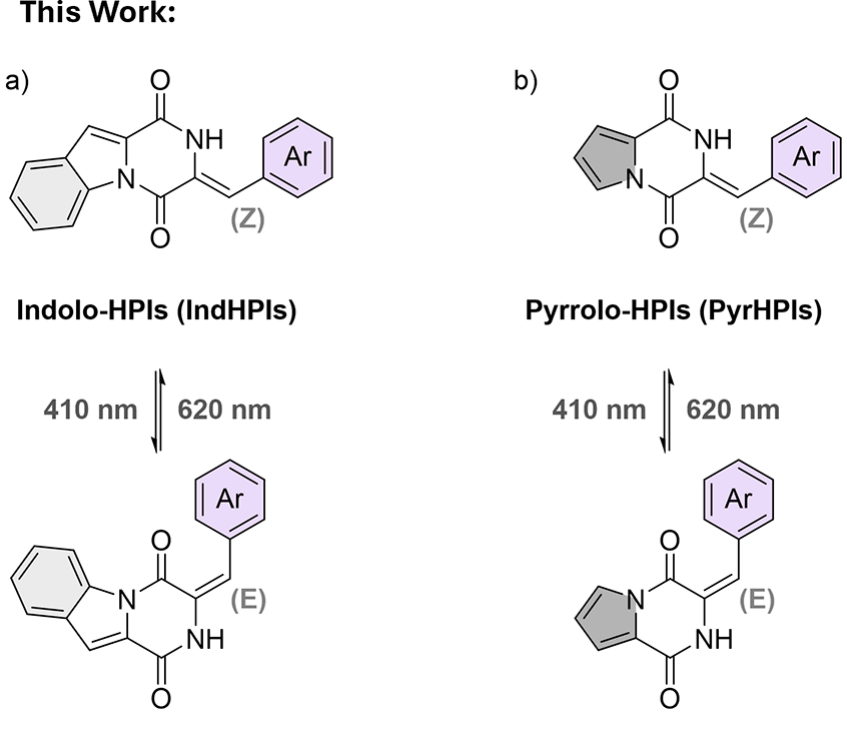
(a) Indolo-hemipiperazines (IndHPIs) and (b) pyrrolo-hemipiperazines (PyrHPIs), both switchable with light of up to 620 nm.

Within this report, we present a collection of elaborated HPI derivatives dubbed indolo-hemipiperazines (IndHPIs, Figure 2a) and pyrrolo-hemipiperazines (PyrHPIs, Figure 2b). The former class of compounds sparked our interest primarily due to intensified luminescence and substantial red shift in absorbance, compared to the previously reported HPIs.^22,23^ To investigate the influence of this annulated π-system on the photophysical properties in more detail, two IndHPIs were selected as reference compounds, and the pyrrolo-analogues were synthesized and subsequently characterized. All of the described compounds exhibit visible-light addressability and in some instances quantitative or excellent isomerization upon illumination with green (523 nm) and red light (620 nm). Furthermore, these compounds, to varying degrees, exhibit strong fluorescence and unusually large Stokes shifts of up to 135 nm. Initial *in vitro* experiments also reveal only limited toxicity of IndHPIs toward HeLa cells at the tested concentrations, as well as efficient cellular uptake. Their luminescent properties are retained within cells, and upon illumination, their fluorescence intensity is reduced and subsequently restored—due to the spontaneous relaxation from the metastable *E*-isomer to the thermally stable *Z*-isomeric form. These results combined render IndHPIs and PyrHPIs attractive for *in vitro* applications, such as switchable fluorescent probes for live super-resolution imaging, as photochromic scaffolds for incorporation in biologically active compounds, or to expand the scope of optical data storage systems. As cyclic dipeptides are common pharmacophores, we hypothesize that HPI-based photoswitchable fluorophores may have the potential to complement the existing palette of dyes for the microscopy of subcellular structures. Additionally, they may be designed into compounds capable of optically self-reporting of their property change (correlated with their isomeric composition).

## Results and Discussion

A small representative collection of carbo- and heterocycle-bearing IndHPIs and PyrHPIs was synthesized in four steps: To generate the diketopiperazine core, standard peptide coupling conditions for the reaction of indole- or pyrrole-2-carboxylic acid with glycine methyl ester hydrochloride were applied (Scheme 1). Cyclization using methanesulfonic acid (MSA) and acetylation with acetic acid anhydride (Ac_2_O) provided the precursor **12**, while the pyrrolo-derivative was cyclized using NaH and acetylation was performed at reduced temperature to generate precursor **13**. Base-catalyzed condensation of the precursors **12**/**13** with aromatic aldehydes generated the respective HPIs **1**–**11** with fair to good yields, using conditions previously developed for HPIs.^23,26^ All compounds were obtained as their pure Z-isomers, as exemplified in the single-crystal structures of IndHPI **2** and PyrHPI **10** (Figures S51 and S52), which is in agreement with earlier reports^22–24^ and has been explained by the Zimmermann–Traxler model.^27^

**Scheme 1:**
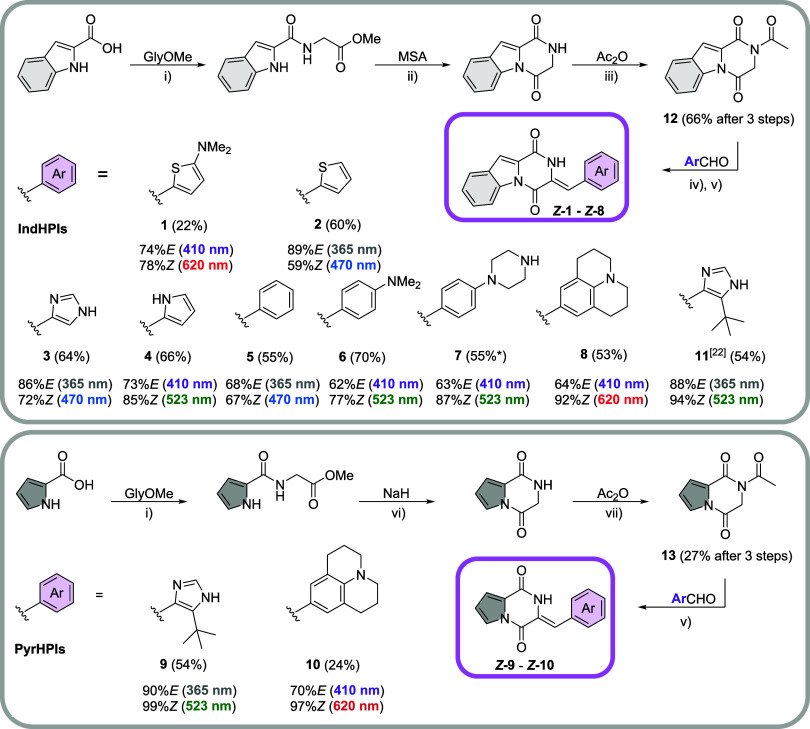
Synthesis of IndHPIs **1**–**8**, **11** and PyrHPIs **9** and **10** ^a^

a The provided yields refer to the last reaction step. (i) EDC·HCl, DMAP, CH_2_Cl_2_, 0 °C to rt, 20 h; (ii) neat, 70 °C, 16 h; (iii) neat, 140 °C, 16 h; (iv) DBU, DMF, rt, 16 h; (v) LiHMDS, THF, −78 °C to rt, 16 h; (vi) THF, 0 °C, 2 h; (vii) neat, 100 °C, 1 h. *After Boc-deprotection in TFA/CH_2_Cl_2_ (1/1), rt, 30 min.

Elemental photophysical properties of compounds **1**–**11** are summarized in Table 1.

**Table 1:**
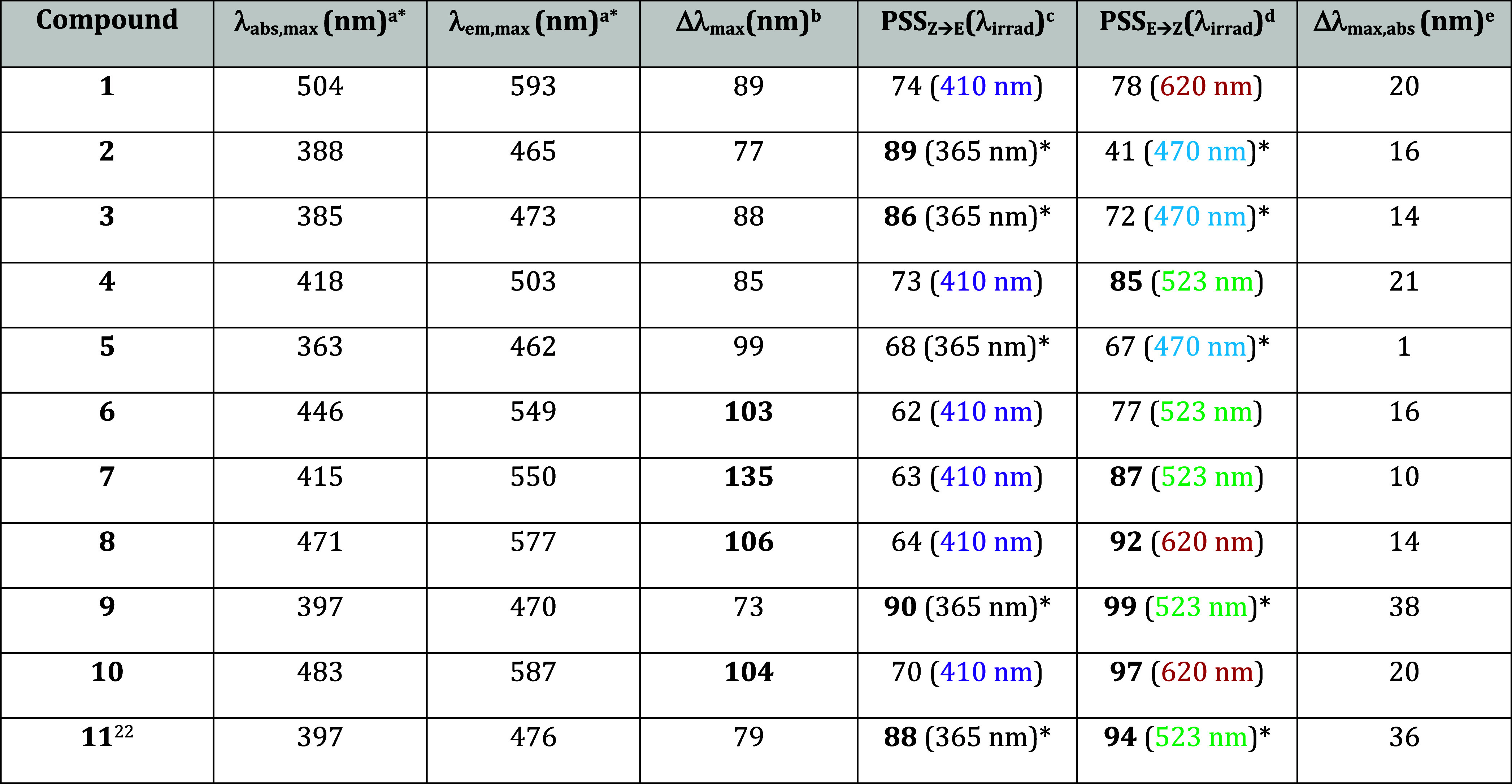
General Photophysical Properties of HPIs **1**–**11** in DMSO

a Absorption and fluorescence maxima.

b Stokes shift.

c PSS with the highest ratio of *E*-isomer upon illumination with λ_irrad_.

d PSS with the highest ratio of *Z*-isomer upon illumination with the indicated λ_irrad_.

e Band separation between the *Z*-isomer and in PSS with the highest *E*-ratio (* with 10.0 equiv of AscH_2_).

All compounds exhibit large Stokes shifts between 85 and 135 nm in DMSO: The absorbance maxima span a range of λ_abs,max_ = 363–504 nm and the emission maxima lie at λ_em,max_ = 462–593 nm. Upon illumination with violet (410 nm) or UV (365 nm) light, all HPIs introduced herein undergo reversible photoisomerization from their stable *Z*-configuration to the metastable *E*-isomer. This isomerization produces mixtures containing 62–90% of the metastable *E*-configuration. In the reverse direction, good to quantitative yields (59–99%) of the *Z*-form can be achieved upon photoequilibration with cyan to red light (490–620 nm). Band separation between the photoisomers is generally enhanced in heterocyclic derivatives compared to the carbocyclic compounds, allowing for higher bidirectional photoconversions, similar to previously reported HPIs^22,23^ (Figure 3a,b).

**Figure 3:**
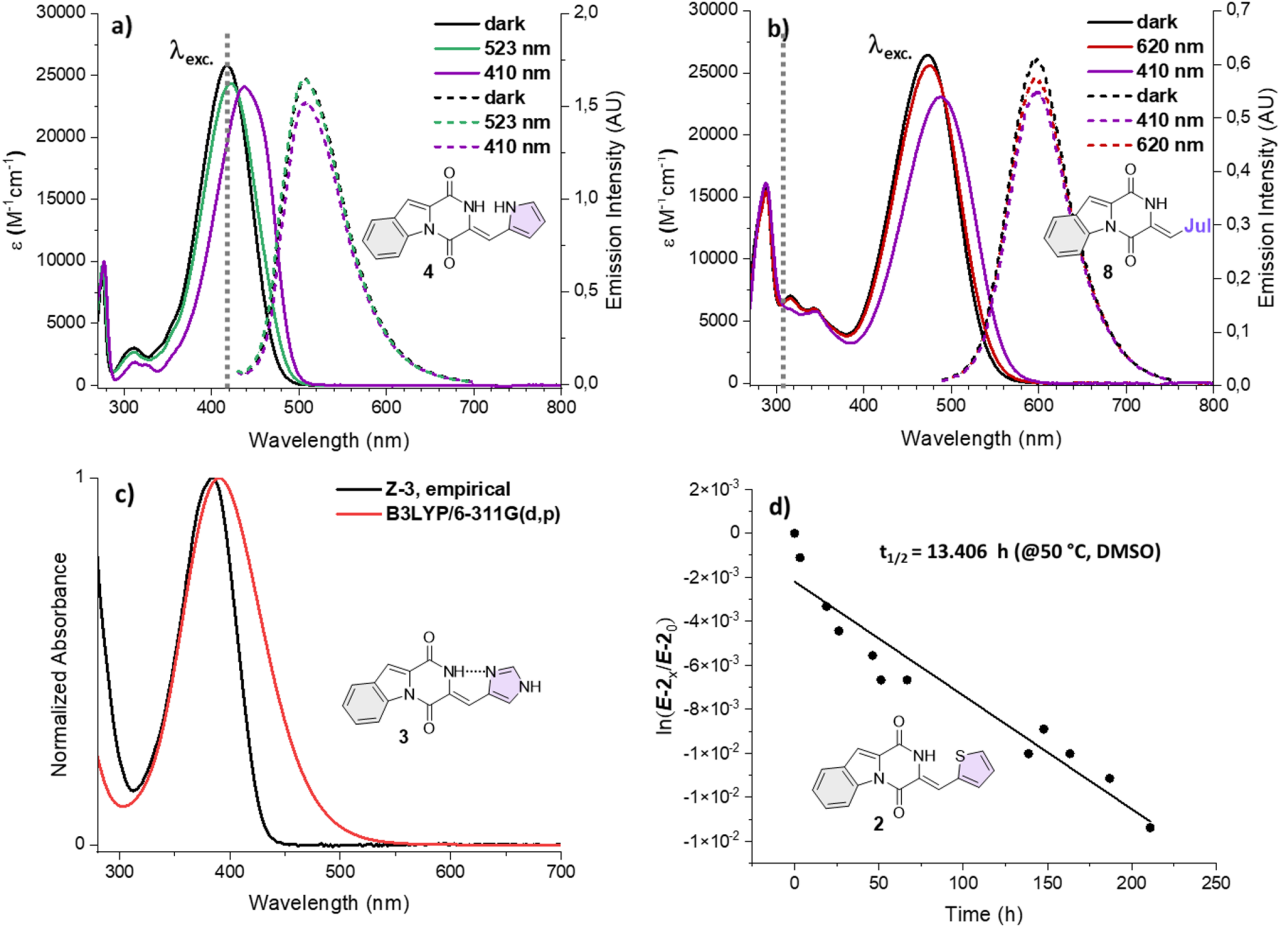
(a) Absorption spectra of compound **4** in DMSO with 10.0 equiv of AscH_2_ prior to irradiation and in the PSS of the indicated wavelengths (solid lines), and the corresponding fluorescence spectra in DMSO (dashed lines). (b) Absorption spectra of compound **8** in DMSO with 10.0 equiv of AscH_2_ prior to and in the PSS of the indicated wavelengths (solid lines), as well as the corresponding fluorescence spectra in DMSO (dashed lines). (c) Comparison of the normalized empirical and simulated (B3LYP/GD3BJ-6-311G­(d,p), PCM­(DMSO)) absorption spectra of compound **3**. (d) Linearized relaxation kinetics of ***E*** **-2** at 50 °C in DMSO, monitored over the course of 200 h, and the resulting thermal half-life *t*_1/2_.

Additionally, the reaction quantum yields for **4** and **8** were determined in both switching directions in DMSO, revealing a substantially higher efficiency for **4** (Φ_R_(*Z*→*E*) = 0.31) compared to **8** (Φ_R_(*Z*→*E*) = 0.13) and previously investigated HPIs.^22,23^ Even more striking is the difference in Φ_R_ for either switching direction, which is about a factor of 30 lower in compound **4** (Φ_R_(*E*→*Z*) = 0.013) and six times lower in compound **8** (Φ_R_(*E*→*Z*) = 0.02).

Next, the UV–vis spectra of **1**–**11** (both isomers) were simulated, after optimizing their structures using the B3LYP-GD3BJ/6-311G­(d,p) PCM (DMSO) level of theory, which had previously^22,23^ been shown to produce theoretical values matching closely to empirical results for other HPIs. Especially the λ_abs,max_ of compounds **3**, **4**, **9**, and **11** are predicted by the calculations with a very high degree of accuracy (Figure 3c and Figure S72), while a slight systematic overestimation by the employed method can be discerned.

Another important characteristic of molecular photoswitches is the thermal stability of their metastable states: While certain switches like most diarylethenes can only be relaxed photochemically (P-type),^28–33^ others exhibit variable thermal lifetimes of their thermodynamically unstable isomers (T-type), which can vary from fractions of milliseconds to years.^34–42^ Hemipiperazines belong to the T-type photoswitches, as the *E-*isomer can relax thermally to the *Z-*form. However, this process is extremely slow in comparison to most established T-type photoswitches, which effectively renders the *E-*isomers thermally metastable under ambient conditions. The prototypical HPI plinabulin showed a lifetime of its *E-*isomer exceeding 1 month at r.t. Therefore, the half-life of HPIs is usually determined at elevated temperatures. We have chosen three compounds to demonstrate general trends in thermal stability of our collection and the influence of intramolecular interactions within respective *E-*isomers. Compound **5** (thermal half-life of 99 h at 50 °C) was chosen as a reference example (no intramolecular interactions) and compared to **4** (H-bonding) or **2** (S–O chalcogen-bonding) (thermal half-life *t*_1/2_ of up to ∼13,000 h at 50 °C) (Figure 3d).

All compounds **1**–**11** further showed a change in their emission intensity upon photoisomerization. This is the net result of two effects: the decrease of Φ_fl_ (significant for compounds **3**, **9**, and **11**) and the decrease of absorption intensity at the selected excitation wavelength (λ_exc._) between the *Z*- and *E-*isomers (dominating for the remaining compounds) (see Table 2). The crucial structural element for a significant Φ_fl_ photomodulation is clearly the internal *H*-bonding between the diketopiperazine HPI core and the photoswitchable arylidene unit. Compounds **3**, **9**, and **11**, all bearing an imidazole moiety, show drastically higher Φ_fl_ than all other HPIs, as well as a more pronounced capability of toggling between a bright *Z*-configuration and a less emissive *E*-isomer (Table 2 and Figure 4). Furthermore, the band shape of the fluorescence spectrum remains unchanged regardless of the isomer composition. One possible interpretation could be a shared excited state of both isomeric forms.^43^ Another is that the *E*-isomers are entirely nonemissive. However, in the latter case, one would expect the decrease of fluorescence intensity to be directly proportional to the decreasing amount of the emissive *Z-*isomer at the respective photoequilibrium, which is not the case.

**Table 2:** Modulation of Φ_fl_ (Significant for Compounds **3**, **9**, and **11**) and the Decrease of Fluorescence Intensity Due to Shift in Absorption between the *Z*- and *E*-Isomers (Dominating for the Remaining Compounds)

compound	**Φ**_**fl**_ **(** *Z* **)** ^a^	**Φ**_**fl,rel**_ **(** *E* **/** *Z* **)** ^b^	CPS_rel_ ^c^
**1**	2.3%	0.81	0.81
**2**	1.5%	0.89	0.82
**3**	**15%**	**0.31**	**0.43**
**4**	1.1%	0.87	0.82
**5**	0.8%	0.95	0.93
**6**	2.4%	0.92	0.91
**7**	0.6%	0.96	0.85
**8**	1.4%	0.93	0.90
**9**	**16%**	**0.33**	**0.16**
**10**	n/d	n/d	n/d
**11** ^22^	**22%**	**0.46**	**0.24**

a Absolute quantum yield (Φ_fl_) of the *Z*-isomers in DMSO.

b Quantum yield relative to Φ_fl_ of the respective *Z*-isomer after equilibration to the PSS with highest *E*-isomer content Φ_fl,rel_.

c Relative fluorescence intensity after equilibration to the PSS with highest *E*-isomer content (CPS_rel_).

**Figure 4:**
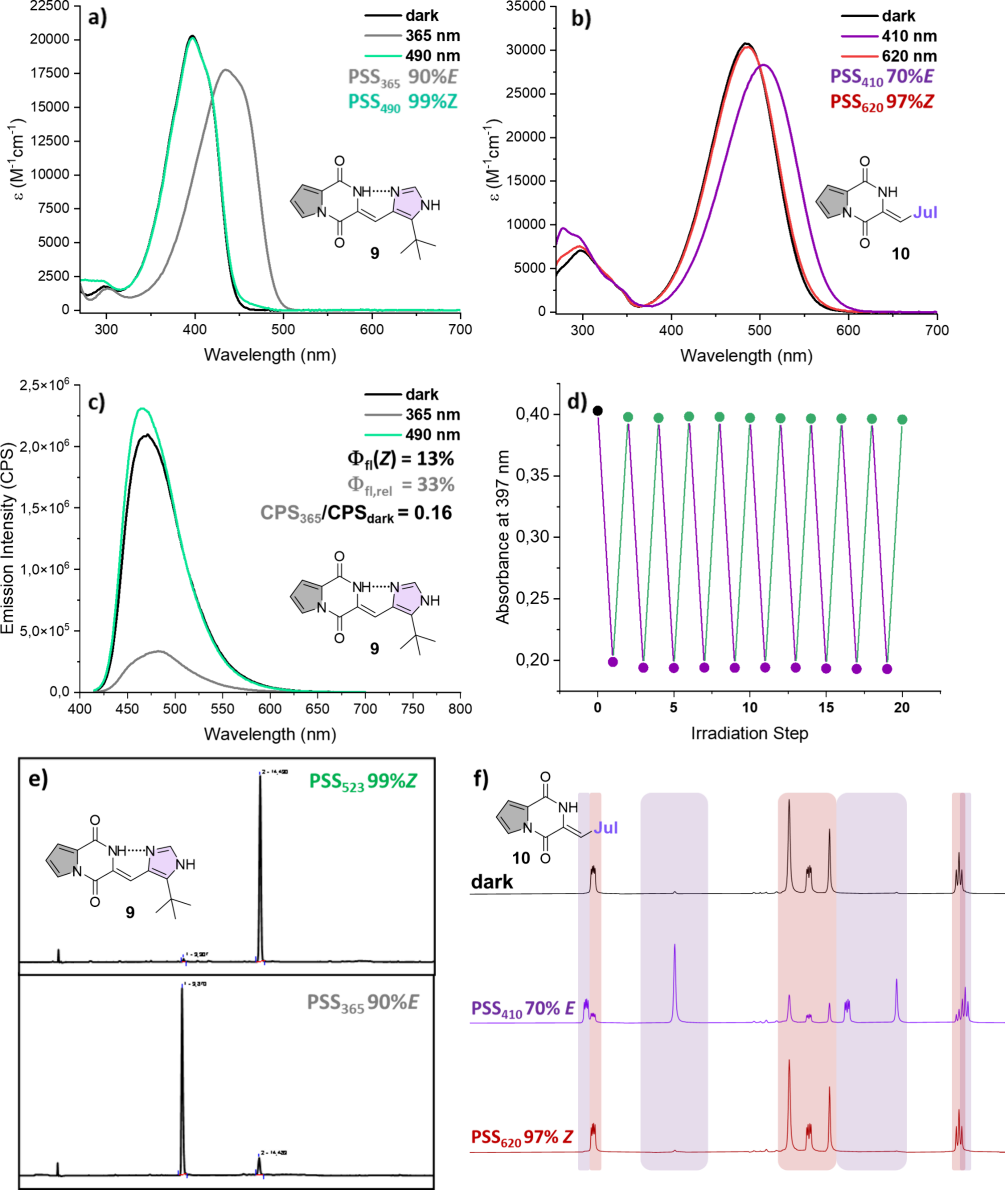
Photophysical properties and thermal stability of PyrHPIs in DMSO with 10.0 equiv of AscH_2_: (a, b) absorption spectra of compound **9** and **10** prior to irradiation and in the PSS of the indicated λ_irrad_; (c) fluorescence spectra of compound **9** prior to illumination and in the PSS of λ_irrad_; (d) switching cycles of compound **9**, achieved with alternating illumination of 365 and 490 nm; (e) HPLC traces of a solution of **9**, first irradiated with 365 nm (bottom) and subsequently with 523 nm (top); (f) ^1^H NMR spectra of compound **10** in DMSO-*d*_6_ prior to illumination and in its PSS.

Simulations and XRD both indicate *H*-bonding within the *Z*-isomer.^22,44^ While internal *H*-bonding within the *E*-isomer has been shown to occur in similar systems^23,45^ and likely is present here, NBO analysis of compound **9** reveals energies of 16.22 kcal/mol for the *Z*-isomer and 11.98 kcal/mol for the *E*-isomer, respectively, indicating a stronger *H*-bond in the native *Z*-isomer. This difference in strength of noncovalent interaction leads to the assumption that the tighter *H*-bound *Z*-isomer assumes a rigidified structure, disfavoring nonemissive relaxation pathways and enhancing luminescence intensity, compared to its *E*-isomer.^46–50^

Overall, particularly compound **9** combines favorable emissive properties (Φ_fl_(*Z*) = 16%) with very efficient bidirectional photoconversions, including quantitative recovery of its *Z*-isomer using green light (Table 1 and Figure 4a,e). Notably, compounds **1**, **8**, and **10** enable for very selective (in case of **10**, near-quantitative) isomerization using 620 nm frequency, which renders them the first reported HPIs that show efficient responsiveness toward red light (Figure 4b). Generally, we can state (by comparing **10** to **8**, or **9** to **11**) that PyrHPIs show somewhat better photoconversions than IndHPIs, due to slightly increased band separation between their photoisomers, which in turn allows for higher selectivity upon illumination.

While the presence of reducing agents is often detrimental to the performance of photoswitches in a biological context, e.g., azobenzenes,^51–53^ HPIs in fact benefit from the reductive environment, which prevents them from slight photooxidative degradation.^22^ Thus, we tested the fatigue resistance of compound **9** in DMSO with 10.0 eq. of ascorbic acid (AscH_2_) upon 10 switching cycles with alternating illumination using 365 and 490 nm light (Figure 4d). The absorption maximum of ***Z*** **-9** at 397 nm was then tracked over the switching cycles and revealed no apparent degradation (*A*_10_/*A*_dark_ = 0.99). The same result was observed (10 switching cycles, 410/590 nm) for compound **1**, with the HPIs having the highest bathochromic shift in absorption and emission (Figure S1). The absorbance maxima of compounds **1**, **4**, and **8** were also monitored upon prolonged irradiation with 410 nm for solutions with or without AscH_2_, revealing the pronounced stabilizing effect of AscH_2_: Some degradation does occur at very long illumination times (up to 30 min; 180-fold equilibration time) for either solution; however, the rate of degradation is up to >700-fold lower when AscH_2_ is added (Figure S8). Combined, these experiments highlight the very pronounced resistance toward irreversible photobleaching, when compared to many well-established fluorophores,^54–57^ and toward degradation by reducing agents, when compared to azobenzenes,^51–53^ in particular. Interestingly, upon further stability testing of the previously reported indolo-plinabulin (**11**), we observed that the type of photochromism depends on the used concentration of AscH_2_: In a saturated solution of AscH_2_ in DMSO, **11** exhibited negative photochromism upon illumination with violet light, as mentioned in the original publication.^22^ As a result, illumination with longer wavelengths to recover the *Z*-configuration was found to be inefficient and equilibrated to merely 33% ***Z*** **-11**.

Upon limiting the content of AscH_2_ to 10.0 equiv., however, a pronounced positive photochromism was observed for **11** when irradiated with UV or violet light (Figure S2). As a consequence, the efficiency of backisomerization significantly increased (up to 94% of ***Z**-* **11** with 523 nm).

Finally, we were interested in whether the fluorescent HPIs can be used inside living cells. HeLa cells were therefore treated with solutions of the compounds, screened for their potential as photoswitchable fluorophores. First, suitable candidates were selected based on their absorption properties, to exclude harmful UV-light exposure of the cells. Consequently, the compounds **1**, **4**, **7**, **8**, **9**, and **10** were chosen based on their efficient isomerization with violet light. While most were internalized and showed fluorescence within the cells, only compounds **4** and **8** exhibited reversible fluorescence modulation upon irradiation. Compound **9** could not be detected within the cells to a significant degree. Regarding compounds **1**, **7**, and **10**, we did not detect any meaningful fluorescence recovery upon irradiation with 405 nm and subsequent relaxation in the dark. Possible explanations for these observations include instability of these compounds toward the illumination in aqueous media, poor cellular uptake (in the case of compound **9**), and—in the case of compounds **1**, **7**, and **10**—longer thermal half-life of the metastable isomer, or altered reactivity toward cellular components.

The cells containing compounds **4** and **8** were irradiated with the 405 nm laser of a confocal microscope, to generate the respective *E*-isomer. Upon irradiation, indeed, a decrease in signal intensity could be observed, surprisingly to a higher extent than was previously determined in solution (Figure S29). To test the reversibility of the turn-off effect upon *E*-isomer enrichment, the cells were illuminated a second time—now with 505 nm, which should primarily produce the stronger fluorescent *Z*-isomer (Figure S29). The control experiment with no additional illumination, however, yielded spontaneous regeneration of the fluorescent signal at roughly the same rate as that of the irradiated samples.

To examine this unexpected signal regeneration further, thermal relaxation of the *E*-isomers of **4** and **8** in living HeLa cells was monitored over 15 min post-illumination with 405 nm, revealing rapid fluorescence recovery within minutes (Figure 5a and Figure S32).

**Figure 5:**
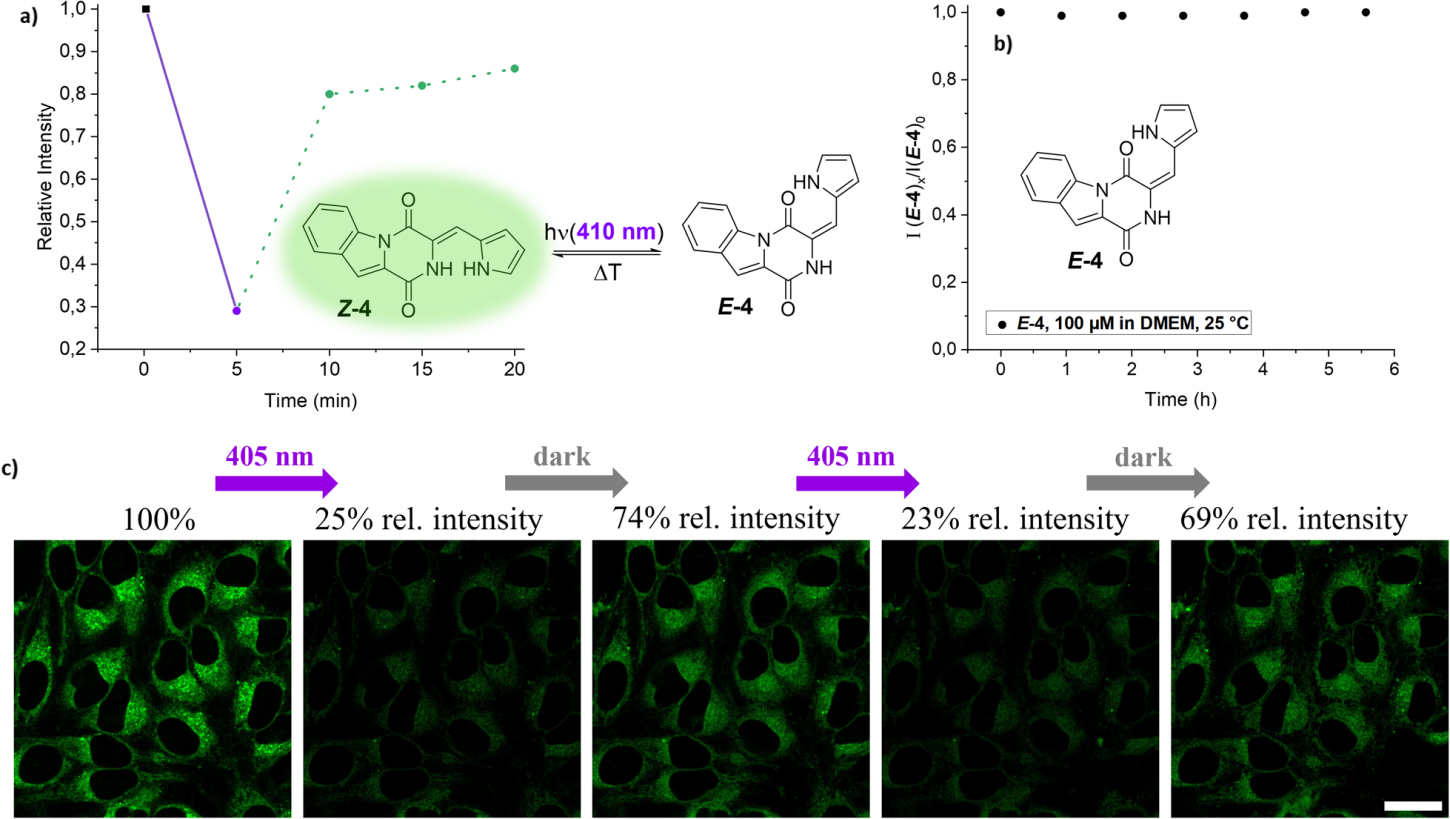
(a) Fluorescence emission intensity inside of HeLa cells after 5 min of illumination with 405 nm and in defined time intervals after the illumination phase. (b) Thermal isomerization kinetics of ***E*** **-4**, 100 μM in DMEM at 25 °C, monitored via HPLC over the course of 5.5 h. (c) HeLa cells with internalized ***Z*** **-4** prior to illumination (left), after irradiation with 405 nm for 5 min (middle), and subsequent relaxation phase of 5 min under no irradiation (right). The emission intensities are stated relative to the initial value of the nonilluminated cells (scale bar: 25 μm).

Considering the long thermal half-lives of the compounds in question, previously determined in DMSO, these results were quite unexpected (Figure 3d). Testing whether relaxation kinetics in the cell medium would be different to those in DMSO, a stock solution of ***Z*** **-4** in DMSO was illuminated and subsequently diluted in DMEM. The *E*-isomer content was monitored over 5.5 h at rt; however, no re-isomerization could be detected (Figure 5b).

To test whether regeneration of the luminescent signal may be a result of re-isomerization and no other process inside of the cells like irreversible metabolization, a second experiment was conducted: The cells with internalized IndHPIs **4** and **8** were illuminated with 405 nm for 5 min, which brought about a significant decrease in fluorescence intensity of up to 25%. Upon equilibration in the dark for 5 min, regeneration of up to 74% of the initial intensity could be achieved (Figure 5c). A second cycle of irradiation and thermal relaxation could be performed, yielding virtually the same signal intensities, proving the reversible nature of the process and thus the causal relationship of photoisomerization and modulation of luminescence.

A potential effect of pH in different cell compartments on the isomerization of IndHPIs was additionally investigated: Compound ***Z*** **-4** was illuminated in aqueous solutions containing the respective buffers to establish pH values of 5.5, 7.5, and 8.5, as well as 10 vol % of DMSO in each case, to help solubilization. No difference in isomeric composition was discernible, as monitored *via* HPLC (Figure S22). While relaxation of ***E*** **-4** was found to be accelerated at more acidic pH, the rate was still found to be decidedly too slow to explain the spontaneous re-isomerization demonstrated inside of cells.

In order to further assess the scope of applicability of the compounds **4** and **8** inside living cells, we have performed viability assays on HeLa cells and on another mammalian cell line (HepG2). In all these tests, we did not observe a significant decrease of cell viability (<80%) below the 2 μM concentration of both compounds in neither cell line. Compound **4** shows more significant cytotoxicity at and above the 5 μM concentration for both cell lines, and compound **8** becomes slightly more cytotoxic (but only to HeLa cells) at the 10 μM concentration.

In an effort to demonstrate the photostability of IndHPIs, we tried to simulate the high illumination intensities employed in superhigh-resolution microscopy: Compounds **4** and **8** were subjected to the prolonged irradiation of up to 30 min with 410 nm LED light (3 W)—the frequency used for photoisomerization inside the HeLa cells. We have observed a meaningful degradation within 10 min of irradiation. Yet, the degradation was almost fully suppressed (<5%) in the presence of AscH_2_, which acted as a reducing agent preventing photooxidation of both compounds (Figures S8 and S9). Therefore, we believe that the photobleaching in cytosol will be negligible as well due to the high concentrations of glutathione. We also tested the harshest conditions for illumination achievable with our LED setup: Irradiation with 365 nm LEDs of 2 × 9 W of electrical power led to significant degradation for compound **4** over the course of 5 min (>30-fold equilibration time) in plain DMSO and to only minor degradation over the course of 30 min when AscH_2_ was added (*A*_30min_/*A*_10s_ = 0.94) (Figure S10). Compound **8** similarly exhibited highly increased photostability upon addition of AscH_2_, but to a slightly lesser extent than compound **4** (*A*_30min_/*A*_10s_ = 0.76) (Figure S11).

Finally, addressing the unexpectedly fast reisomerization kinetics of our compounds inside of cells, we turned to the literature: In a precedence study, the working group around Tolbert proposed a specific mechanism for the thermal relaxation of the metastable isomer of the GFP chromophore, which is structurally very similar to our HPI system.^58^ By addition of a suitable nucleophile to a Michael-acceptor functionality (also present within HPIs), the π-bond character of the central double bond within the chromophore is reduced, facilitating rotation around that same bond and thus resulting in regeneration of the stable isomer upon elimination of the initial nucleophile (Figure 6a). An adequate species within cells would be represented in GSH, as it occurs inside cells in high concentrations of roughly 1–10 mM.^59–61^ Furthermore, as a soft nucleophile, GSH exhibits high β-site selectivity within Michael systems.^62^ To address this hypothetical analogy to the studies of Tolbert and colleagues, compound ***Z*** **-4** was subjected to the following experiment: A solution of ***Z*** **-4** was prepared in DMEM, also containing 2 mM GSH. The solution was split in two parts, one of which was used as a blank sample and the other was equilibrated to its PSS with 410 nm illumination. Immediately afterward, a spectrum was recorded every 30 s for 15 min, while the temperature of the sample was kept at 37.5 °C (Figure 6b). The resulting spectra highlight the strikingly different relaxation behavior of compound **4** in the presence of a soft nucleophile: Within 15 min, the sample almost completely reverted to the *Z*-isomer (which would equate to baseline reading), while in DMEM without GSH, no detectable isomerization took place over several hours (see Figure S34b). These findings are additionally supported by HPLC experiments (Figure S34c). Combined, these results readily explain the observed spontaneous fluorescence regeneration shown in Figure 5a,c and additionally provide a reason why no difference in initial fluorescence signal was exhibited when the cells were treated with either *Z*- or the *E*-isomers of the compounds. Compounds **1**, **7**, and **10** were internalized by cells but most likely underwent photobleaching upon irradiation, as the fluorescence level irreversibly decreased upon illumination.

**Figure 6:**
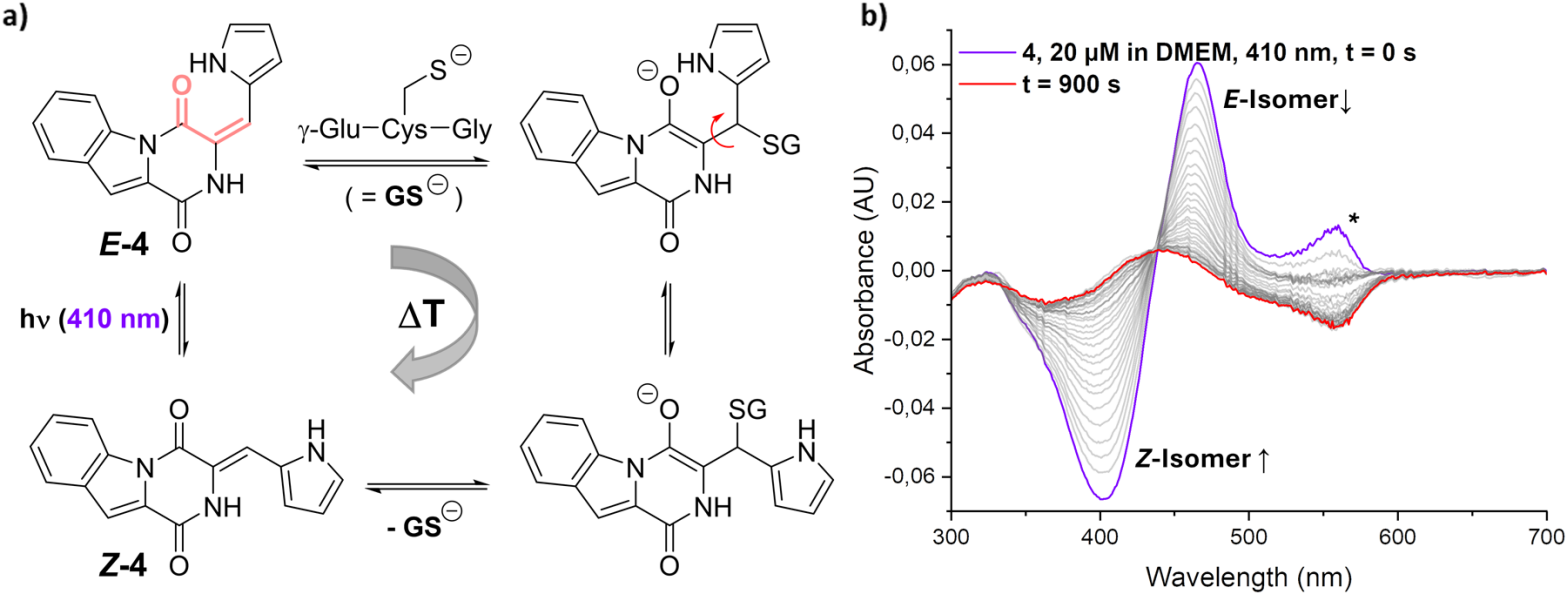
(a) Hypothesized mechanism of thermal reisomerization of IndHPIs, mediated by GSH, in analogy to ref 57. Michael acceptor highlighted in pale red. (b) UV–vis spectra of compound **4**, 20 μM in DMEM, 410 nm, t = 0 s,containing 2 mM of GSH, after irradiation to the PSS at 410 nm and blanked against pure ***Z*** **-4** in the same solution. Every 30 s, a spectrum was recorded over the course of 15 min total. *Artifact, likely caused by photobleaching of phenol red, present in DMEM.

## Conclusions

To conclude, within this work, we present the synthesis and detailed photophysical investigation of a novel class of photoswitchable fluorophores: This family of compounds comprises HPIs with annulated π-systems (either indole- or pyrrole-annulated cores), which exhibit up to quantitative isomerization capabilities upon illumination with visible wavelengths, in some instances even with biocompatible red light. An intramolecular *H*-bond between the DKP core and the arylidene unit was identified as a defining structural feature for significant fluorescence quantum yields. Compounds containing an imidazole moiety, capable of such a noncovalent interaction, further revealed a large band separation between the photoisomers, as well as efficient modulation of their emission intensity upon photoisomerization. Upon excitation, all fluorescent HPIs presented herein exhibit unusually large Stokes shifts of up to 135 nm, which is a highly favorable property for fluorophores used in biological applications. General photophysical properties of these compounds are largely unaffected by the size of the π-system attached to the DKP core, highlighting a strong potential for structural diversification without compromising their highly beneficial switching capabilities. IndHPIs further exhibit remarkable thermal half-lives on the order of years in DMSO in their metastable isomeric form, even at elevated temperatures. This allows for the isolation of the metastable isomers and virtually indefinite storage in their solid form. Additionally, it was shown that their absorption properties can be simulated to a high degree of accuracy using the B3LYP/6-311G­(d,p) level of theory.

Initial cell experiments revealed a seemingly unspecific cellular uptake of these fluorophores. Furthermore, the fading and regeneration of fluorescence upon illumination (of up to 75% in either direction) could be shown in two selected examples. An unexpected thermal regeneration of fluorescence intensity after irradiation was observed, an effect that could be replicated outside of living cells with the addition of GSH. This elucidates a novel, nucleophile-mediated relaxation pathway of HPIs from their metastable isomeric state, which will undoubtedly be of high relevance for the future design of novel HPIs. While the ability of IndHPIs to modulate the fluorescence intensity inside of cells has been shown to be limited in scope, it is worth noting that for the first time, photoisomerization of HPIs within living cells can be followed in real time.

Lastly, it needs to be underscored that IndHPIs exhibit several attractive features, in particular for application in biological contexts: starting from their resistance against reductive degradation, limited toxicity, and most importantly their highly favorable photophysical properties, such as responsiveness to light >600 nm. These characteristics in sum address several shortcomings of established molecular photoswitches and highlight the potential of this novel class of photochromic compounds.

## Supplementary Material





## Notes

Details of synthesis, structural analyses, photochemical, photophysical, thermal stability, computational data, and biological experiments (PDF)
Addition of GSH influences the rate of relaxation of the compound **4** (MP4)

## Abbreviations

AscH2: ascorbic acid
GSH: glutathione (reduced)
HPI: hemipiperazine
IndHPI: indolo-hemipiperazine
IndPlin: indolo-plinabulin
PSS: photostationary state
PyrHPI: pyrrolo-hemipierazine
PyrPlin: pyrrolo-plinabulin
