# Supplementary material for: Photoswitchable Fluorescence of Peptide-Based Hemipiperazines Inside of Living Cells
Source: J Am Chem Soc. 2025 Jul 14;147(30):26652–62. doi: 10.1021/jacs.5c07013 (PMC12314918; doi:10.1021/jacs.5c07013)
Supplement: Supplementary file 1 [file ja5c07013_si_001.pdf]

Photoswitchable Fluorescence of Peptide-Based Hemipiperazines Inside of Living Cells

Peter Gödtel[1,4,] Anna Rösch[2], Susanne Kirchner[1], Rabia Elbuga-Ilica[1], Angelika Seliwjorstow[1], Olaf Fuhr[3], Ute Schepers[1,2*], Zbigniew L. Pianowski[1,4*]

[1] Institute of Organic Chemistry, Karlsruhe Institute of Technology
76131 Karlsruhe, Germany.

[2] Institute of Functional Interfaces, Karlsruhe Institute of Technology
76344 Eggenstein-Leopoldshafen, Germany.

[3] Institute of Nanotechnology and Karlsruhe Nano Micro Facility (KNMFi), Karlsruhe Institute of Technology; 76344 Eggenstein-Leopoldshafen, Germany.

[4] Institute of Biological and Chemical Systems – FMS, Karlsruhe Institute of Technology
76344 Eggenstein-Leopoldshafen, Germany.

Supplementary Information

Table of Contents

Synthetic Procedures and Characterization 2
Irradiation Intensities of LEDs 13
Absorption and Emission Spectra of the Photostationary States 14
Photostability 18
Quantification of Photostationary States 24
Influence of pH on Isomerization Ratios 32
Thermal Stability of *E*-Isomers 33
Isomerization Quantum Yield 34
Cell cultures 37
In Vitro Control Experiments for Thermal Relaxation 44
Normalized UV-Vis and Fluorescence Spectra 46
Fluorescence Quantum Yield 49
NMR Spectra 50
Crystal Structure Determinations 63
Theoretically Obtained Geometries 65
Molecular Orbitals 92
Excitation Energies and Calculated Absorption Spectra 104
Calculated Absorption Spectra 118
Natural Bond Orbital (NBO) Analysis 121

Synthetic Procedures and Characterization

Synthesis of diketopiperazine-precursors **12 (IndDKP)** and **13 (PyrDKP)**

Synthesis of methyl (1H-indole-2-carbonyl)glycinate

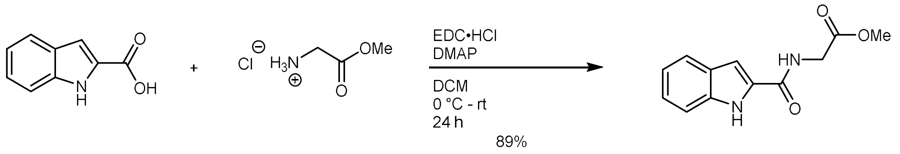

To a solution of 1H-indole-2-carboxylic acid (5.00 g, 31.0 mmol, 1.00 eq.) and glycine methyl ester hydrochloride (4.67 g, 37.2 mmol, 1.20 eq.) in 125 mL dry DCM, EDC hydrochloride (7.14 g, 37.2 mmol, 1.20 eq.) and DMAP (6.06 g, 49.6 mmol, 1.60 eq.) were added at 0 °C. After stirring for 4 h at 0 °C, the solution was allowed to warm to ambient temperature and stirred for an additional 20 h. The reaction mixture was then cooled in an ice bath once more and the resulting precipitate was filtered off. The remaining organic layer was washed with distilled water and 10% HCl, dried over Na_2SO_4 and all volatiles were evaporated *in vacuo*. The precipitate was combined with the extracted crude solid and purified by recrystallization from MeOH, to yield 7.19 g of a colorless solid (89%).

^{1}H NMR (400 MHz, DMSO): δ = 11.61 (bs, 1H), 8.93 (t, *J* = 6.0 Hz, 1H), 7.64 - 7.62 (m, 1H), 7.44 - 7.42 (m, 1H), 7.21 - 7.17 (m, 1H), 7.16 - 7.15 (m, 1H), 7.06 - 7.02 (m, 1H), 4.06 (d, *J* = 6.0 Hz, 2H), 3.67 (s, 1H) ppm.

^{13}C NMR (101 MHz, DMSO): δ = 170.5, 161.5, 136.5, 131.0, 127.0, 123.5, 121.6, 119.8, 112.3, 103.0, 51.7, 40.8 ppm.

TLC: R_f = 0.3 (Cyclohexane:EtOAc; 10:3).

IR (ATR): ν = 3367 (m), 3271 (vs), 3194 (w), 3153 (w), 3132 (w), 3084 (w), 3055 (w), 3043 (w), 3027 (w), 2996 (w), 2946 (w), 1737 (vs), 1639 (vs), 1621 (s), 1577 (m), 1548 (vs), 1511 (m), 1497 (w), 1449 (w), 1438 (m), 1421 (s), 1409 (s), 1370 (m), 1344 (w), 1315 (s), 1275 (s), 1215 (vs), 1184 (s), 1159 (m), 1140 (s), 1116 (m), 1077 (w), 1023 (w), 1001 (w), 983 (m), 949 (w), 932 (w), 849 (w), 822 (vs), 781 (vs), 773 (s), 747 (vs), 730 (vs), 703 (s), 628 (s), 611 (m), 585 (w), 558 (vs), 538 (s), 469 (s), 441 (vs), 375 (s) cm^{-1}.

HRMS (EI): *m/z* calcd. for C12H12N2O3 [M+H], 232.0922; found, 232.0921.

Synthesis of 2,3-dihydropyrazino[1,2-a]indole-1,4-dione

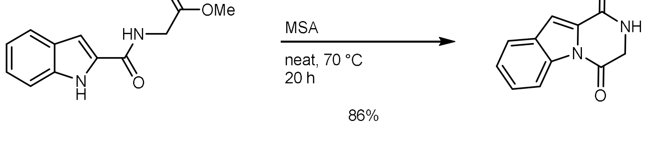

A mixture of methyl (1H-indole-2-carbonyl)glycinate (1.00 g, 3.81 mmol, 1.00 eq.) and MSA (17.0 mL, 25.2 g, 68.8 eq.) was stirred overnight at 70 °C. The reaction mixture was then poured on ice-cold water and the resulting precipitate was filtered off to afford the product as a colorless solid (860 mg, 86%).

^{1}H NMR (400 MHz, DMSO): δ = 8.49 (s, 1H), 8.37 (dd, J = 8.3, 1.0 Hz, 1H), 7.80 (d, J = 7.9 Hz, 1H), 7.52 (ddd, J = 8.5, 7.3, 1.4 Hz, 1H), 7.41 (td, J = 7.6, 1.2 Hz, 1H), 7.36 (s, 1H), 4.42 (d, J = 2.1 Hz, 2H) ppm.

^{13}C NMR (101 MHz, DMSO): δ = 163.5, 156.4, 134.2, 129.9, 128.5, 127.2, 124.8, 122.5, 115.7, 111.8, 46.7 ppm.

TLC: R_f = 0.13 (DCM:EtOAc; 1:1).

IR (ATR): ν = 3390 (vw), 3367 (vw), 3350 (vw), 3322 (vw), 3308 (vw), 3291 (vw), 3284 (vw), 3275 (vw), 3267 (vw), 3254 (vw), 3245 (vw), 3170 (w), 3129 (w), 3102 (w), 3041 (w), 3034 (w), 2952 (w), 2911 (w), 2873 (w), 2825 (w), 2751 (vw), 2738 (vw), 1703 (vs), 1672 (vs), 1605 (m), 1592 (vs), 1574 (s), 1499 (w), 1477 (w), 1434 (vs), 1417 (s), 1380 (vs), 1356 (s), 1332 (vs), 1309 (m), 1298 (m), 1259 (w), 1241 (w), 1203 (m), 1187 (w), 1153 (m), 1132 (s), 1109 (m), 1079 (s), 1037 (m), 1010 (w), 986 (w), 953 (w), 919 (w), 898 (w), 867 (w), 841 (s), 823 (s), 752 (vs), 73 (vs), 693 (m), 653 (m), 623 (w), 609 (w), 595 (w), 571 (m), 538 (vs), 484 (s), 469 (vs), 433 (s), 412 (m), 398 (m) cm^{-1}.

HRMS (EI): *m/z* calcd. for C11H8N2O2 [M+H], 200.0580; found, 200.0580.

Synthesis of 2-acetyl-2,3-dihydropyrazino[1,2-a]indole-1,4-dione (**12**)

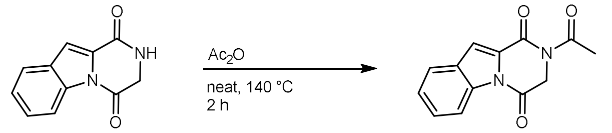

2,3-Dihydropyrazino[1,2-a]indole-1,4-dione (1.00 g, 5.00 mmol, 1.00 equiv.) was dissolved in acetic anhydride (100 mL, 108 g, 1.08 mol, 216 equiv.) and refluxed at 140 °C for 2 hours. The solvent was removed under reduced pressure and the product was washed with diethyl ether and filtered off to yield 1.15 g (4.75 mmol, 95%) of the product as a golden metallic solid.

^{1}H NMR (400 MHz, DMSO): δ = 8.35 (d, J = 8.3 Hz, 1H), 7.87 (dt, J = 7.9, 1.0 Hz, 1H), 7.70 (s, 1H), 7.60 (ddd, J = 8.4, 7.2, 1.3 Hz, 1H), 7.46 (td, J = 7.6, 7.2, 1.1 Hz, 1H), 4.69 (s, 3H), 2.58 (s, 3H) ppm.

^{13}C NMR (101 MHz, DMSO): δ = 171.7, 162.1, 156.9, 134.2, 129.2, 128.7, 128.4, 125.2, 123.1, 115.7, 115.6, 48.9, 27.2 ppm.

TLC: R_f = 0.30 (Cyclohexane:EtOAc; 10:3).

IR (ATR): ν = 1720 (vs), 1696 (vs), 1679 (vs), 1606 (w), 1557 (s), 1436 (s), 1404 (vs), 1370 (s), 1360 (vs), 1336 (vs), 1266 (vs), 1234 (s), 1210 (vs), 1160 (s), 1139 (s), 1115 (m), 1099 (s), 1069 (m), 1044 (m), 1010 (m), 979 (m), 967 (s), 948 (m), 878 (m), 853 (s), 839 (m), 800 (w), 755 (vs), 739 (vs), 694 (m), 670 (m), 642 (w), 618 (m), 602 (m), 585 (m), 575 (m), 550 (m), 537 (s), 499 (w), 484 (w), 448 (w), 432 (s), 407 (w), 388 (w), 375 (m) cm^{-1}.

HRMS (EI): *m/z* calcd. for C13H10N2O3 [M+H], 242.0686; found, 242.0687.

Synthesis of methyl (1H-pyrrole-2-carbonyl)glycinate

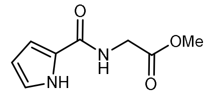

Synthesized according to general procedure B. The crude product was obtained as a colorless solid (67%).

^{1}H NMR (400 MHz, DMSO): δ = 11.47 (s, 1H), 8.42 (t, *J* = 6.0 Hz, 1H), 6.90 – 6.84 (m, 1H), 6.82 – 6.77 (m, 1H), 6.12 – 6.07 (m, 1H), 3.96 (d, *J* = 6.0 Hz, 2H), 3.64 (s, 3H) ppm.

^{13}C NMR (101 MHz, DMSO): δ = 170.7, 160.9, 125.6, 121.7, 110.4, 108.7, 51.7 ppm.

TLC: R_f = 0.3 (Cyclohexane:EtOAc; 10:3).

HRMS (EI): *m/z* calcd. for C8H10N2O3 [M], 182.0691; found, 182.0691.

<u>2,3-dihydropyrrolo[1,2-a]pyrazine-1,4-dione</u>

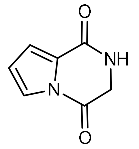

Methyl-(1H-pyrrole-2-carbonyl)glycinate (1.00 eq.) was dissolved in anhydrous THF (0.05 M) and cooled to 0 °C. Subsequently, NaH (60 wt% in mineral oil) was added and the mixture was stirred at 0 °C under an Ar atmosphere for 5 min. Afterwards, the ice-bath was removed and left to stir for another 2 h, an aq. sat. NH_4Cl solution was added, and the aqueous phase was quickly extracted using EtOAc. The combined organic layers were dried over Na_2SO_4, the solvent was evaporated *in vacuo* and the resulting crude solid was dried under reduced pressure and used without further purification. The yield was 63% of a colorless solid.

^{1}H NMR (400 MHz, DMSO): δ = 8.18 (s, 1H), 7.58 (dd, *J* = 3.2, 1.6 Hz, 1H), 6.94 (dd, *J* = 3.4, 1.6 Hz, 1H), 6.57 (t, *J* = 3.3 Hz, 1H), 4.39 (d, *J* = 2.1 Hz, 3H) ppm.

^{13}C NMR (101 MHz, DMSO): δ = 162.8, 155.9, 126.4, 118.7, 116.6, 114.7, 46.4 ppm.

HRMS (EI): *m/z* calcd. for C7H6N2O2 [M], 150.0429; found, 150.0429.

<u>2-acetyl-2,3-dihydropyrrolo[1,2-a]pyrazine-1,4-dione (**13**)</u>

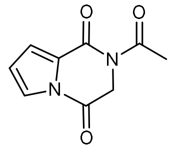

2,3-dihydropyrrolo[1,2-a]pyrazine-1,4-dione (1.00 equiv.) was dissolved in acetic anhydride (50 mM) and stirred for 1 h at 100 °C. After TLC showed no further change in composition, most of the acetic anhydride was removed *in vacuo* and subsequently, the remaining organic phase was diluted with CH_2Cl_2 and washed with distilled water and brine. The organic layer was then dried over Na_2SO_4, filtered off and the volatiles were removed under reduced pressure to afford the crude product. Purification ensued employing flash column chromatography (CH / EtOAc : 8/2) to afford the pure product as yellow crystals (64%).

^{1}H NMR (400 MHz, DMSO): δ = 7.73 (dd, *J* = 3.1, 1.6 Hz, 1H), 7.27 (dd, *J* = 3.5, 1.6 Hz, 1H), 6.69 (t, = 3.4 *J* Hz, 1H), 4.68 (s, 2H), 2.53 (s, 3H) ppm.

^{13}C NMR (101 MHz, DMSO): δ = 171.7, 161.4, 155.9, 125.6, 120.5, 120.3, 116.0, 48.7, 27.2 ppm.

TLC: R_f = 0.3 (CH / EtOAc: 8:2).

IR (ATR): ν = 3145 (w), 3135 (w), 3123 (w), 3019 (vw), 2985 (w), 2953 (w), 2918 (w), 2853 (w), 1737 (s), 1687 (vs), 1561 (s), 1540 (w), 1449 (s), 1435 (s), 1415 (vs), 1366 (vs), 1354 (vs), 1332 (vs), 1254 (vs), 1235 (vs), 1211 (vs), 1157 (s), 1116 (vs), 1072 (vs), 1051 (s), 1041 (vs), 1009 (m), 980 (m), 960 (s), 915 (m), 884 (m), 873 (w), 856 (m), 786 (w), 761 (vs), 738 (vs), 681 (w), 618 (vs), 591 (m), 537 (s), 479 (m), 411 (w), 384 (w) cm^{-1}.

HRMS (EI): *m/z* calcd. for $C_9H_8N_2O_3$ [M+H], 192.0529; found, 192.0529.

Synthesis of Indolo-Hemipiperazines (**IndHPIs**) and Pyrrolo-Hemipiperazines (**PyrHPIs**)

General Procedure

300 mg (1.24 mmol, 1.00 eq.) of 2-acetyl-2,3-dihydropyrazino[1,2-a]indole-1,4-dione (**12**) (or 2-acetyl-2,3-dihydropyrrolo[1,2-a]pyrazine-1,4-dione (**13**)) were dissolved in dry DMF (0.20 M) under an argon atmosphere. The respective aldehyde (1.20 eq.) was dissolved in the mixture and DBU (1.10 eq.) was added. The mixture was stirred for 16-18 h under an argon atmosphere at room temperature. The reaction mixture was subsequently poured on ice-cold water (10 times the volume of DMF) and the resulting precipitate was filtered off. The crude product was finally purified either *via* recrystallization or column chromatography (please check the respective entries for details).

(Z)-3-((5-(dimethylamino)thiophen-2-yl)methylene)-2,3-dihydropyrazino[1,2-a]indole-1,4-dione (**1**)

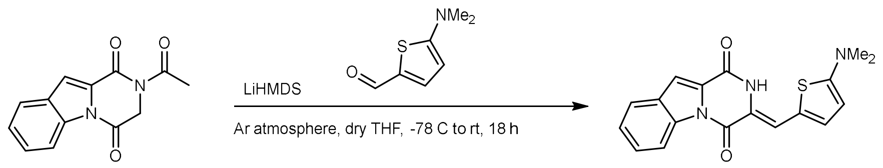

The following procedure was adapted from [1].

2-acetyl-2,3-dihydropyrazino[1,2-a]indole-1,4-dione (**12**) (300 mg, 1.24 mmol, 1.00 eq.) was dissolved in dry THF (0.20 M) under an argon atmosphere and cooled to -78 °C. In a separate flask, 5-(dimethylamino)thiophene-2-carbaldehyde (231 mg, 1.49 mmol, 1.20 eq.) was dissolved in 1 mL of dry THF. 1.61 mL of a 1 M solution of LiHMDS (260 mg, 1.61 mmol, 1.30 eq.) was added to the diketopiperazine solution of **12** and the mixture was stirred for 5 min. Subsequently, the aldehyde was added over the course of 5 min *via* syringe. The flask and the syringe used for transfer were rinsed with an additional 1 mL of dry THF. The reaction mixture was then left to stir for an additional 20 min and subsequently transferred to an ice-bath and left to warm to rt overnight. Afterwards, the mixture was poured into ice-cold water and the resulting precipitate was fitered off, washed with cold water and finally recrystallized from MeCN to afford the pure product as a deep-purple solid (94 mg, 22%).[1]

^{1}H NMR (400 MHz, $CDCl_3$): δ = 8.66 (d, J = 9.3 Hz, 1H), 7.96 (bs, 1H), 7.75 (d, J = 7.9 Hz, 1H), 7.57 – 7.50 (m, 2H), 7.43 (s, 1H), 7.42 – 7.37 (m, 1H), 7.17 (d, J = 4.9 Hz, 1H), 5.92 (d, J = 4.3, 2H), 3.08 (s, 3H) ppm.

^{13}C NMR (101 MHz, $CDCl_3$): δ = 163.9, 156.5, 154.7, 136.1, 135.9, 128.8, 128.0, 127.8, 125.1, 122.8, 119.8, 117.8, 117.3, 116.4, 113.6, 103.2, 42.6 ppm.

IR (ATR): v = 3235 (w), 2970 (w), 2929 (w), 2905 (vw), 2868 (vw), 2851 (vw), 2829 (w), 1697 (s), 1662 (vs), 1592 (vs), 1519 (w), 1476 (w), 1462 (w), 1445 (w), 1402 (vs), 1380 (vs), 1366 (vs), 1357 (vs), 1337 (s), 1312 (w), 1290 (w), 1255 (m), 1238 (s), 1225 (vs), 1193 (vs), 1160 (vs), 1118 (s), 1082 (m), 1054 (m), 1028 (s), 1003 (m), 962 (w), 949 (w), 941 (w), 921 (m), 904 (w), 891 (w), 863 (w), 829 (m), 812 (m), 769 (s), 756 (w), 745 (vs), 731 (s), 708 (m), 662 (w), 633 (w), 609 (w), 602 (w), 568 (m), 545 (s), 509 (m), 497 (m), 469 (w), 453 (w), 428 (w), 385 (w) cm^{-1}.

HRMS (FAB): *m*/*z* calcd. for $C_{18}H_{15}N_3O_2S$ [M], 337.0880; found, 337.0878.

UV/Vis (DMSO): λ_{max} = 504 nm.

(Z)-3-(thiophen-2-ylmethylene)-2,3-dihydropyrazino[1,2-a]indole-1,4-dione (**2**)

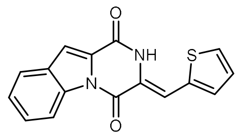

According to general procedure. Purified *via* recrystallization from MeCN to afford the pure product as a yellow solid with a yield of 60%.

^{1}H NMR (400 MHz, DMSO-d_6): δ = 9.94 (bs, 1H), 8.49 (d, J = 8.4 Hz, 1H), 7.87 – 7.83 (m, 2H), 7.71 (d, J = 3.9 Hz, 1H), 7.60 – 7.54 (m, 2H), 7.45 (t, J = 7.3 Hz, 2H), 7.47 – 7.43 (m, 1H), 7.33 (s, 1H) 7.25 – 7.23 (m, 1H) ppm.

^{13}C NMR (101 MHz, DMSO-d_6): not reported due to low solubility.

IR (ATR): ν = 3305 (w), 3119 (w), 3088 (w), 3077 (w), 3058 (w), 3020 (w), 2184 (w), 1677 (vs), 1606 (vs), 1564 (s), 1506 (w), 1443 (s), 1368 (vs), 1339 (vs), 1323 (vs), 1258 (vs), 1238 (vs), 1222 (vs), 1196 (vs), 1156 (s), 1140 (vs), 1118 (s), 1072 (s), 1051 (s), 1028 (vs), 1004 (s), 979 (m), 939 (m), 912 (m), 901 (w), 888 (s), 860 (s), 844 (m), 826 (s), 786 (w), 741 (vs), 724 (vs), 694 (s), 636 (m), 620 (s), 611 (m), 601 (s), 582 (m), 550 (vs), 518 (vs), 483 (m), 459 (s), 426 (m) cm^{-1}.

HRMS (EI): *m*/*z* calcd. for $C_{16}H_{11}N_2O_2S$: 295.0536 [M]; found: 295.0536.

UV/Vis (DMSO): λ_{max} = 388 nm.

(Z)-3-((1H-imidazol-5-yl)methylene)-2,3-dihydropyrazino[1,2-a]indole-1,4-dione (**3**)

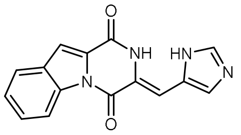

According to general procedure. Purified *via* preparative HPLC (gradient MeCN in H_2O, 0.1%TFA: 20% → 80%, flowrate: 15 mL/min, run time: 45 min) to afford the pure product as a bright yellow solid with a yield of 64%.

^{1}H NMR (400 MHz, DMSO-*d$_6$*): δ = 12.80 (s, 1H), 12.05 (s, 1H), 8.51 (d, *J* = 8.3 Hz, 1H), 8.08 (s, 1H), 7.86 (d, *J* = 7.9 Hz, 1H), 7.76 (s, 1H), 7.56 – 7.52 (m, 2H), 7.44 (t, *J* = 7.6 Hz, 1H), 7.08 (s, 1H) ppm.

^{13}C NMR (101 MHz, DMSO): δ = 156.9, 153.9, 141.5, 141.4, 136.7, 135.5, 129.8, 128.9, 127.5, 125.1, 123.2, 122.8, 116.8, 111.9, 49.1 ppm.

IR (ATR): ν = 3162 (w), 3123 (w), 3114 (w), 3097 (w), 3081 (w), 3051 (w), 3027 (w), 3014 (w), 1697 (w), 1673 (vs), 1612 (s), 1588 (s), 1572 (m), 1453 (w), 1445 (m), 1404 (vs), 1381 (vs), 1358 (vs), 1336 (vs), 1313 (s), 1296 (m), 1247 (m), 1221 (m), 1197 (s), 1184 (m), 1162 (w), 1145 (w), 1111 (w), 1099 (w), 1030 (s), 1006 (w), 997 (w), 925 (w), 884 (w), 854 (w), 813 (s), 762 (s), 747 (vs), 721 (s), 688 (vs), 662 (m), 616 (w), 595 (w), 579 (m), 543 (vs), 482 (s), 462 (s), 429 (m), 411 (w) cm^{-1}.

HRMS (EI): *m*/*z* calcd. for $C_{15}H_{11}N_4O_2$ [M], 279.0877; found, 279.0878.

UV/Vis (DMSO): λ_{max} = 385 nm.

(Z)-3-((1H-pyrrol-2-yl)methylene)-1-acetylpiperazine-2,5-dione (**4**)

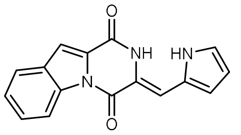

According to general procedure. Purified *via* recrystallization from MeCN to afford the pure product as a yellow-ocher solid with a yield of 66%.

^{1}H NMR (400 MHz, DMSO-*d$_6$*): δ = δ = 11.49 (s, 1H), 9.98 (s, 1H), 8.52 (d, *J* = 8.3 Hz, 1H), 7.84 (d, *J* = 7.8 Hz, 1H), 7.55 (ddd, *J* = 8.5, 7.2, 1.3 Hz, 1H), 7.49 (s, 1H), 7.43 (t, *J* = 7.2 Hz, 1H), 7.20 (s, 1H), 7.13 (d, *J* = 1.6 Hz, 0H), 6.97 (d, *J* = 3.8 Hz, 1H), 6.30 (t, *J* = 3.0 Hz, 1H) ppfm.

^{13}C NMR (101 MHz, DMSO-*d$_6$*): δ = 156.9, 154.6, 135.0, 129.0, 128.5, 127.1, 126.2, 124.8, 123.1, 122.8, 120.8, 116.3, 114.4, 111.7, 111.2, 111.0 ppm.

IR (ATR): *ν* = 3337 (w), 3305 (w), 3145 (w), 1666 (vs), 1647 (vs), 1605 (vs), 1595 (vs), 1582 (s), 1567 (m), 1443 (s), 1409 (m), 1392 (vs), 1375 (vs), 1351 (vs), 1333 (vs), 1264 (s), 1237 (w), 1228 (w), 1197 (s), 1145 (m), 1130 (vs), 1103 (m), 1068 (w), 1027 (s), 1004 (w), 878 (m), 816 (m), 800 (w), 734 (vs), 698 (s), 679 (s), 647 (m), 629 (w), 612 (w), 591 (s), 569 (s), 518 (w), 501 (w), 470 (w), 428 (w) cm^{-1}.

HRMS (EI): *m*/*z* calcd. for $C_{16}H_{11}N_3O_2$ [M], 277.0846; found, 277.0847.

UV/Vis (DMSO): λ_{max} = 418 nm.

(Z)-3-benzylidene-2,3-dihydropyrazino[1,2-a]indole-1,4-dione (**5**)

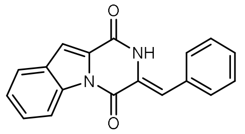

According to general procedure. Purified *via* recrystallization from toluene to afford the pure compound, which was isolated as a pale-yellow solid in 55% yield.

^{1}H NMR (400 MHz, DMSO-*d*$_6$): *δ* = 10.45 (s, 1H), 8.50 (d, *J* = 8.4 Hz, 1H), 7.86 (d, *J* = 7.9 Hz, 1H), 7.66 (d, *J* = 7.6 Hz, 2H), 7.58 (t, *J* = 7.8 Hz, 1H), 7.53 (s, 1H), 7.46 (m, 3H), 7.38 (t, *J* = 7.4 Hz, 1H), 7.16 (s, 1H) ppm.

^{13}C NMR (101 MHz, DMSO-*d*$_6$): 13C NMR (101 MHz, DMSO) δ 156.5, 154.8, 135.1, 133.0, 129.9, 128.8, 128.7, 128.6, 127.6, 127.1, 125.1, 123.0, 118.6, 116.3, 112.8 ppm.

IR (ATR): *ν* = 3162 (w), 3123 (w), 3114 (w), 3097 (w), 3081 (w), 3051 (w), 3027 (w), 3014 (w), 1697 (w), 1673 (vs), 1612 (s), 1588 (s), 1572 (m), 1453 (w), 1445 (m), 1404 (vs), 1381 (vs), 1358 (vs), 1336 (vs), 1313 (s), 1296 (m), 1247 (m), 1221 (m), 1197 (s), 1184 (m), 1162 (w), 1145 (w), 1111 (w), 1099 (w), 1030 (s), 1006 (w), 997 (w), 925 (w), 884 (w), 854 (w), 813 (s), 762 (s), 747 (vs), 721 (s), 688 (vs), 662 (m), 616 (w), 595 (w), 579 (m), 543 (vs), 482 (s), 462 (s), 429 (m), 411 (w) cm^{-1}.

HRMS (FAB): *m*/*z* calcd. for $C_{18}H_{12}N_2O_2$ [M], 288.0893; found, 288.0894.

UV/Vis (DMSO): λ_{max} = 363 nm.

(Z)-3-(4-(dimethylamino)benzylidene)-2,3-dihydropyrazino[1,2-a]indole-1,4-dione (**6**)

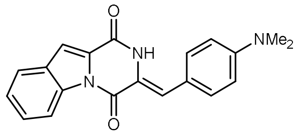

According to general procedure. Purified *via* recrystallization from toluene to afford the pure compound as a yellow solid in 70% yield.

^{1}H NMR (400 MHz, DMSO-*d*$_6$): *δ* = 10.18 (s, 1H), 8.52 (dd, *J* = 8.3, 0.9 Hz, 1H), 7.86 (d, *J* = 7.8 Hz, 1H), 7.57 (m, 3H), 7.51 (s, 1H), 7.47 – 7.42 (m, 1H), 7.14 (s, 1H), 6.79 (d, *J* = 9.0 Hz, 2H), 3.01 (s, 6H) ppm.

^{13}C NMR (101 MHz, $CDCl_3$): *δ* = 156.4, 154.8, 150.9, 136.0, 130.7, 128.7, 128.0, 127.8, 125.2, 123.0, 122.8, 121.6, 120.2, 117.2, 114.0, 112.5, 40.1 ppm.

IR (ATR): *ν* = 3225 (w), 3109 (w), 3054 (w), 2895 (w), 2854 (w), 2803 (w), 1694 (w), 1666 (vs), 1611 (m), 1584 (vs), 1524 (s), 1477 (w), 1442 (s), 1422 (w), 1401 (vs), 1363 (vs), 1334 (vs), 1261 (s), 1231 (s), 1213 (m), 1188 (vs), 1171 (vs), 1160 (s), 1143 (s), 1119 (m), 1065 (m), 1028 (vs), 1003 (m), 945 (m), 936 (m), 902 (w), 890 (m), 857 (w), 827 (m), 809 (vs), 769 (s), 756 (m), 744 (vs), 731 (vs), 705 (s), 632 (m), 609 (m), 577 (m), 544 (s), 521 (s), 507 (s), 496 (s), 477 (m), 469 (s), 428 (s), 418 (m) cm^{-1}.

HRMS (EI): *m*/*z* calcd. for $C_{20}H_{17}N_3O_2$ [M], 331.1315; found, 331.1317.

UV/Vis (DMSO): λ_{max} = 446 nm.

Tert-butyl(Z)-4-(4-((1,4-dioxo-1,2-dihydropyrazino[1,2-a]indol-3(4H)-ylidene)methyl)phenyl)piperazine-1-carboxylate (**7-Boc**)

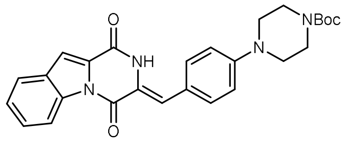

According to general procedure. Purified *via* flash gradient column chromatography CH_2Cl_2/EtOAc: (4/1 → 3/2) to afford the pure product as an orange solid with a yield of 62%.

^{1}H NMR (400 MHz, $CDCl_3$): δ = 8.64 (dt, *J* = 8.4, 0.9 Hz, 1H), 8.19 (s, 1H), 7.76 (d, *J* = 7.9 Hz, 1H), 7.61 – 7.52 (m, 2H), 7.47 – 7.38 (m, 3H), 7.30 (s, 1H), 6.96 (d, *J* = 8.9 Hz, 2H), 3.60 (t, *J* = 5.3 Hz, 4H), 3.28 (t, *J* = 5.3 Hz, 4H), 1.50 (s, 9H) ppm.

^{13}C NMR (101 MHz, $CDCl_3$): δ = 156.2, 154.9, 154.8, 151.5, 136.2, 130.6, 128.8, 128.3, 127.8, 125.5, 124.6, 123.5, 123.0, 120.6, 117.3, 116.0, 114.6, 80.3, 48.0, 28.6 ppm.

IR (ATR): *ν* = 3235 (w), 2970 (w), 2929 (w), 2905 (vw), 2868 (vw), 2851 (vw), 2829 (w), 1697 (s), 1662 (vs), 1592 (vs), 1519 (w), 1476 (w), 1462 (w), 1445 (w), 1402 (vs), 1380 (vs), 1366 (vs), 1357 (vs), 1337 (s), 1312 (w), 1290 (w), 1255 (m), 1238 (s), 1225 (vs), 1193 (vs), 1160 (vs), 1118 (s), 1082 (m), 1054 (m), 1028 (s), 1003 (m), 962 (w), 949 (w), 941 (w), 921 (m), 904 (w), 891 (w), 863 (w), 829 (m), 812 (m), 769 (s), 756 (w), 745 (vs), 731 (s), 708 (m), 662 (w), 633 (w), 609 (w), 602 (w), 568 (m), 545 (s), 509 (m), 497 (m), 469 (w), 453 (w), 428 (w), 385 (w) cm^{-1}.

HRMS (EI): *m*/*z* calcd. for $C_{27}H_{28}N_4O_4$ [M], 472.2105; found, 472.2104.

UV/Vis (DMSO): λ_{max} = 411 nm.

(Z)-3-(4-(piperazin-1-yl)benzylidene)-2,3-dihydropyrazino[1,2-a]indole-1,4-dione (**7**)

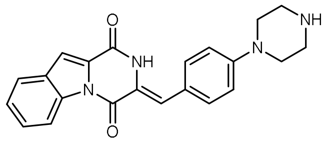

7-Boc (100 mg, 212 µmol, 1.00 equiv) was dissolved in 10 mL of a 20 vol% TFA (3.26 g, 2.20 mL, 28.6 mmol, 135 equiv) and 80% dry CH_2Cl_2 (8.80 mL), containing 1 vol%) TIPS (85.0 mg, 110 µL, 537 µmol, 2.54 equiv) at 0 °C (ice bath). Subsequently, the ice bath was removed and the solution was allowed to warm to ambient temperature. After 2.5 hours the starting material had been consumed (reaction control was performed by TLC) and toluene (50 mL) was added and the mixture was removed from all volatiles

by azeotropic distillation. The TFA salt of the target compound was isolated as a yellow solid in 89% yield. A part of the compound was washed with sat. aq. solution of NaHCO3 and extracted with ethyl acetate to obtain the neutralized compound.

^{1}H NMR (400 MHz, DMSO-*d*$_6$): δ = 8.52 (d, J = 8.3 Hz, 1H), 7.86 (d, J = 7.9 Hz, 1H), 7.57 (dd, J = 8.4, 5.4 Hz, 3H), 7.52 (s, 1H), 7.45 (t, J = 7.6 Hz, 1H), 7.12 (s, 1H), 7.00 (d, J = 9.0 Hz, 2H), 3.26 – 3.17 (m, 4H), 2.89 – 2.81 (m, 4H) ppm.

^{13}C NMR (101 MHz, DMSO-*d*$_6$): δ = 157.1, 155.2, 150.5, 135.6, 132.1, 129.2, 129.0, 128.0, 125.5, 124.7, 124.3, 123.4, 120.2, 116.8, 115.6, 113.0, 45.0, 43.0 ppm.

IR (ATR): *ν* = 3245 (w), 3054 (w), 2921 (w), 2850 (w), 1664 (vs), 1591 (vs), 1519 (s), 1469 (w), 1445 (s), 1400 (vs), 1381 (vs), 1341 (vs), 1273 (vs), 1256 (vs), 1239 (vs), 1224 (vs), 1193 (vs), 1142 (vs), 1098 (s), 1067 (m), 1048 (m), 1027 (vs), 1001 (s), 990 (s), 960 (m), 938 (m), 912 (s), 892 (m), 866 (m), 824 (s), 806 (vs), 768 (s), 745 (vs), 732 (vs), 720 (s), 710 (s), 662 (m), 649 (s), 636 (m), 623 (m), 609 (s), 602 (s), 567 (vs), 544 (vs), 523 (vs), 509 (vs), 496 (vs), 469 (s), 456 (s), 443 (m), 426 (vs), 416 (s), 401 (s), 387 (s) cm^{-1}.

HRMS (EI): *m*/*z* calcd. for $C_{22}H_{20}N_4O_2$ [M], 372.1581; found, 372.1579.

UV/Vis (DMSO): λ_{max} = 415 nm.

(Z)-3-((2,3,6,7-tetrahydro-1H,5H-pyrido[3,2,1-ij]quinolin-9-yl)methylene)-2,3-dihydropyrazino[1,2-a]indole-1,4-dione (**8**)

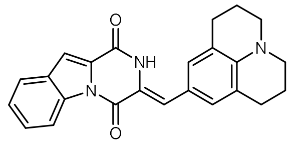

According to general procedure. The residual solid was washed with Et_2O and the target compound was isolated as a red solid in 53% yield.

^{1}H NMR (400 MHz, $CDCl_3$): δ = 8.66 (dd, J = 8.4, 1.0 Hz, 1H), 8.25 (s, 1H), 7.76 (dt, J = 7.9, 1.1 Hz, 1H), 7.59 – 7.52 (m, 1H), 7.52 (s, 1H), 7.41 (ddd, J = 8.3, 7.3, 1.1 Hz, 1H), 7.23 (s, 1H), 6.97 (s, 2H), 3.26 (dd, J = 6.7, 4.8 Hz, 4H), 2.77 (t, J = 6.4 Hz, 4H), 2.04 – 1.93 (m, 4H) ppm.

^{13}C NMR (101 MHz, $CDCl_3$): δ = 156.6, 154.9, 144.3, 136.1, 128.8, 128.5, 128.0, 127.9, 125.2, 122.8, 122.6, 122.1, 121.9, 119.4, 117.3, 113.8, 50.0, 27.9, 21.6 ppm.

IR (ATR): *ν* = 3116 (s), 3020 (s), 3007 (s), 2936 (s), 2928 (s), 2849 (s), 2805 (s), 2660 (w), 2514 (w), 1756 (w), 1742 (w), 1662 (m), 1588 (m), 1520 (w), 1442 (m), 1390 (vs), 1383 (vs), 1336 (vs), 1310 (vs), 1279 (s), 1242 (s), 1208 (s), 1173 (s), 1160 (s), 1145 (s), 1103 (m), 1077 (m), 1051 (w), 1028 (m), 1006 (w), 983 (w), 935 (w), 904 (w), 860 (w), 820 (w), 789 (w), 745 (m), 735 (m), 703 (w), 635 (w), 612 (w) cm^{-1}.

HRMS (FAB): *m*/*z* calcd. for $C_{24}H_{21}N_3O_2$ [M], 383.1628; found, 383.1630.

UV/Vis (DMSO): λ_{max} = 481 nm.

(Z)-3-((5-(tert-butyl)-1H-imidazol-4-yl)methylene)-2,3-dihydropyrrolo[1,2-a]pyrazine-1,4-dione **(9, PyrPlin)**

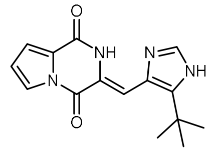

Synthesized according to general procedure and purified *via* recrystallization from MeCN. The target compound was isolated as a yellow solid in a yield of 54%.

^{1}H NMR (400 MHz, DMSO-d$_6$): δ = 12.52 (s, 1H), 12.09 (s, 1H), 7.94 (s, 1H), 7.74 (dd, J = 3.1, 1.5 Hz, 1H), 7.26 (s, 1H), 7.08 (dd, J = 3.5, 1.5 Hz, 1H), 6.64 (t, J = 3.3 Hz, 1H), 1.42 (s, 9H) ppm.

^{13}C NMR (101 MHz, DMSO-d$_6$): δ = 155.1, 152.8, 143.0, 135.2, 130.9, 125.0, 123.4, 120.5, 117.1, 114.9, 110.5, 32.2, 30.7 ppm.

IR (ATR): ν = 3184 (m), 3129 (w), 3085 (w), 3046 (w), 3034 (w), 3002 (w), 2990 (w), 2973 (m), 2955 (m), 2917 (m), 2868 (w), 2849 (m), 1691 (m), 1660 (vs), 1601 (s), 1570 (s), 1504 (m), 1451 (s), 1412 (vs), 1363 (m), 1351 (s), 1334 (vs), 1283 (s), 1266 (s), 1230 (m), 1210 (s), 1187 (s), 1111 (s), 1064 (s), 1017 (m), 1000 (s), 945 (m), 933 (s), 891 (m), 880 (m), 868 (w), 847 (s), 827 (m), 805 (vs), 738 (vs), 721 (vs), 700 (s), 656 (s), 643 (s), 594 (m), 581 (m), 538 (m), 516 (w), 501 (m), 480 (w), 466 (m), 458 (m), 443 (m), 388 (vs) cm^{-1}.

HRMS (FAB): *m*/*z* calcd. for $C_{15}H_{17}N_4O_2$ [M], 285.1346; found, 285.1347.

UV/Vis (DMSO): λ_{max} = 397 nm.

E-Isomer:

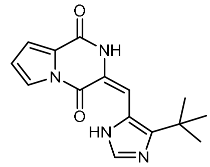

To afford the E-Isomer, a solution of **9** in DMSO was irradiated with 410 nm LEDs for 1 h and subsequently subjected to preparative HPLC (gradient MeCN in H_2O, 0.1%TFA: 20% → 80%, flowrate: 15 mL/min, run time: 45 min)

^{1}H NMR (400 MHz, DMSO-*d*$_6$): δ =12.60 (s, 1H), 10.86 (s, 1H), 7.79 – 7.71 (m, 2H), 7.44 (s, 1H), 7.04 (d, J = 5.1 Hz, 1H), 6.69 (t, J = 3.3 Hz, 1H), 1.39 (s, 9H) ppm.

^{13}C NMR (101 MHz, DMSO-*d*$_6$): δ = 157.4, 155.6, 153.3, 135.8, 125.1, 120.1, 119.4, 116.0, 115.4, 33.5, 31.4 ppm.

(Z)-3-((2,3,6,7-tetrahydro-1H,5H-pyrido[3,2,1-ij]quinolin-9-yl)methylene)-2,3-dihydropyrrolo[1,2-a]pyrazine-1,4-dione (**10**)

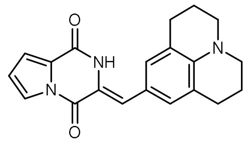

Synthesized according to general procedure and purified *via* recrystallization from MeCN. The target compound was isolated as red crystals in a yield of 24%.

^{1}H NMR (400 MHz, CDCl$_3$): δ = 8.04 (s, 1H), 7.72 (dd, J = 3.1, 1.6 Hz, 1H), 7.23 – 7.16 (m, 2H), 6.97 (s, 2H), 6.56 (t, J = 3.3 Hz, 1H), 3.32 – 3.22 (m, 4H), 2.76 (t, J = 6.4 Hz, 4H), 2.04 – 1.92 (m, 4H) ppm.

^{13}C NMR (101 MHz, CDCl$_3$): δ = 155.4, 154.3, 144.6, 128.9, 124.6, 124.1, 121.8, 121.4, 121.2, 119.3, 118.2, 115.0, 50.0, 27.9, 21.5 ppm.

IR (ATR): ν = 3220 (w), 3213 (w), 3126 (w), 2921 (m), 2849 (m), 2837 (m), 2808 (w), 1686 (w), 1639 (vs), 1613 (m), 1571 (vs), 1513 (vs), 1456 (m), 1449 (m), 1432 (m), 1404 (vs), 1367 (m), 1336 (vs), 1302 (vs), 1266 (vs), 1220 (vs), 1204 (vs), 1173 (vs), 1116 (vs), 1079 (vs), 1062 (vs), 1027 (s), 1006 (s), 965 (s), 926 (s), 914 (m), 901 (s), 880 (s), 858 (s), 853 (s), 792 (m), 769 (s), 755 (vs), 745 (vs), 735 (vs), 676 (s), 649 (s), 619 (s), 595 (s), 585 (s), 561 (m), 544 (m), 518 (s), 509 (s), 484 (s), 446 (vs), 421 (m), 411 (m), 391 (vs) cm^{-1}.

HRMS (FAB): *m*/*z* calcd. for $C_{24}H_{21}N_3O_2$ [M], 333.1472; found, 333.1469.

UV/Vis (DMSO): λ_{max} = 483 nm.

(Z)-3-((5-(tert-butyl)-1H-imidazol-4-yl)methylene)-2,3-dihydropyrrolo[1,2-a]pyrazine-1,4-dione (**11, IndPlin**)

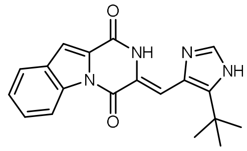

Synthesized according to procedure described in [2] and purified *via* recrystallization from MeCN. The target compound was isolated as yellow crystals in a yield of 54%. Analytical data in accordance with reference data.

Irradiation Intensities of LEDs

Table S1: *The LEDs applied to irradiate the photoswitchable compounds were characterized using the PowerMax USB (type PS19Q) sensor device (Coherent®), by placing them within a distance from the sensor, equivalent to the distance between sample and light source in the illumination experiments.*

λ_{max} of the LED diode (nm)	Max Power (mW/cm^2)	Max mean power measured (W)	Min Power (mW/cm^2)	Min mean power measured (W)
365	-	-	5,61E-01	1,59E-03
410	1,89E+01	5,36E-02	1,29E+01	3,65E-02
430	1,85E+01	5,23E-02	1,19E+01	3,38E-02
450	1,13E+00	3,21E-03	8,86E-01	2,51E-03
470	1,69E+01	4,80E-02	1,59E+01	4,52E-02
490	7,64E+00	2,17E-02	4,98E+00	1,41E-02
523	7,08E+00	2,01E-02	6,56E+00	1,86E-02
590	1,07E+01	3,03E-02	4,59E+00	1,30E-02
620	3,59E+01	1,02E-01	1,52E+01	4,32E-02

Absorption and Emission Spectra of the Photostationary States

All compounds **1-11** were each dissolved in DMSO with a resulting final concentration of 40 µM and 10.0 equiv. of $AscH_2$ (d = 5 mm). The absorption, as well as the fluorescence spectra of the non-irradiated samples were measured first. The samples were then irradiated with the denoted wavelengths in succession for defined time intervals (10 s - 10 min), until the PSS was reached (10 s - 60 min) and their spectra were measured. To record relative fluorescence intensities between the PSSs of one specific compound, all settings were kept constant in between measurements for this particular compound.

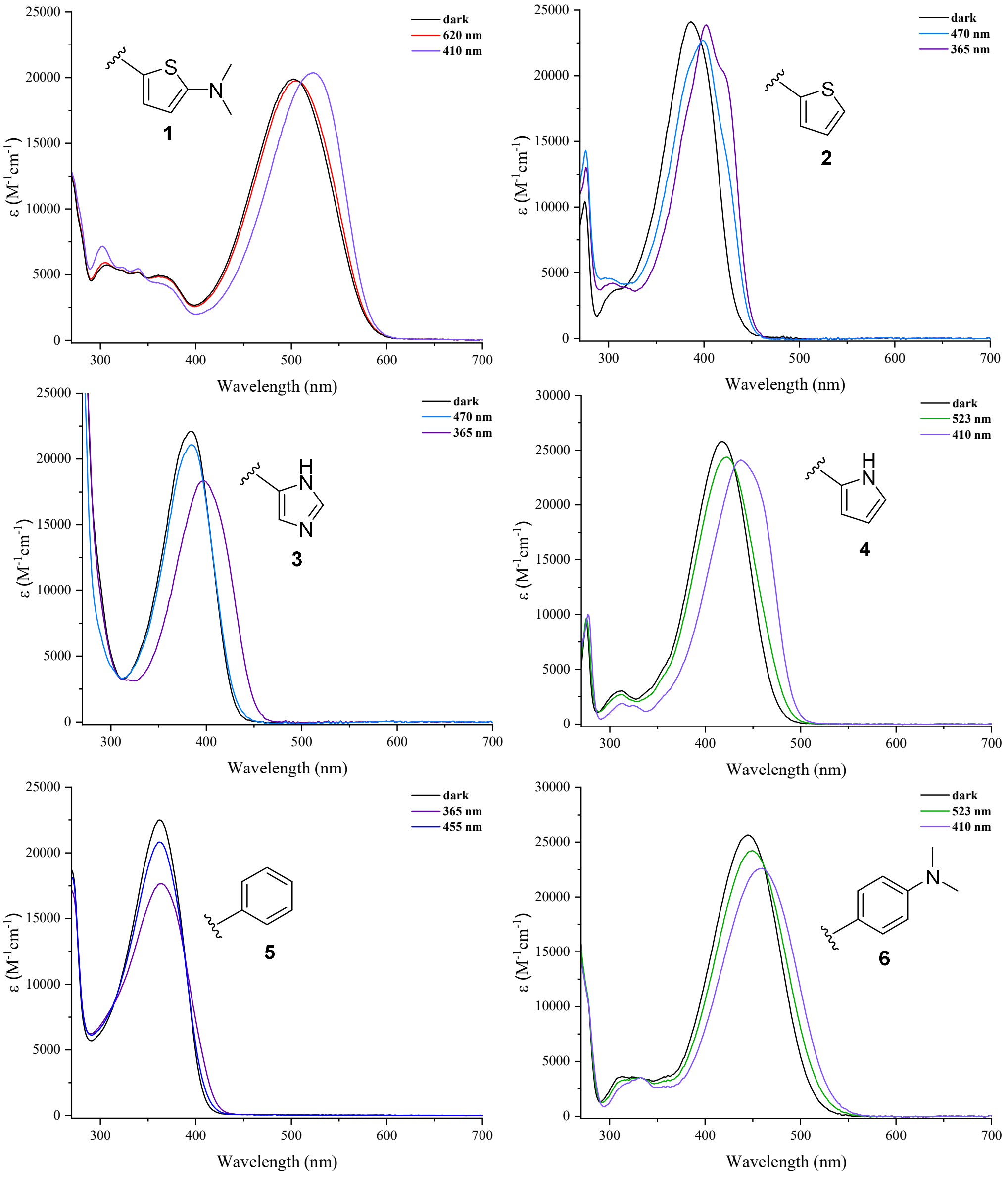

***Figure S1**: Spectra of the photostationary states established with the denoted illumination wavelengths of the Indolo-HPIs **1-6** as 40 µM solutions in DMSO, containing 10 eq. of $AscH_2$. The samples were irradiated in succession, starting from the shortest to longest wavelengths.*

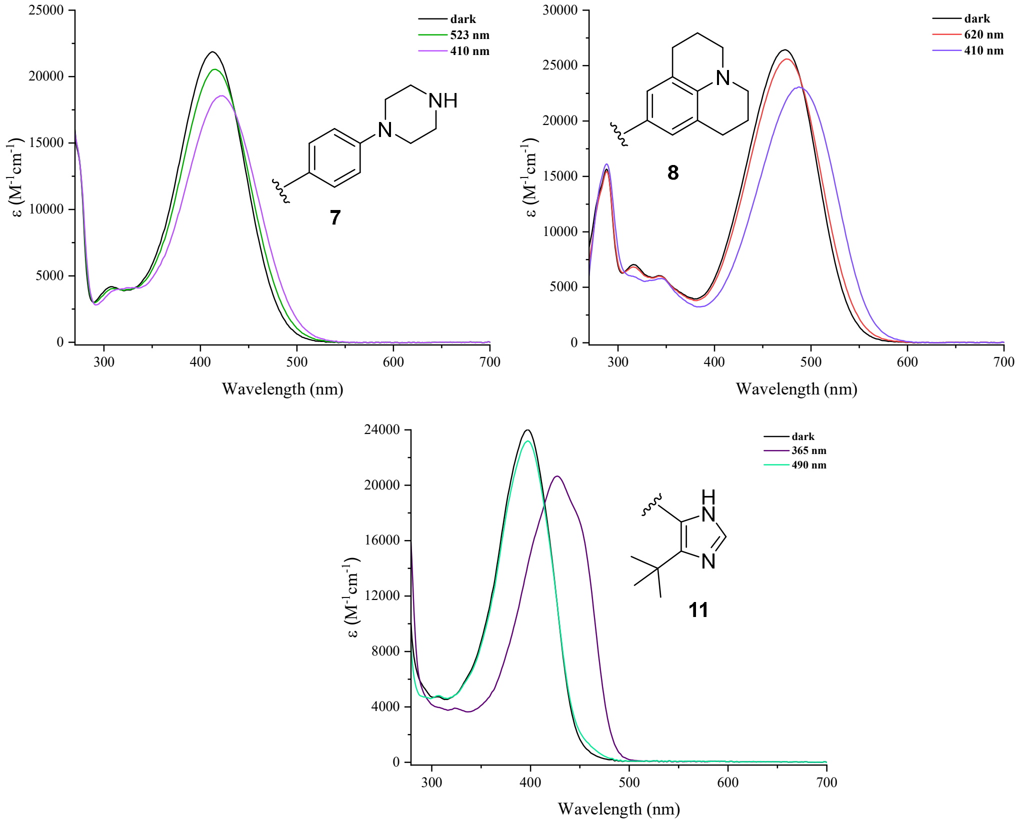

***Figure S2**: Spectra of the photostationary states established with the denoted illumination wavelengths of the Indolo-HPIs **7**, **8** and **11** as 40 µM solutions in DMSO, containing 10 eq. of AscH$_2$. The samples were irradiated in succession, starting from the shortest to longest wavelengths.*

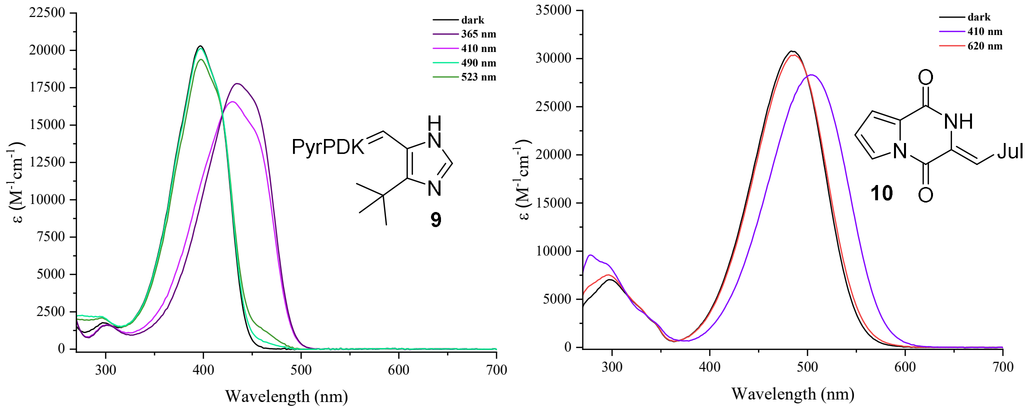

***Figure S3**: Spectra of the photostationary states established with the denoted illumination wavelengths of the Pyrrolo-HPIs **9-10** as 40 µM solutions in DMSO, containing 10 eq. of AscH$_2$. The samples were irradiated in succession, starting from the shortest to longest wavelengths.*

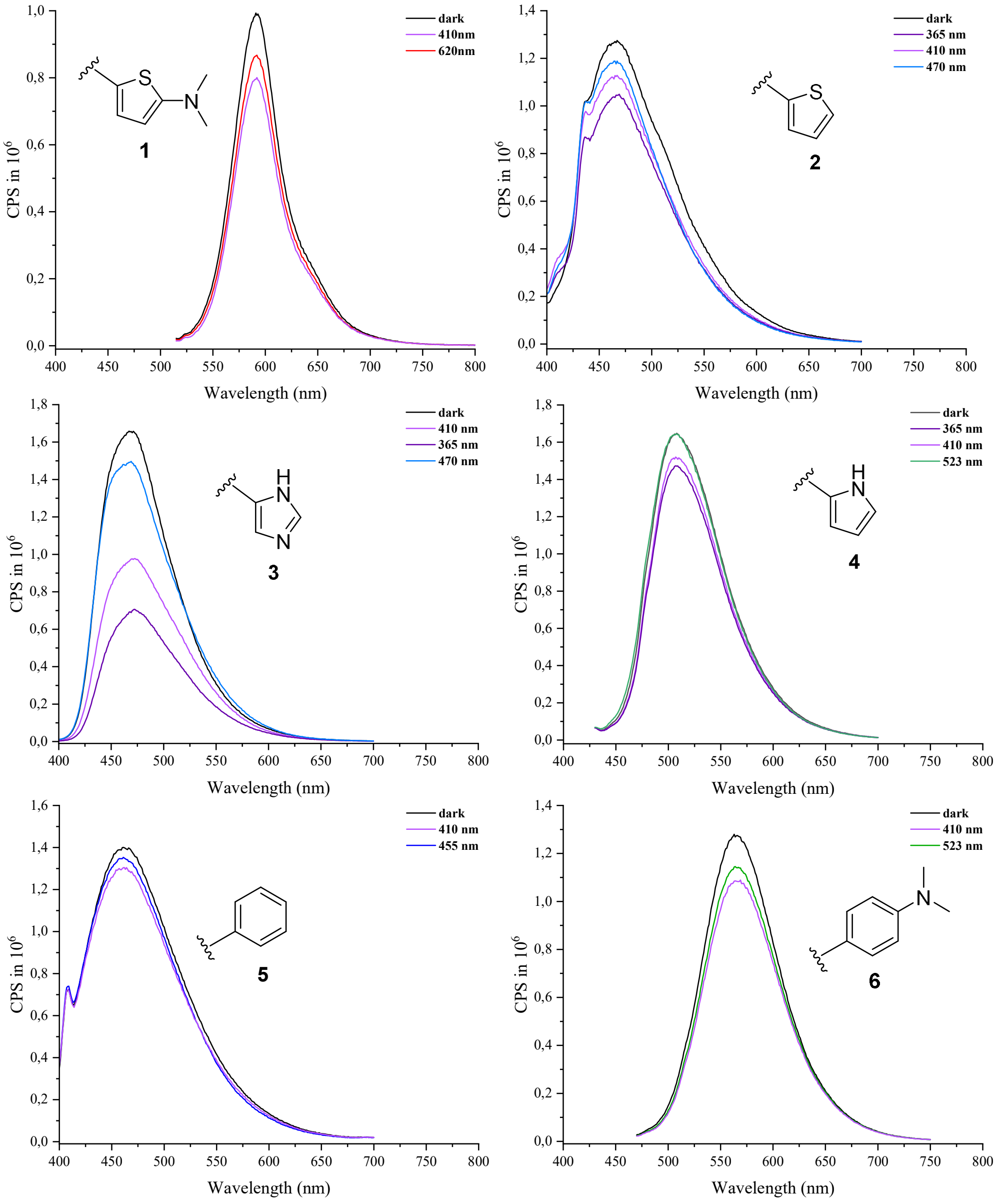

Figure S4: *Emission spectra of the photostationary states established with the denoted illumination wavelengths of IndHPIs* ***1-6*** *as 40 µM solutions in DMSO, containing 10 eq. of* $AscH_2$. *The samples were irradiated in succession, starting from the shortest to longest wavelengths. The spectra were recorded with constant excitation wavelengths and slit widths for each compound.*

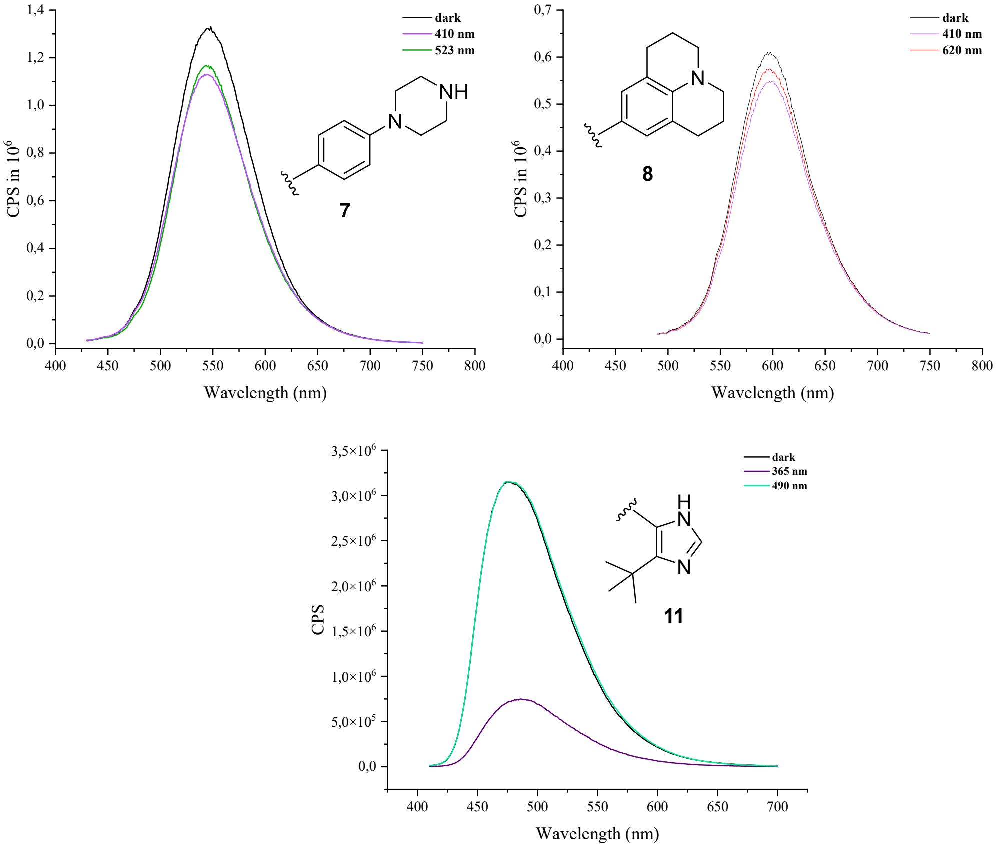

Figure S5: *Emission spectra of the photostationary states established with the denoted illumination wavelengths of IndHPIs **7**, **8** and **11** as 40 µM solutions in DMSO, containing 10 eq. of* $AscH_2$*. The samples were irradiated in succession, starting from the shortest to longest wavelengths. The spectra were recorded with constant excitation wavelengths and slit widths for each compound.*

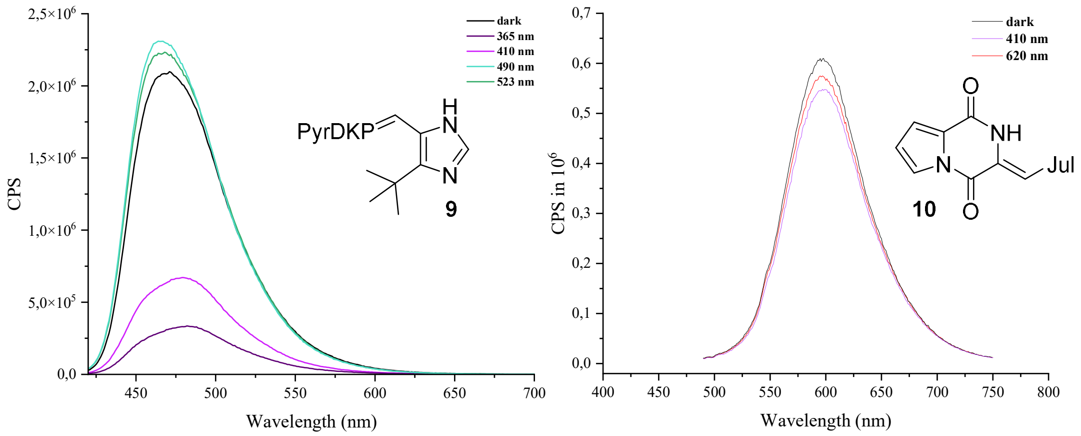

Figure S6: *Emission spectra of the photostationary states established with the denoted illumination wavelengths of PyrHPIs **9** and **10** as 40 µM solutions in DMSO, containing 10 eq. of* $AscH_2$*. The samples were irradiated in succession, starting from the shortest to longest wavelengths. The spectra were recorded with constant excitation wavelengths and slit widths for each compound.*

Photostability

Compound **1**, **4** and **8** were dissolved in DMSO to obtain two solutions for each compound with a final concentration of 40 µM, with or without 10.0 equiv. of ascorbic acid ($AscH_2$).

The solutions were both irradiated either with light of 410 nm (Fig. S7 – S9) or 365 nm wavelength (Fig. S10 – S11) in the indicated intervals for a total of up to 30 min. After every interval, a spectrum was recorded, and the resulting absorption maximum was tracked over the whole illumination period.

Plotting $1/\varepsilon_{max}d$ *vs.* t and a linear regression reveale the rate constant k (Ms^{-1}) and a direct linear relation between the decrease of absorption maximum and time.

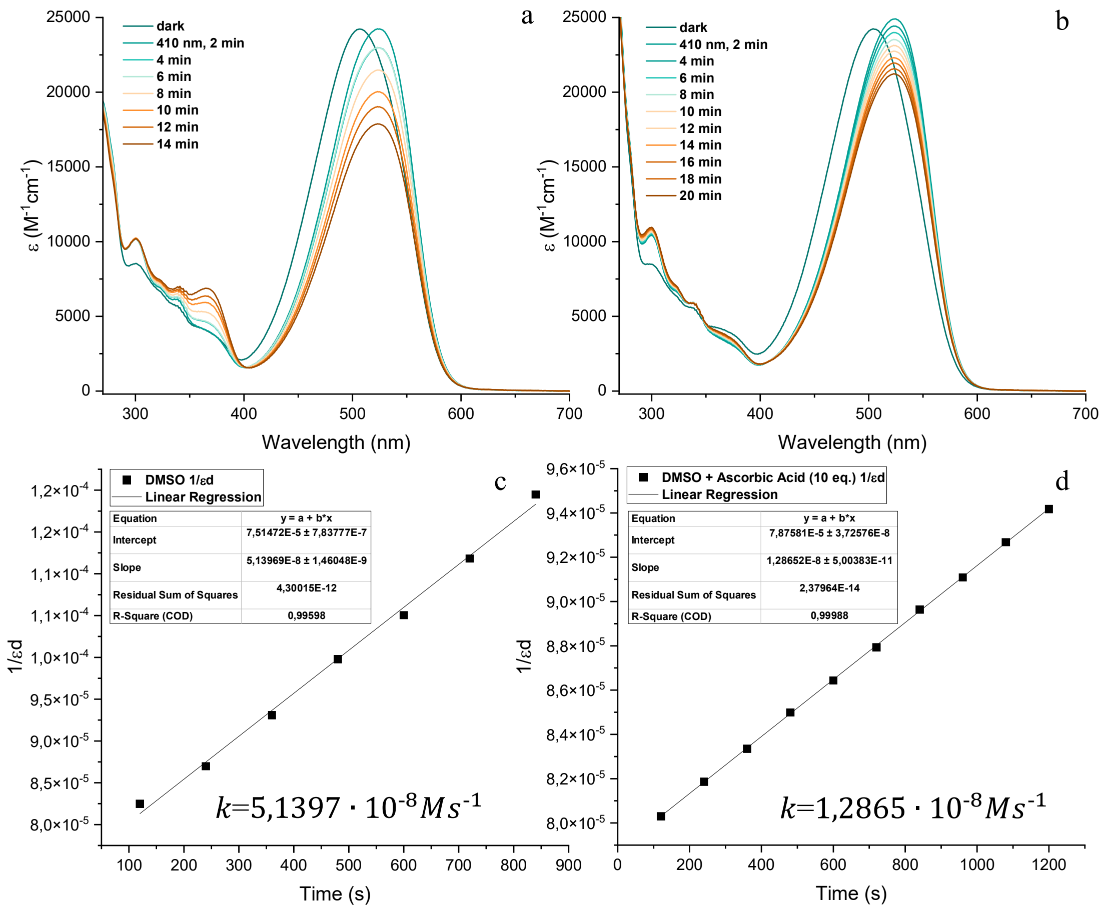

***Figure S7**: a) Absorption spectra of the PSS and continuous degradation of **1** (40 µM in DMSO), followed via decrease in absorption maximum upon continuous illumination with 410 nm. b) Absorption spectra of the PSS and continuous degradation of **1** (40 µM in DMSO) in the presence of excess AscH₂ (400 µM), followed via decrease in absorption maximum upon continuous illumination with 410 nm. c) Linear regression of 1/εd vs. time, using the maximal extinction coefficient ε_{max} of the mixture from Fig. S7a. d) Linear regression of 1/εd vs. time, using the maximal extinction coefficient ε_{max} of the mixture from Fig. S7b.*

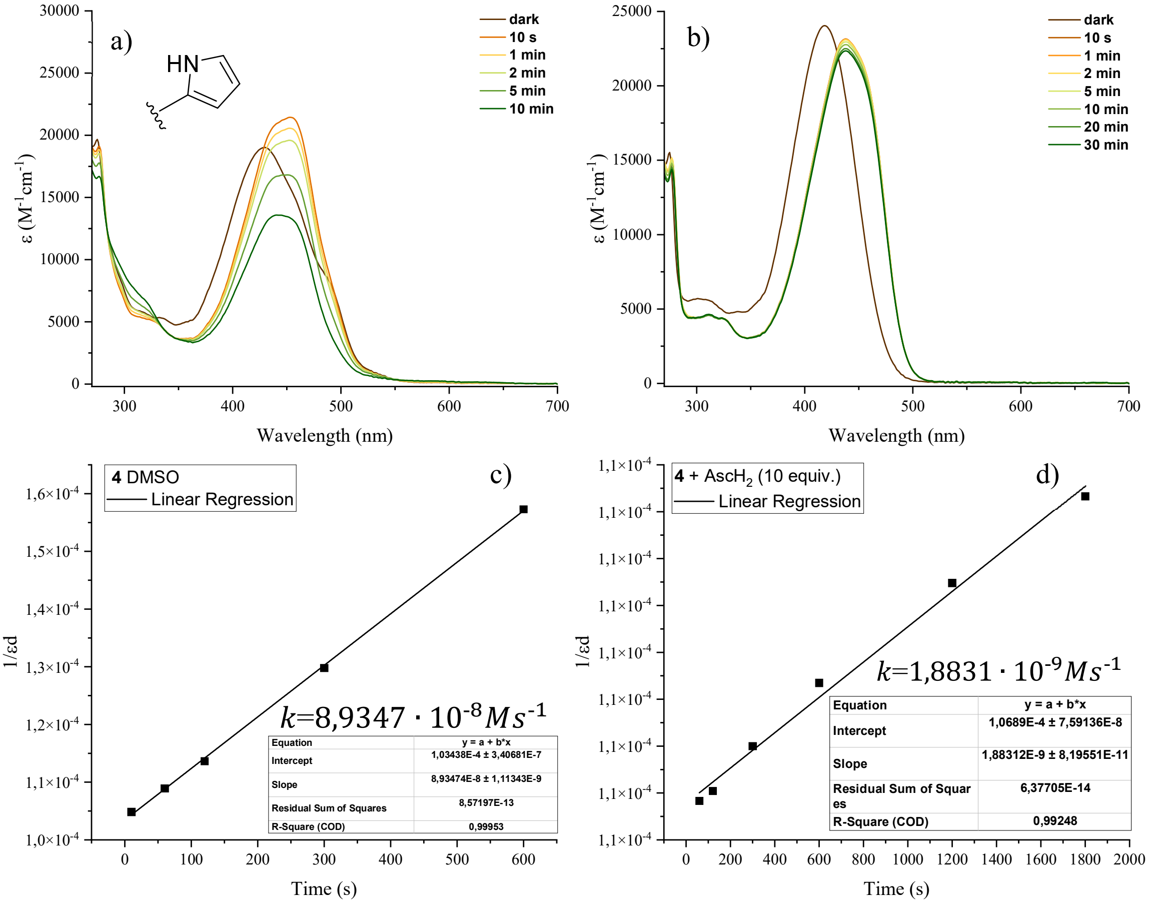

***Figure S8**: a) Absorption spectra of the PSS and continuous degradation of **4** (40 µM in DMSO), followed via decrease in absorption maximum upon continuous illumination with 410 nm. b) Absorption spectra of the PSS and continuous degradation of **4** (40 µM in DMSO) in the presence of excess $AscH_2$ (400 µM), followed via decrease in absorption maximum upon continuous illumination with 410 nm. c) Linear regression of 1/εd vs. time, using the maximal extinction coefficient ε_{max} of the mixture from Fig. S8a. d) Linear regression of 1/εd vs. time, using the maximal extinction coefficient ε_{max} of the mixture from Fig. S8b.*

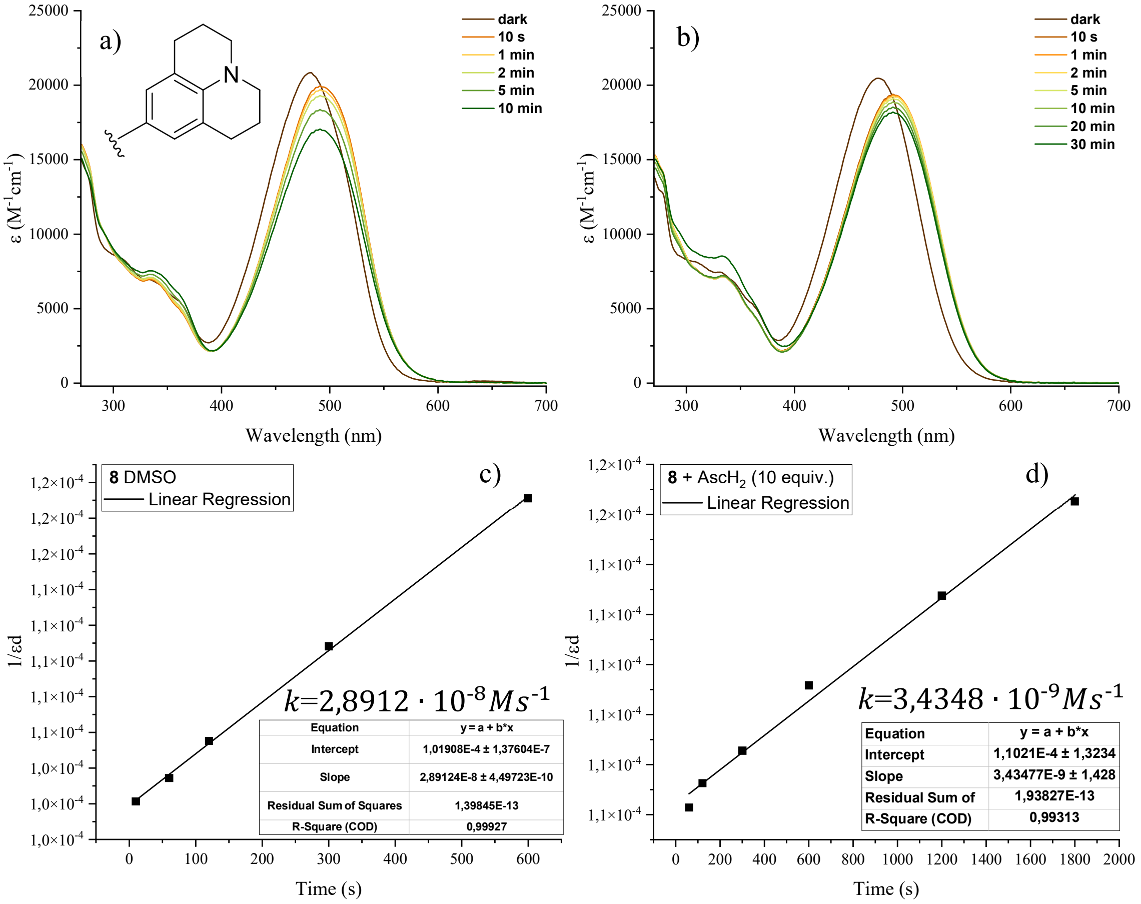

Figure S9: *a) Absorption spectra of the PSS and continuous degradation of* ***8*** *(40 µM in DMSO), followed via decrease in absorption maximum upon continuous illumination with 410 nm. b) Absorption spectra of the PSS and continuous degradation of* ***8*** *(40 µM in DMSO) in the presence of excess* $AscH_2$ *(400 µM), followed via decrease in absorption maximum upon continuous illumination with 410 nm. c) Linear regression of 1/εd vs. time, using the maximal extinction coefficient* ε_{max} *of the mixture from Fig. S9a. d) Linear regression of 1/εd vs. time, using the maximal extinction coefficient* ε_{max} *of the mixture from Fig. S9b.*

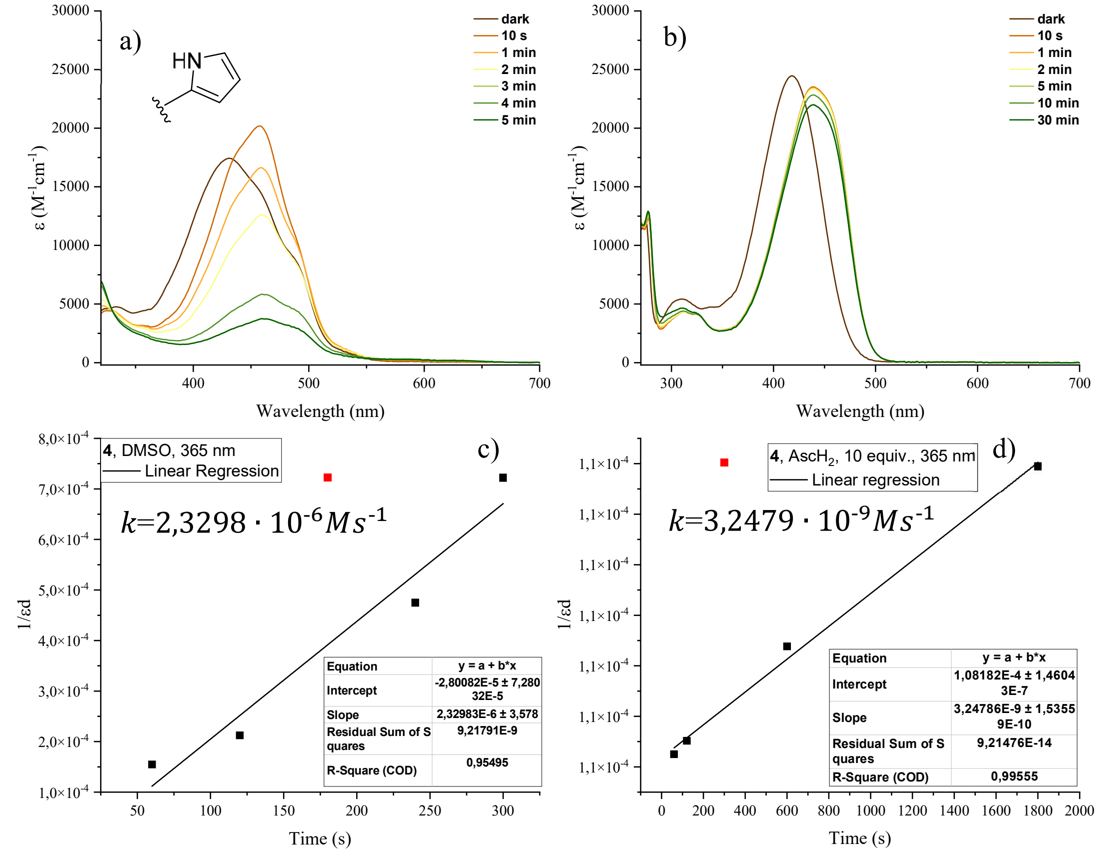

***Figure S10**: a) Absorption spectra of the PSS and continuous degradation of **4** (40 µM in DMSO), followed via decrease in absorption maximum upon continuous illumination with 365 nm. b) Absorption spectra of the PSS and continuous degradation of **4** (40 µM in DMSO) in the presence of excess $AscH_2$ (400 µM), followed via decrease in absorption maximum upon continuous illumination with 365 nm. c) Linear regression of 1/εd vs. time, using the maximal extinction coefficient ε_{max} of the mixture from Fig. S10a. d) Linear regression of 1/εd vs. time, using the maximal extinction coefficient ε_{max} of the mixture from Fig. S10b.*

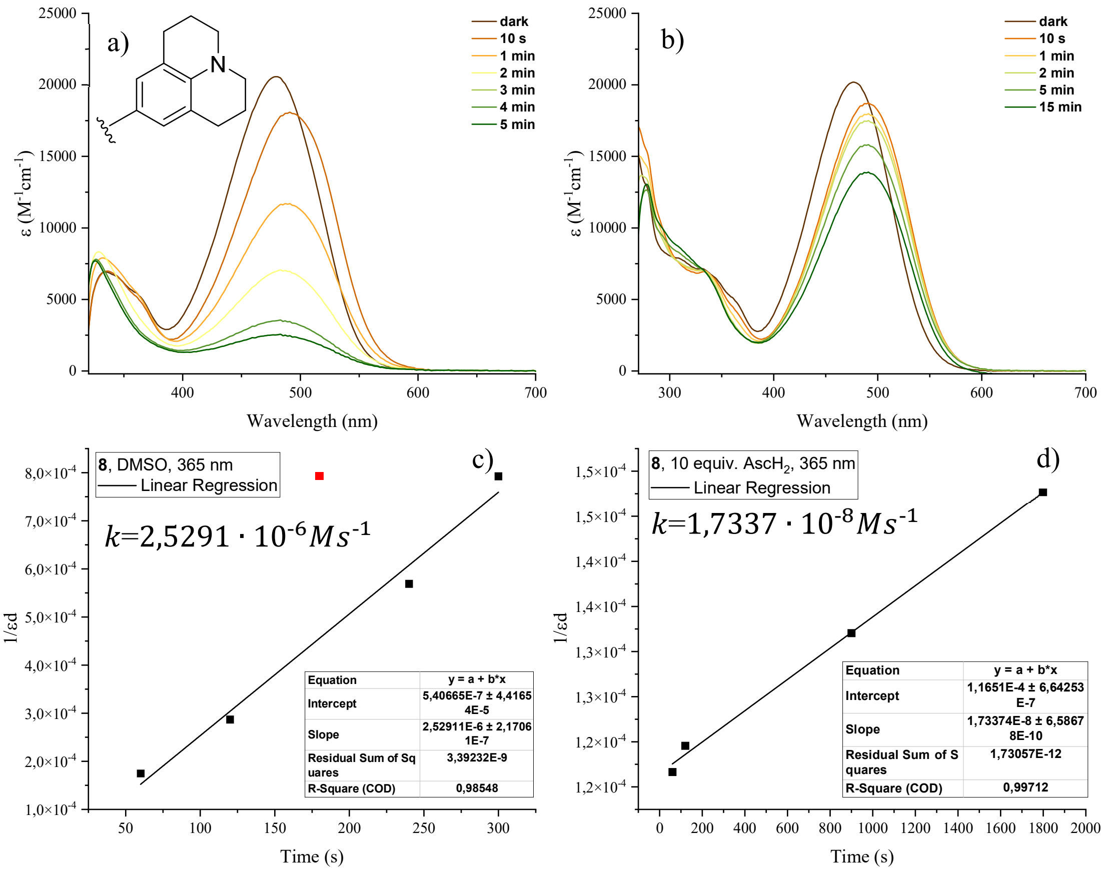

***Figure S11**: a) Absorption spectra of the PSS and continuous degradation of **8** (40 µM in DMSO), followed via decrease in absorption maximum upon continuous illumination with 365 nm. b) Absorption spectra of the PSS and continuous degradation of **8** (40 µM in DMSO) in the presence of excess $AscH_2$ (400 µM), followed via decrease in absorption maximum upon continuous illumination with 365 nm. c) Linear regression of 1/εd vs. time, using the maximal extinction coefficient ε_{max} of the mixture from Fig. S11a. d) Linear regression of 1/εd vs. time, using the maximal extinction coefficient ε_{max} of the mixture from Fig. S11b.*

To highlight the photostability of compound **1** under strongly reductive conditions and over multiple switching cycles, a 40 µM solution of **1**, containing 400 µM of ascorbic acid, was irradiated with 410 nm (20 s) and 590 nm (4 min) in succession for 10 switching cycles.

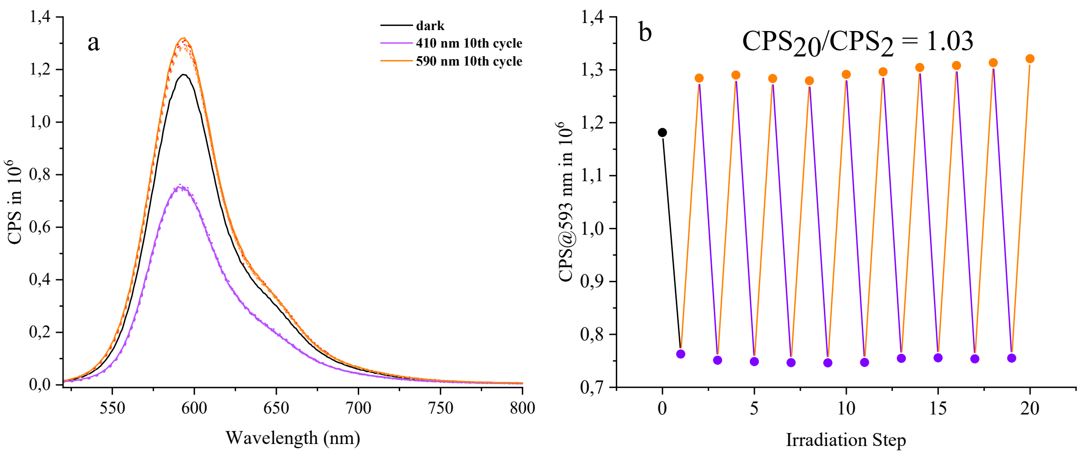

Figure S12: ***a*** *Fluorescence spectra of compound* ***1*** *in the non-irradiated and the photostationary states after 410 nm and 590 nm over 10 consecutive switching cycles (conc = 40 µM, 10.0 equiv. of* $AscH_2$ *in DMSO). Dashed lines represent cycles 1-9, straight lines represent the spectra of the last switching cycle.* ***b*** *Emission maximum tracked for all spectra shown in fig. S12a.*

Additionally, compound **9** was dissolved in DMSO to a concentration of 40 µM with an additional 10.0 equiv. of $AscH_2$ and illuminated in an alternating fashion with 365 nm and 490 nm for 10 consecutive cycles and the absorption maximum of ***Z*-9** was tracked (Fig. S13).

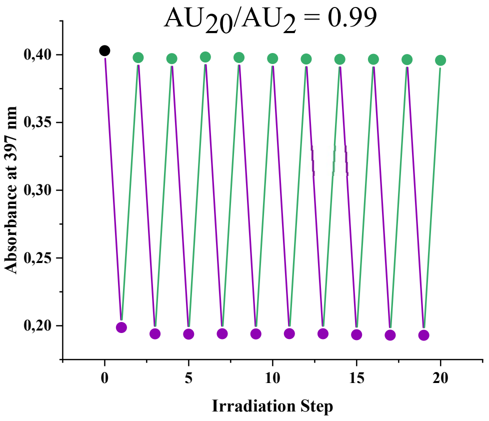

Figure S13: *Absorbance maximum of* ***Z-9*** *tracked over 10 consecutive switching cycles (conc = 40 µM, with 10.0 equiv. of* $AscH_2$ *in DMSO).*

Quantification of Photostationary States

To quantitatively analyze the PSS composition, ^{1}H-NMR samples of compounds **1-10**, dissolved in DMSO-d$_6$, were irradiated with the denoted wavelengths in succession (shortest to longest; 365 nm, 410 nm, 430 nm, 455 nm, 470 nm, 490 nm, 523 nm, 590 nm and 620 nm). The highlighted signals were integrated, compared to a reference signal, and the mean value of the integrals that belong to one species was calculated to determine the PSS.

Compounds **2**, **3**, **5**, **9** and **11** were dissolved in DMSO to a final concentration of 500 µM, also containing 10 eq. of AscH$_2$. Each solution subjected and HPLC analysis, afterwards illuminated with 365 nm and then split in several parts. These were then each irradiated with one of the indicated wavelengths of light for various time-intervals (ranging from 5 - 30 min) and subjected to HPLC analysis. The integral of the relevant signals was compared to the integral before illumination and the ratio of I_{PSS}/I_{dark} was calculated to determine the constitution of the PSS. All measurements were repeated with twice the duration of illumination to confirm that the isomeric ratio was at equilibrium (Fig. S16-S18).

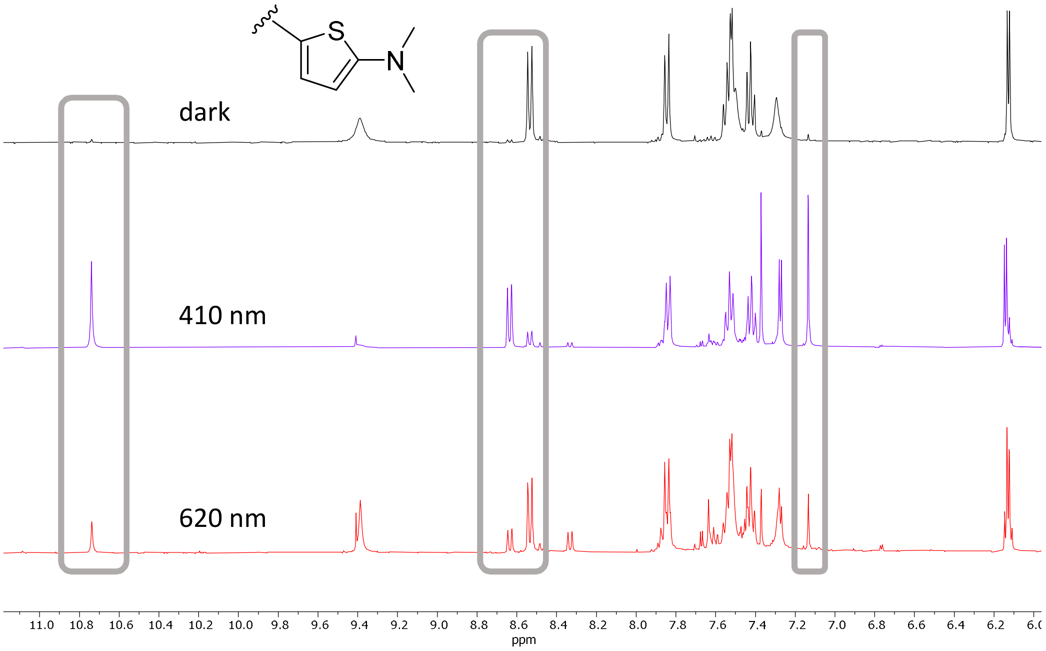

λ_{irrad}	410 nm	430 nm	455 nm	470 nm	490 nm	523 nm	590 nm	620 nm
**%*E***	74	71	66	65	56	38	26	22

Figure S14: *^{1}H-NMR of **1** in DMSO-d6 prior to irradiation and after illumination to equilibrium with the indicated wavelengths (top). Concentration of **E-1** in equilibrium under illumination with the indicated wavelengths (bottom).*

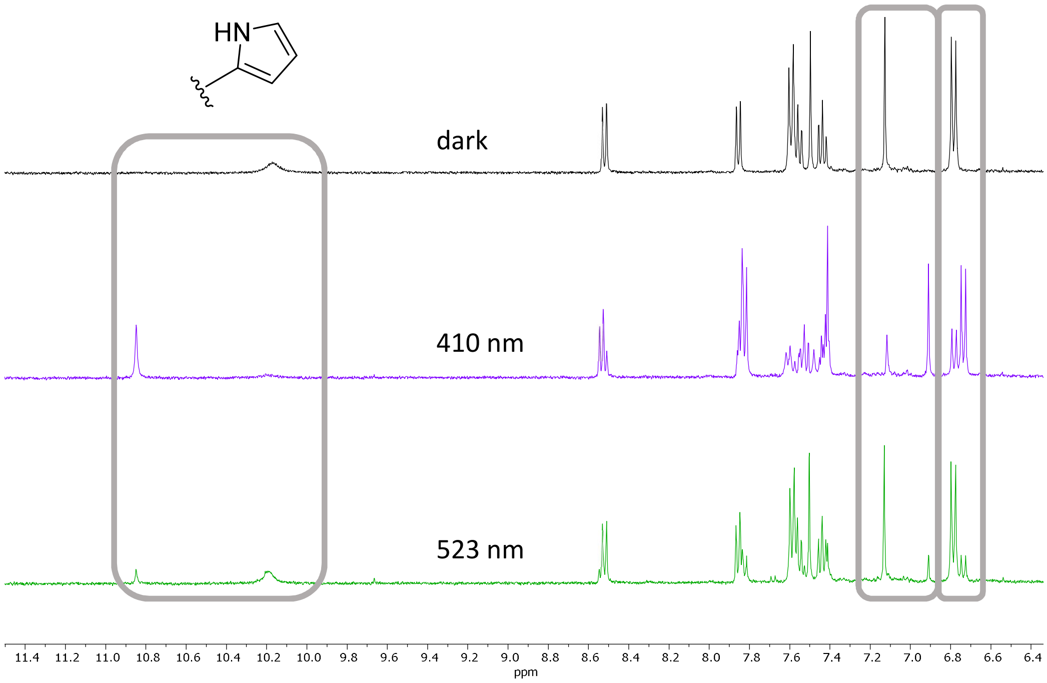

λ_{irrad}	410 nm	430 nm	455 nm	470 nm	490 nm	523 nm
**%*E***	73	69	51	39	30	15

Figure S14: *^{1}H-NMR of **4** in DMSO-d6 prior to irradiation and after illumination to equilibrium with the indicated wavelengths (top). Concentration of **E-4** in equilibrium under illumination with the indicated wavelengths (bottom).*

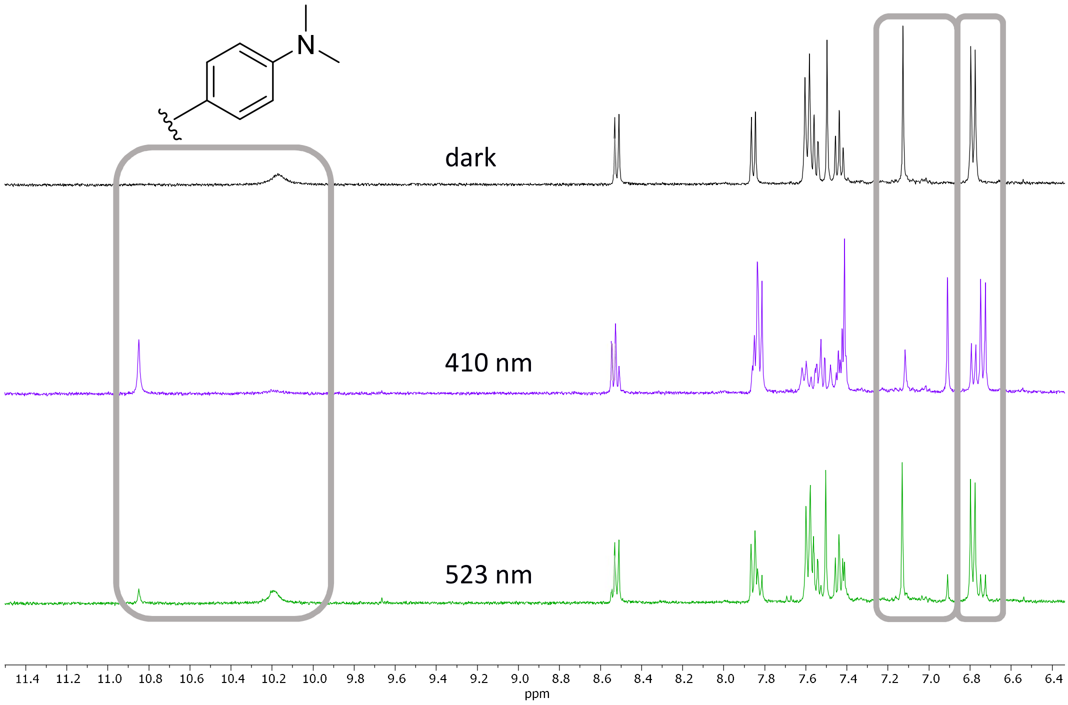

λ_{irrad}	410 nm	430 nm	455 nm	470 nm	490 nm	523 nm
**%*E***	62	60	55	49	29	23

Figure S15: ^{1}H-NMR of **6** in DMSO-d6 prior to irradiation and after illumination to equilibrium with the indicated wavelengths (top). Concentration of ***E*-6** in equilibrium under illumination with the indicated wavelengths (bottom).

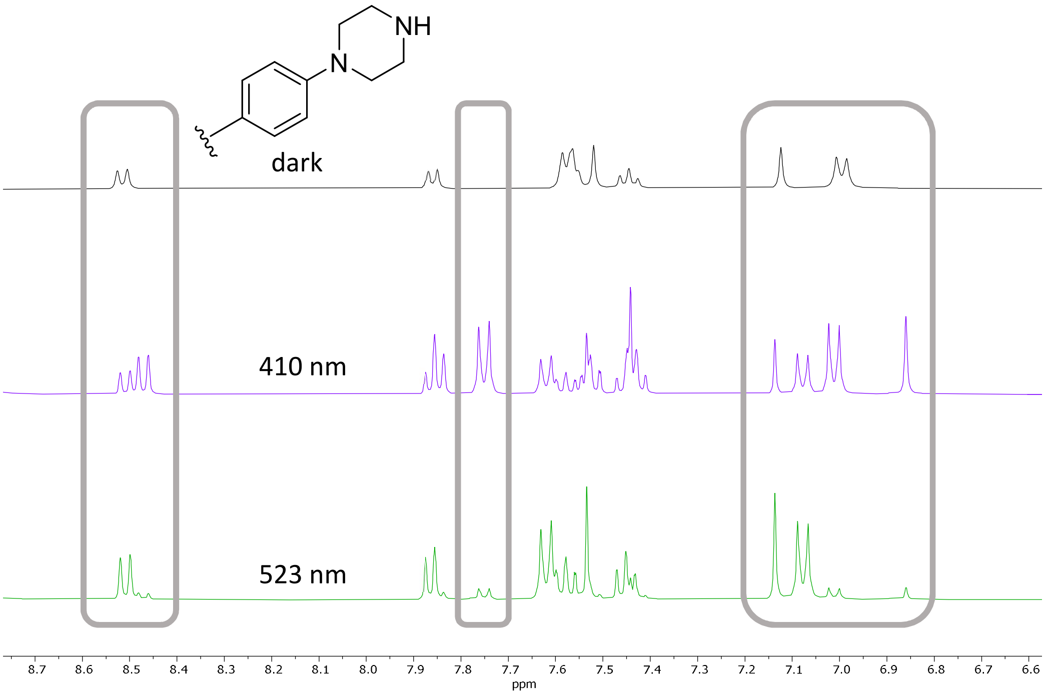

λ_{irrad}	410 nm	430 nm	455 nm	470 nm	490 nm	523 nm
**%*E***	63	57	42	33	20	13

Figure S16*: ^{1}H-NMR of* ***7*** *in DMSO-d6 prior to irradiation and after illumination to equilibrium with the indicated wavelengths (top). Concentration of* ***E-7*** *in equilibrium under illumination with the indicated wavelengths (bottom).*

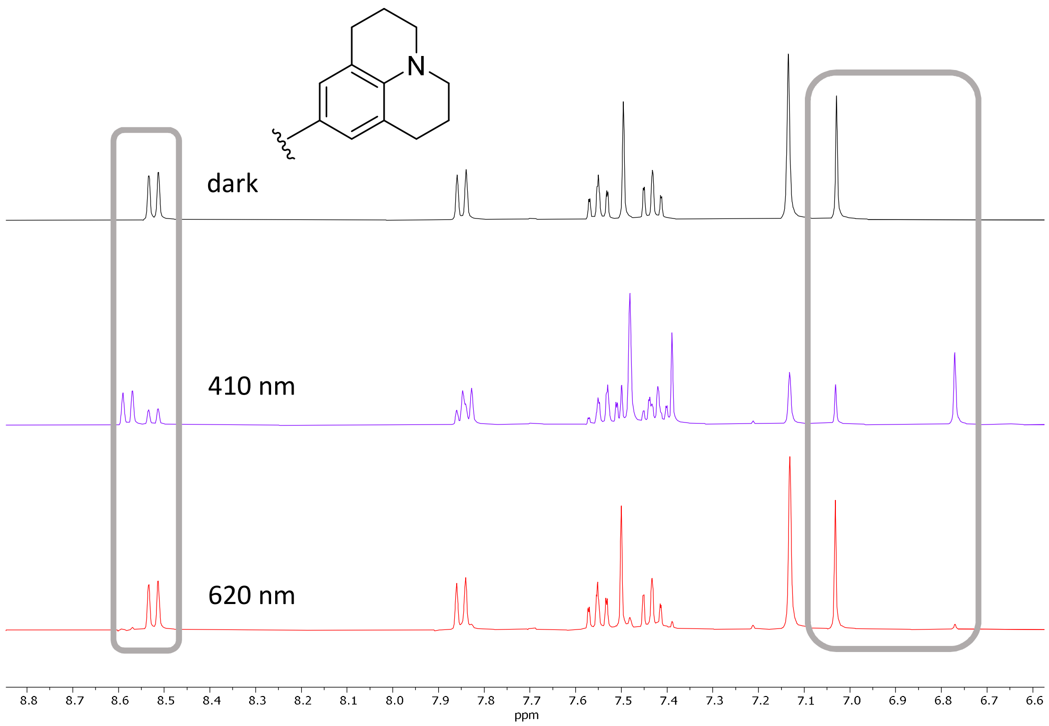

λ_{irrad}	410 nm	430 nm	455 nm	470 nm	490 nm	523 nm	590 nm	620 nm
**%*E***	64	60	57	53	37	26	15	8

Figure S17: *^{1}H-NMR of **8** in DMSO-d6 prior to irradiation and after illumination to equilibrium with the indicated wavelengths (top). Concentration of **E-8** in equilibrium under illumination with the indicated wavelengths (bottom).*

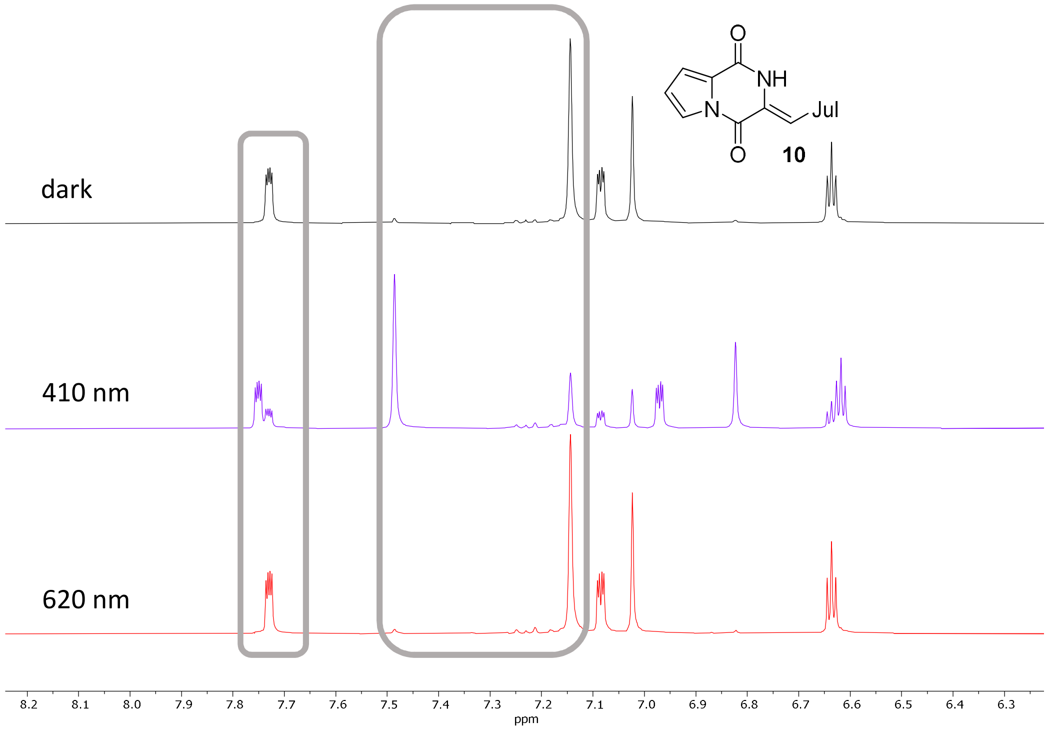

λ_{irrad}	410 nm	430 nm	455 nm	470 nm	490 nm	523 nm	590 nm	620 nm
**%*E***	70	n.d.	n.d.	n.d.	n.d.	n.d.	n.d.	3

Figure S18: *^{1}H-NMR of **10** in DMSO-d6 prior to irradiation and after illumination to equilibirium with the indicated wavelenghts (top). Concentration of **E-10** in equilibrium under illumination with the indicated wavelengths (bottom).*

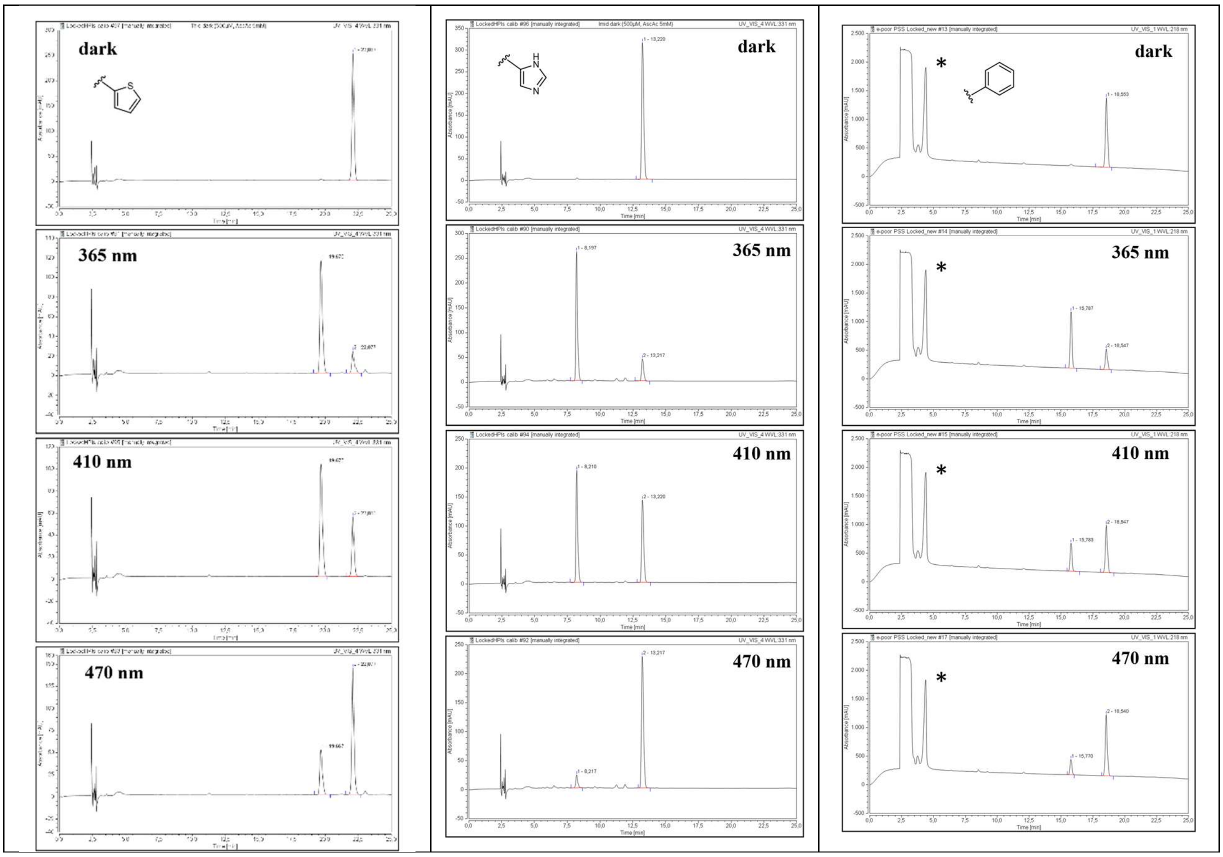

λ_{irrad}	365 nm	410 nm	470 nm
**%*E*-2**	89	78	59

λ_{irrad}	365 nm	410 nm	470 nm
**%*E*-3**	86	55	28

λ_{irrad}	365 nm	410 nm	470 nm
**%*E*-5**	68	32	33

Figure S19*: Chromatograms of the respective compounds in their PSS for the denoted wavelengths of irradiation. All solutions contained 500 µM of substance and 5 mM of ascorbic acid as a stabilizing agent. A stock solution was prepared for all compounds and the solutions were subjected to HPLC chromatography in their non-irradiated state. Subsequently, the stock solutions were irradiated with 365 nm and then split into three parts, two of which were irradiated with 410 nm, 470 nm or 455 nm, depending on the compound. All solutions were injected into the HPLC with the exact same settings, so the integrals of the respective isomers could be compared in between measurements to determine the decrease in Z-Isomer (increase in E-isomer) ($I(Z)_{PSS}/I(Z)_{dark}$). * The asterisk denotes the signal of DMSO and ascorbic acid for the chromatogram of compound* ***5****.*

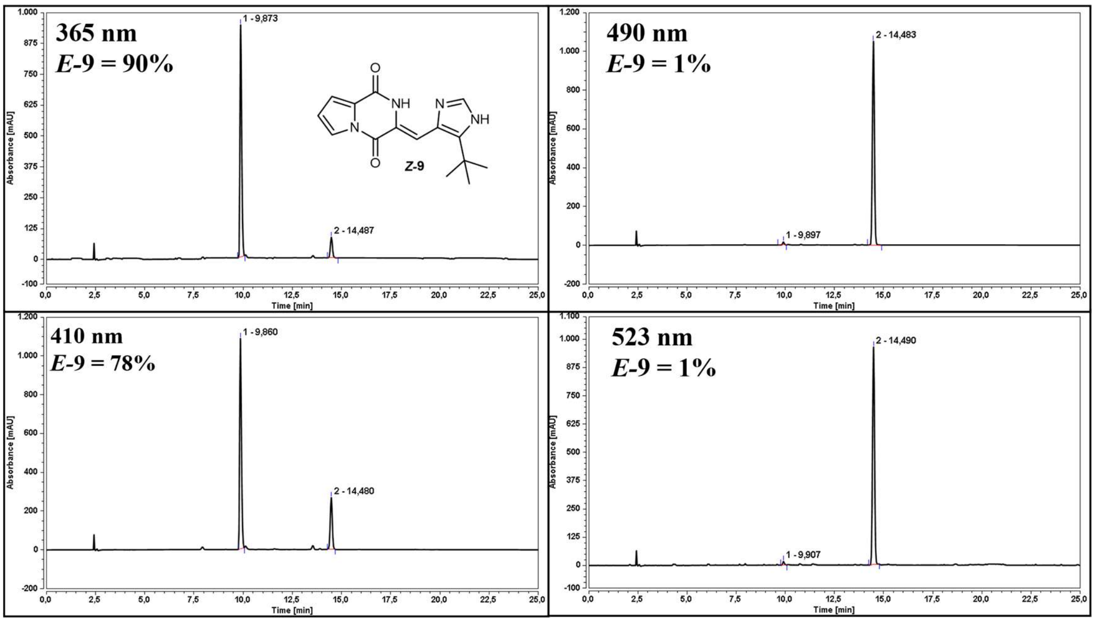

Figure S20: *HPLC-chromatograms of solutions of **9** in DMSO with 2.5 equiv. of* $AscH_2$*. First, the stock solution was irradiated with 365 nm and then split into four parts: one was quantified immediately, and the other three samples were then illuminated with the indicated wavelength until the PSS was reached for each.*

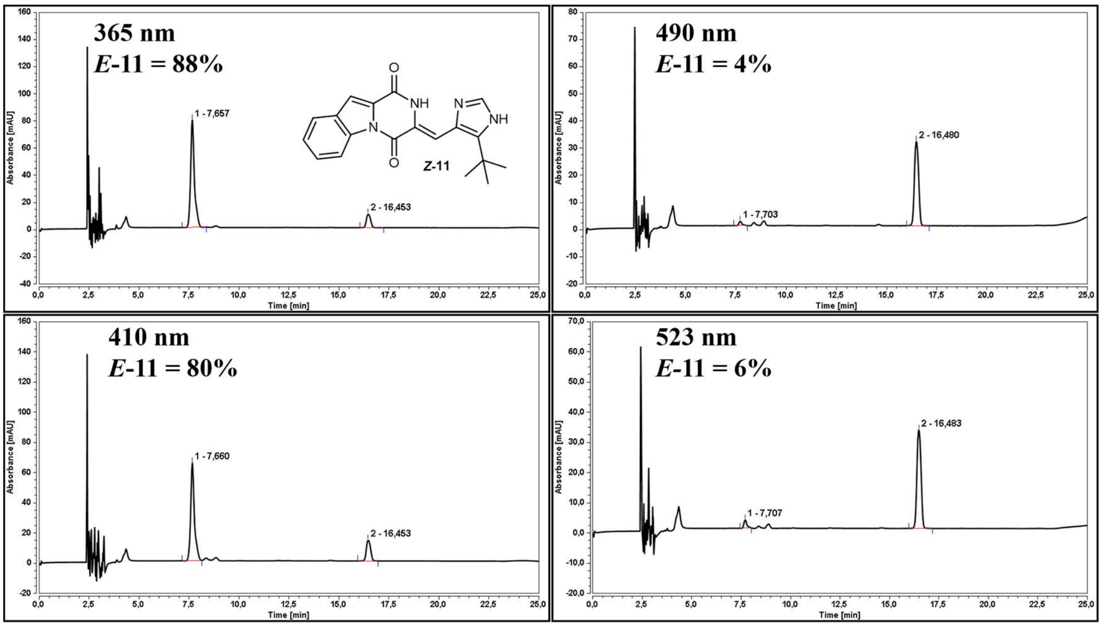

Figure S21: *HPLC-chromatograms of solutions of **11** in DMSO with 2.5 equiv. of* $AscH_2$*. First, the stock solution was irradiated with 365 nm and then split into four parts: one was quantified immediately, and the other three samples were then illuminated with the indicated wavelength until the PSS was reached for each.*

Influence of pH on Isomerization Ratios

To determine, whether the pH has an effect on isomerization ratios of compound **4**, solutions were prepared, containing a concentration of 100 µM **4** in mixtures of buffer solutions with 10 vol% of DMSO. The pH of the buffer solutions were adjusted with a pH-electrode to 5.5, 7.5 and 8.5 , using Na_2HPO_4 + citric acid, Tris + NaCl, and Tris + AcOH, respectively).

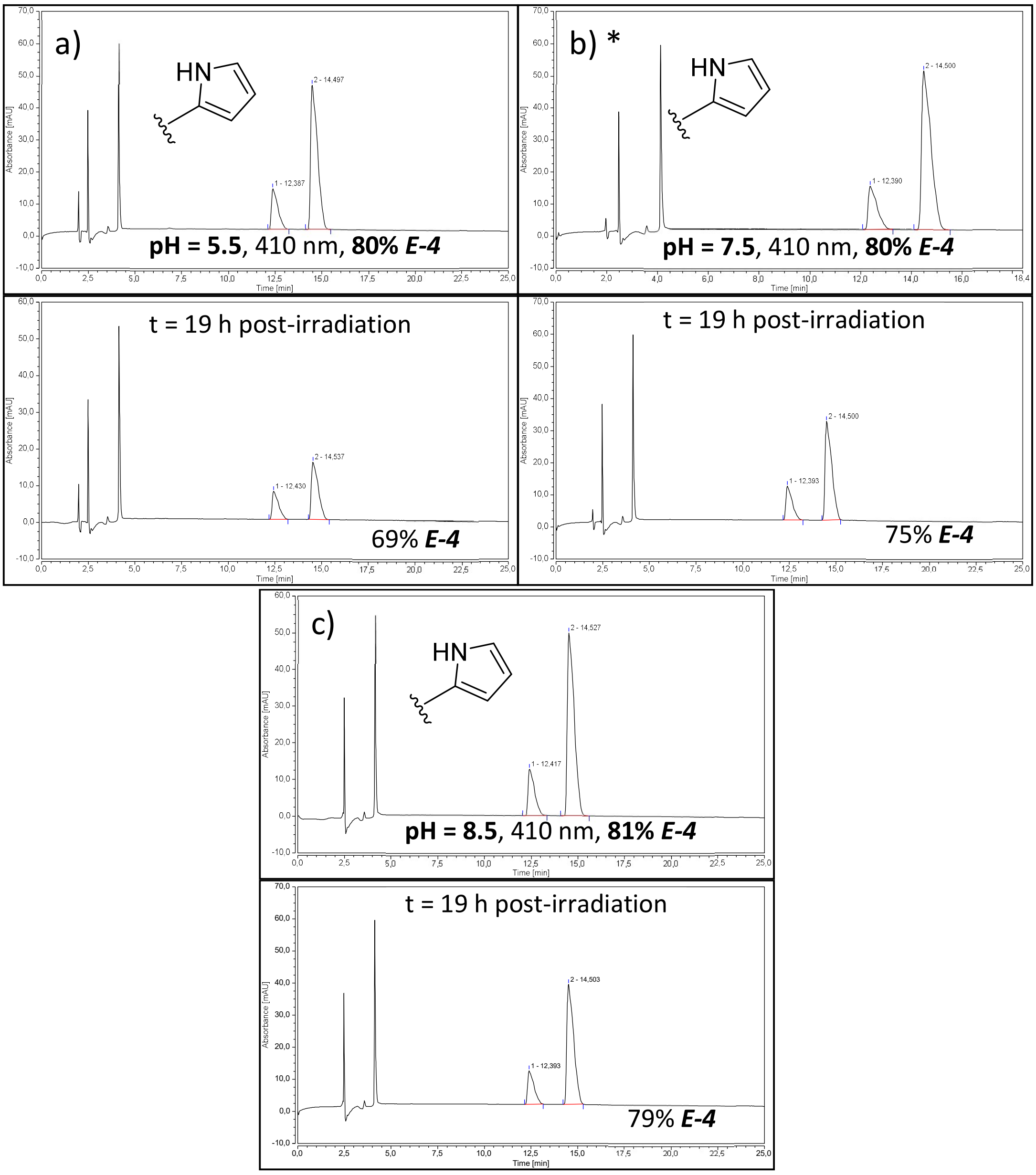

Figure S22: *HPLC-Chromatograms of* ***4****, dissolved in the respective buffer-solutions, containing 10 vol% of DMSO, immediately after equilibration to the PSS and after 19 h post-illumination. The solutions were kept at room temperature. a) pH = 5.5; b) pH = 7.5; c) pH = 8.5.* Please note, that the HPLC-run was kept shorter; the retention times of* ***Z-4*** *and* ***E-4*** *are the same, however.*

Thermal Stability of *E*-Isomers

Compounds **2**, **4** and **5** were chosen as representative examples and dissolved in DMSO with a final concentration of 1 mM. The solutions were irradiated with 365 nm for 15 min and then incubated at 50 °C for a total of 238 h. During this time, small aliquots of 200 µL were taken, diluted with 1000 µL of DMSO and analyzed *via* HPLC to follow the decrease in amount of *E*-isomer present in the isomeric mixture.

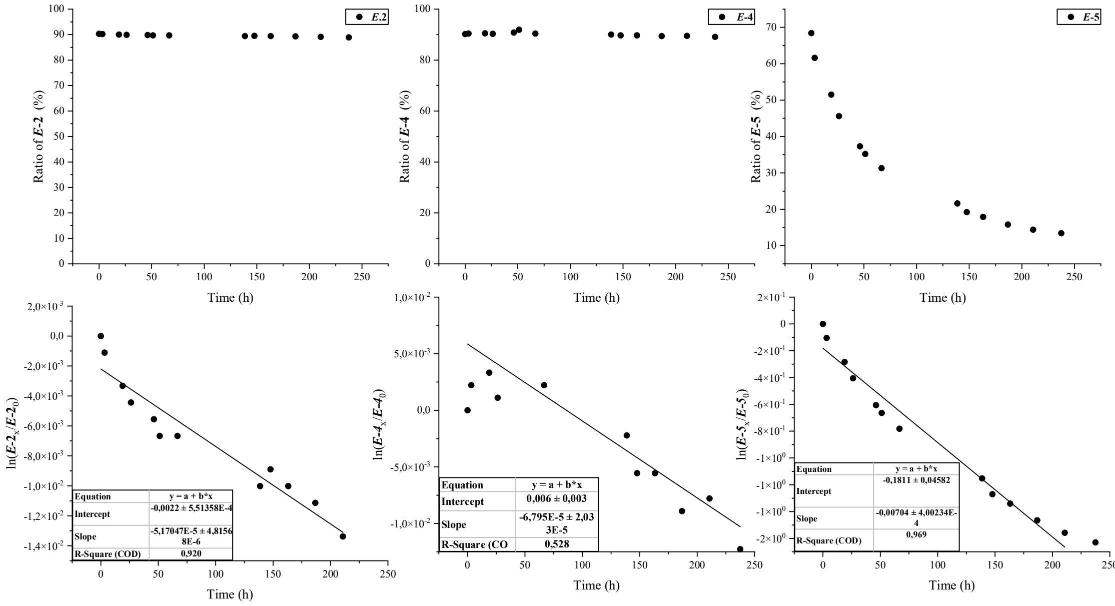

***Figure S23**: Ratio of E-Isomer, monitored over time in solutions of compounds **2** (left), **4** (middle) and **5** (right) in DMSO, irradiated with 365 nm for 15 min and subsequently kept at 50 °C (top). Linearization of the ratios of E-isomer and linear regression to determine the reaction constant of thermal isomerization, k (bottom).*

Assuming first order kinetics, the data points from Fig. S19 were linearized using the following formula:

$$\frac{ln([E_t])}{ln([E_0])} = -kt$$

Subsequently, a linear regression was performed and the negative slope of the thusly obtained straight lines was substituted for k to obtain $t_{1/2}$, employing the following expression:

$$t_{1/2} = \frac{ln(2)}{k}$$

***Table S2:** Thermal half-lives of compounds **E-2**, **E-4**, and **E-5** in DMSO at 50 °C.*

**Compound**	***E*-2**	***E*-4**	***E*-5**
**$t_{1/2}$ (DMSO, 50 °C)**	13.406 h	10.200 h	99 h

Isomerization Quantum Yield

The maximum isomerization quantum yield was determined, using the experimental setup developed by Riedle and coworkers.[3] The following LEDs with the denoted nominal emission maxima were used:

Compound **4**:

Z → E: 400 nm; Luxeon LHUV-0400-0450; **E → Z**: 470 nm; Osram OSLON SSL80 LBCP7P-GYHY

Compound **8**:

Z → E: 420 nm; Luxeon LHUV-0420-0650; **E → Z**: 592 nm; Osram OSLON SSL80 LYCP7P-JRJT

First, the respective Z-isomer was dissolved in DMSO and diluted to a final concentration of 40 µM and illuminated with 400 nm/420 nm for defined time intervals, after which a UV-Vis absorption spectrum was recorded (Fig. S20, left).

To determine the *Z*/*E*-ratio after each illumination interval, the molar absorptivity of the *E*-isomer had to be determined first. To achieve this, the sample was analzed with HPLC, to correlate the isomer ration with the last absorption spectrum. Thus ε_E can be determined (equation 1), with which the isomer-ratio can be calculated for each illumination step during measurement, using equation 2:

$$\varepsilon_Z(\lambda)=\frac{1}{a}\cdot(\varepsilon_{PSS}(\lambda)\text{-}b\cdot\varepsilon_E(\lambda)) \tag{1}$$

$$c_E=\frac{A_{tot}\text{-}\varepsilon_Z c_{tot}}{\varepsilon_E\text{-}\varepsilon_Z} \tag{2}$$

Where ε denotes the extinction coefficient of the specified species (or the PSS), c is the concentration of the respective species, A_{tot} is the total absorption of the isomeric mixture, *a* is defined as the percentage of the product at the PSS and *b* as the percentage of the substrate at the same specified PSS.

To determine the reaction quantum yield in the E→Z direction, the same sample was subsequently irradiated with 470 nm (in case of compound **4**, Fig.S20, top).or 592 nm (in case of compound **8**, Fig. S21, top).

Since continuous irradiation well after the PSS is reached will eventually lead to a measured reaction quantum yield of $\phi_{t\rightarrow\infty}$=0, only samples were taken into account, which still lie in the linear regime of the reaction progress, which ensures that Φ_{max} can be determined accurately.

Having obtained the product concentrations for the defined illumination intervals, they were plugged into equation 3.

$$\Phi= N_A hc\frac{c_{prod}V}{P_{\mathrm{abs}}\Delta t\lambda_{LED}} \tag{3}$$

Where Φ signifies the reaction quantum yield, N_A is Avogadro's constant, h is Planck's constant, c denotes the speed of light, c_{prod} stands for the product concentration formed by reaction progress, V is the sample volume, P_{abs} is the absorbed light power, Δt denotes the respective time interval and λ_{LED} stands for the nominal emission wavelength of the respective LED used for the experiment.

The values obtained were finally averaged and their standard deviation was calculated (Fig.S20, S21 bottom).

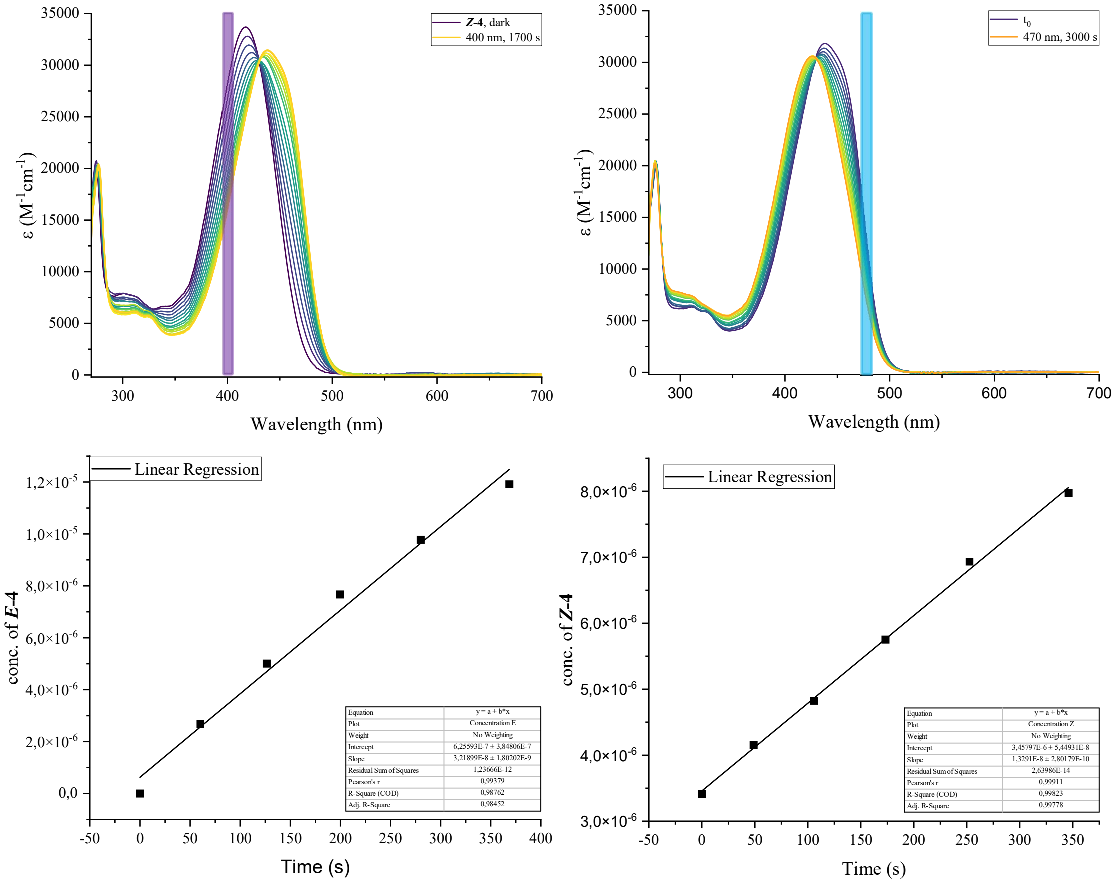

Figure S24*: UV-Vis absorption spectra of compound **4** (DMSO, 40 µM, 10.0 equiv. of AscH$_2$), continuously irradiated with 400 nm and the linear regime of reaction progress, used to assess Φ (left). UV-Vis absorption spectra of **4** for the reverse swtiching direction, under illumination with 470 nm and the respective linear regime of reaction progress (right).*

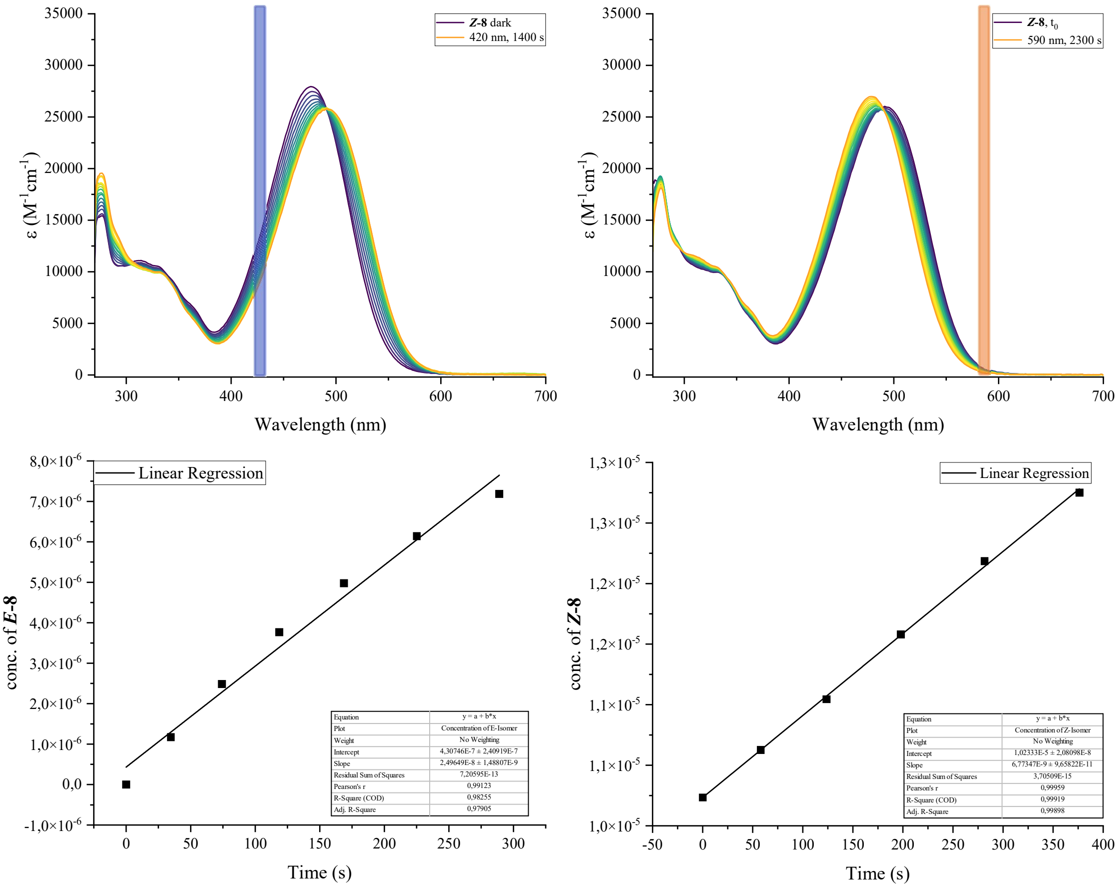

***Figure S25**: UV-Vis absorption spectra of compound **8** (DMSO, 40 µM, 10.0 equiv. of $AscH_2$), continuously irradiated with 420 nm and the linear regime of reaction progress, used to assess Φ (left). UV-Vis absorption spectra of **8** for the reverse swtiching direction, under illumination with 590 nm and the respective linear regime of reaction progress (right).*

Cell cultures

HeLa cells were cultured in Dulbecco's modified Eagle's medium (DMEM; Gibco® Life Technologies), supplemented with 10% fetal calf serum (FCS; Gibco® Life Technologies) and 1% penicillin/streptomycin (Gibco® Life Technologies), at 37°C, 5% CO_2 and saturated humidity. The cells were maintained in 75 cm^2 culture flasks (Greiner Bio-One™, CELLSTAR™). For enzymatic detachment of the cells, the culture medium was removed, and the cells were washed once with 10 ml Dulbecco's phosphate-buffered saline (DPBS; Gibco® Life Technologies) and incubated with 2 ml 0.25% trypsin-EDTA (Gibco® Life Technologies) for 3 min at 37°C. Detachment was stopped by the addition of 8 ml DMEM.

Cytotoxicity Assay

Cytotoxicity of the compounds was determined using the MTT assay. For this 100 µL of a 1 x 10^5 cells/mL cell suspension per well were seeded in a 96-well plate (Greiner Bio-One™ CELLSTAR™) and incubated overnight at 37°C. The following day, the cells were treated with the compounds **4** respectively **8** (0,1 µM; 0,2 µM; 0,5 µM; 1 µM, 2 µM, 5 µM, 10 µM; dissolved in DMEM), adding 100 µL of each solution to the cells in triplicates. Live and dead controls were included in triplicates, where a media change was performed on day 2. After an incubation period of 72 h, dead controls were prepared by adding 5 µL of a 20% Triton X-100 (Carl Roth® GmbH) solution in H_2O. Next, 15 µL of 3-(4,5-dimethylthiazol-2-yl)-2,5-diphenyltetrazolium bromide reagent (MTT reagent; Promega) was added to each well and incubated for 3 h. After incubation 100 µL of Stop Solution (Promega) was added and the plate was incubated for additional 24 h. Absorbance was measured at 595 nm using a microplate reader (SpectraMax iD3). For data analysis, the absorbance values of the dead controls were subtracted, and the relative viability was calculated by normalizing to the mean value of the live controls.

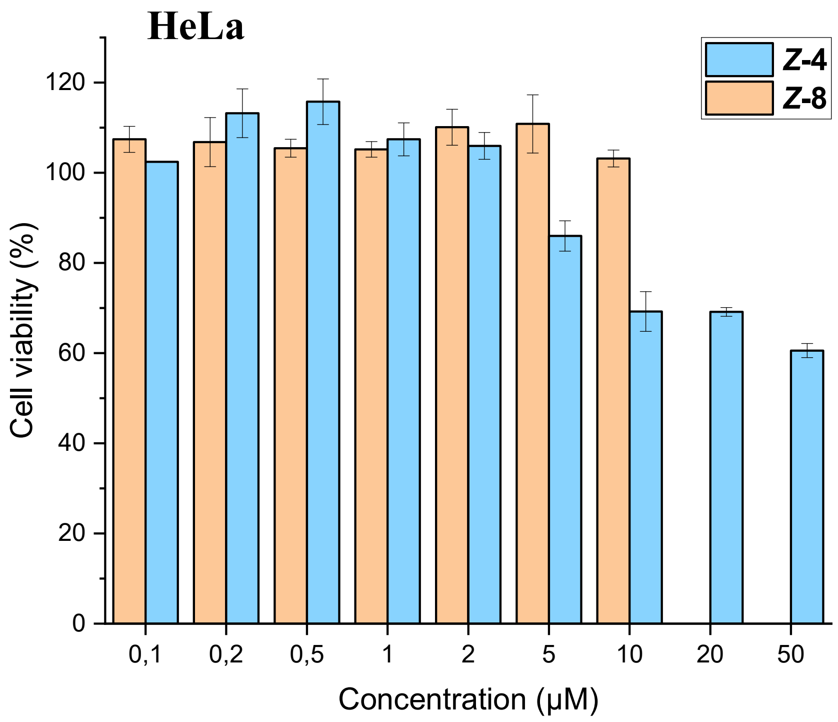

***Figure S26**: **Cytotoxicity Assay.** Viability of HeLa cells after treatment with the compounds **4** (blue bars) and **8** (orange bars) for 72 hours. The measurements were performed in triplicates. The data are presented as means ± standard deviation (SD).*

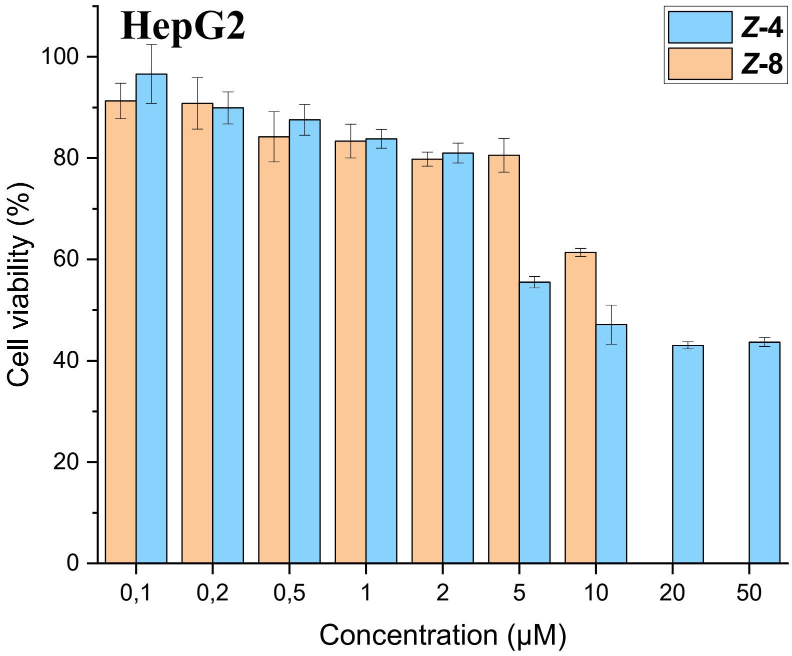

***Figure S27**: **Cytotoxicity Assay.** Viability of HepG2 cells after treatment with the compounds **4** (blue bars) and **8** (orange bars) for 72 hours. The measurements were performed in triplicates. The data are presented as means ± standard deviation (SD).*

Cytotoxicity of the compounds was additionally determined using a slightly different protocol for the MTT-assay, for the sake of reproducibility:

100 µL of a 3 x 10^5 cells/mL cell suspension per well were seeded in a 96-well plate (Greiner Bio-One™ CELLSTAR™) and incubated overnight at 37 °C. The following day, the cells were treated with the compounds **4** and **8** respectively (0,2 µM; 0,5 µM; 1 µM; 10 µM; 20 µM and 50 µM only for compound **4** due to solubility limitations of compound **8**), adding 100 µL of each solution to the cells in six technical replicates. For this, stock solutions of the compounds in DMSO were diluted in DMEM to a final DMSO concentration of 0.5 %. Live and dead controls were included in six technical replicates, and the medium of the control wells was exchanged with DMEM containing 0.5 % DMSO. After an incubation period of 48 h, dead controls were prepared by adding 5 µL of Triton X-100 (Carl Roth® GmbH, 10 % solution in PBS (w/v)) per well. Next, 10 µL of 3-(4,5-dimethylthiazol-2-yl)-2,5-diphenyltetrazolium bromide reagent (MTT reagent; Cell Proliferation Kit I from Roche) was added to each well and incubated for 3 h. Subsequently, 100 µL of Stop Solution (Cell Proliferation Kit I from Roche) was added and the plate was incubated for an additional 72 h. The absorbance was measured at 595 nm using a microplate reader (CLARIOstar Plus). For data analysis, the absorbance values were averaged over five of the six technical replicates (since one row exhibited a systematically occurring pipetting error), the dead controls were subtracted, and the relative viability was calculated by normalizing to the mean value of the live controls. The number of independent experiments was N = 2 for compound ***Z*-4** and N = 3 for compound ***Z*-8**.

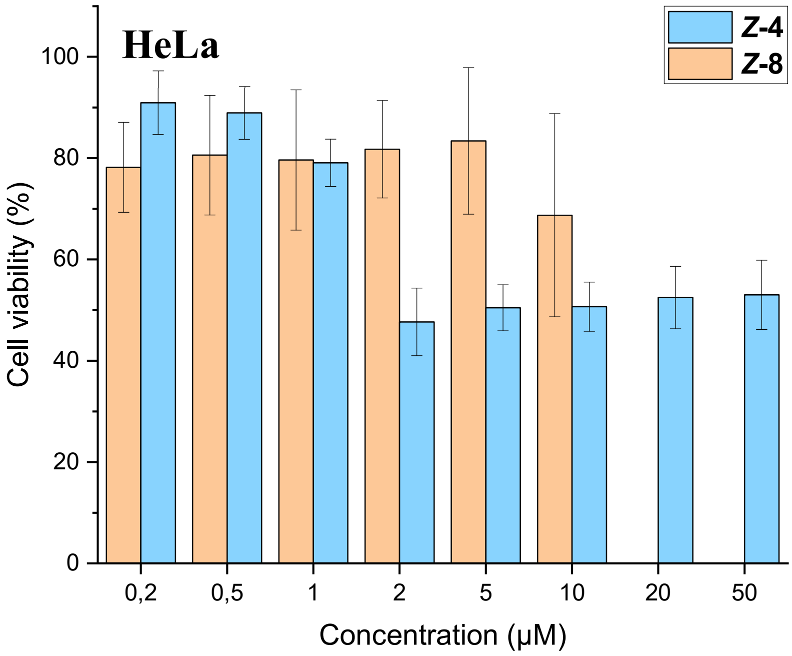

***Figure S28**: **Cytotoxicity Assay.** Viability of HeLa cells after treatment with the compounds **4** (blue bars) and **8** (orange bars) for 48 hours. The measurements were performed in triplicates. The data are presented as means ± standard deviation (SD).*

Fluorescence imaging

HeLa cells (2 x 10^4 cells) were seeded in 200 µL DMEM in one well of a µ-Slide 8 well ibiTreat (ibidi® GmbH) and incubated overnight. The following day, the medium was removed and replaced with 200 µLof 1 µM solutions of either **4** or **8** in DMEM. One well was left untreated as a negative control. Cells were incubated for 72 hours under standard culture conditions. After incubation, the solutions were removed, cells were washed with DPBS and fresh DMEM was added. All experimental steps were performed in the dark. Fluorescence imaging was conducted using the Stellaris 5 confocal microscope (Leica) with a 63x oil objective (**4**: λ_{exc}: 405 nm nm, λ_{em}: 450 nm - 650 nm; laser intensity: 1%; **8**: λ_{exc}: 510 nm, λ_{em}: 545 nm - 695 nm; laser intensity: 6.4%)

Photoswitching of 4 and 8 inside living HeLa cells

To analyze the photoswitching ability of the components in HeLa cells, initial images of the treated cells were captured to represent the starting state, where the more fluorescent Z-isomer predominates. Imaging was conducted using a Stellaris 5 microscope (Leica) with the following settings: **4**: λ_{exc}: 405 nm nm, λ_{em}: 450 nm - 650 nm; laser intensity: 1%; **8**: λ_{exc}: 510 nm, λem: 545 nm - 695 nm; laser intensity: 6.4%.

A: The cells were irradiated with the microscope's 405 nm laser for 5 minutes (laser intensity:20%) to induce isomerization to the less fluorescent E-isomer. To induce re-isomerization into the Z-isomer, the cells were irradiated again for 5 minutes using the microscope's laser (**4**: λ_{exc}: 505 nm, laser intensity: 20%; **8**: λ_{exc}: 590 nm, laser intensity: 20 %). After each irradiation step, images were captured to monitor the photoswitching process. As a control, instead of the second irradiation step to induce re-isomerization back to the Z-isomer, cells were left untreated for 5 minutes to allow thermal re-isomerization.

B: To test whether the compounds are capable of being photoswitched multiple times, the cells were initially irradiated with the microscope's 405 nm laser for 5 minutes (laser intensity: 20%) to convert the molecule into the less fluorescent *E*-isomer form. Following this, the cells were left for 5 minutes in the dark to allow thermal relaxation, back to the *Z*-isomer. The cells were then irradiated again for 5 minutes with the 405 nm laser and left in the dark for an additional 5 min. Images were captured after each irradiation step to monitor the photoswitching process.

The relative fluorescence intensity of the recorded images was quantified using Fiji software. The fluorescence intensity of the initial image was set to 100% as reference. The relative intensities of subsequent images were calculated by referencing them to the intensity of the initial image.

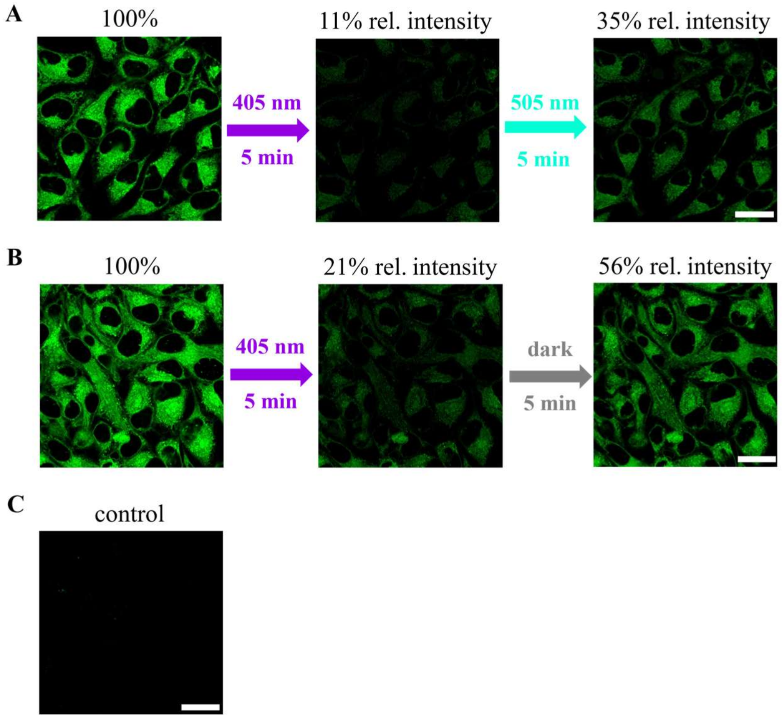

***Figure S29**: **Photoswitching of 4 inside living HeLa cells.** Cells were treated with 1 µM of 4 and incubated for 72 hours. Fluorescence imaging was performed using a Stellaris 5 confocal microscope (Leica; 63x oil objective; λ_{exc}: 405 nm, λ_{em}: 450 nm - 650 nm). **Scale bar:** 25 µM. Initial images were captured, where the more fluorescent Z-isomer predominates. Afterward, the cells were irradiated with 405 nm laser for 5 minutes to induce isomerization to the less fluorescent E-isomer. **A:** Irradiation of the cells for 5 minutes with 505 nm laser to induce re-isomerization to the Z-isomer. **B:** Cells were left for 5 minutes in the dark. **C:** control. The relative fluorescence intensity of the images was quantified using Fiji software.*

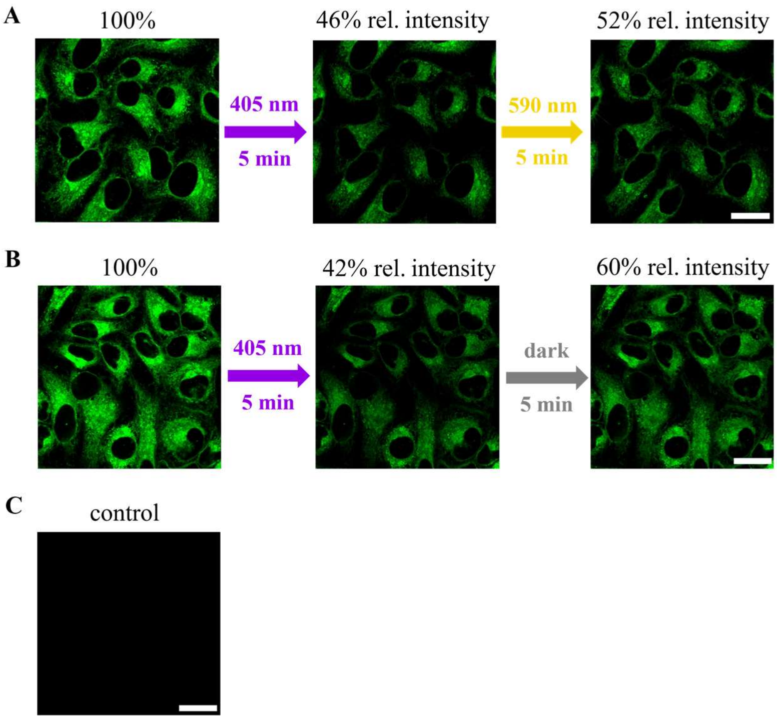

***Figure S30**: **Photoswitching of 8 inside living HeLa cells.** Cells were treated with 1 µM of **8** and incubated for 72 hours. Fluorescence imaging was performed using a Stellaris 5 confocal microscope (Leica; 63x oil objective; λex: 510 nm, λem: 545 nm - 695 nm). **Scale bar:** 25 µM. Initial images were captured, where the more fluorescent Z-isomer predominates. Afterward, the cells were irradiated with a 405 nm laser for 5 minutes to induce isomerization to the less fluorescent E-isomer. **A:** Irradiation of the cells for 5 minutes with a 590 nm laser to induce re-isomerization to the Z-isomer. **B:** Cells were left for 5 minutes in the dark. C: control. µm. The relative fluorescence intensity of the recorded images was quantified using Fiji software.*

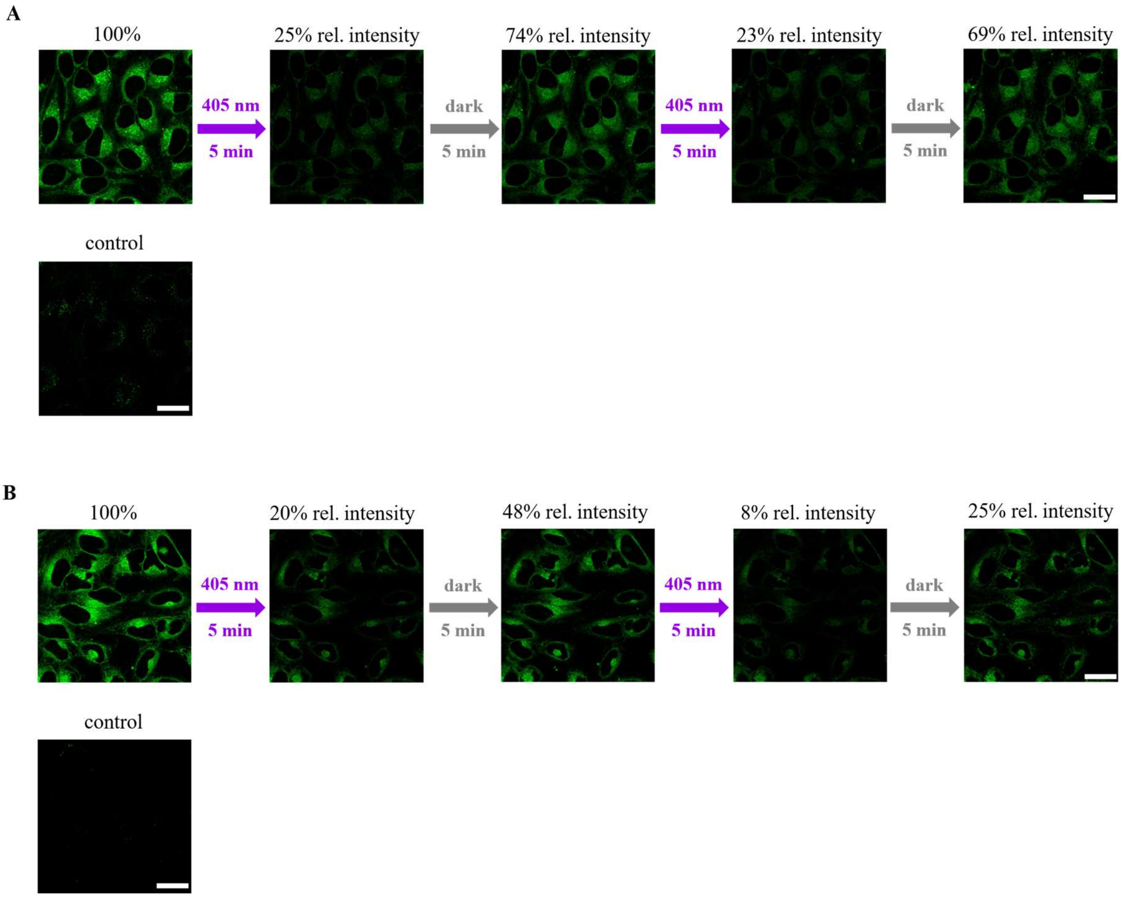

***Figure S31**: **Photoswitching of A: 4 and B: 8 inside living HeLa cells.** To test whether the compounds are capable of being photoswitched multiple times, the cells were initially irradiated with a 405 nm laser for 5 minutes (laser intensity: 20%) to convert the molecule into the less fluorescent E-isomer form. Following this, the cells were left for 5 minutes in the dark to allow thermal relaxation, back to the Z-isomer. The cells were then irradiated again for 5 minutes with the 405 nm laser and left in the dark for an additional 5 minutes. Images Fluorescence imaging was conducted using the Stellaris 5 confocal microscope (Leica) with a 63x oil objective (**A: 4**: λex: 405 nm nm, λem: 450 nm - 650 nm; laser intensity: 1%; **B: 8**: λex: 510 nm, λem: 545 nm - 695 nm; laser intensity: 6,4%). **Scale bar:** 25 µm. The relative fluorescence intensity of the recorded images was quantified using Fiji software.*

Thermal relaxation of 4 and 8 inside living HeLa cells

To investigate the thermal relaxation of the *E*-isomers in living HeLa cells, fluorescence images of the treated cells were taken using a Stellaris 5 microscope (**4**: λ_{exc}: 405 nm nm, λem: 450 nm - 650 nm; laser intensity: 1%) (**8**: λ_{exc}: 510 nm, λem: 545 nm – 695 nm; laser intensity: 6.4%). In the initial state, the more fluorescent *Z*-isomer of the compounds predominates. The cells were subsequently irradiated with the microscope's 405 nm laser for 5 min (laser intensity: 20 %) to convert the *Z*-isomer to the less fluorescent *E*-isomer. Images were captured at 5, 10, and 15-min intervals post-irradiation to monitor the relaxation process back to the more fluorescent *Z*-isomer. The relative fluorescence intensity of the recorded images was quantified using Fiji software. The fluorescence intensity of the initial image was defined as 100% and the relative intensities of subsequent images were determined by normalizing them to the intensity of the initial image.

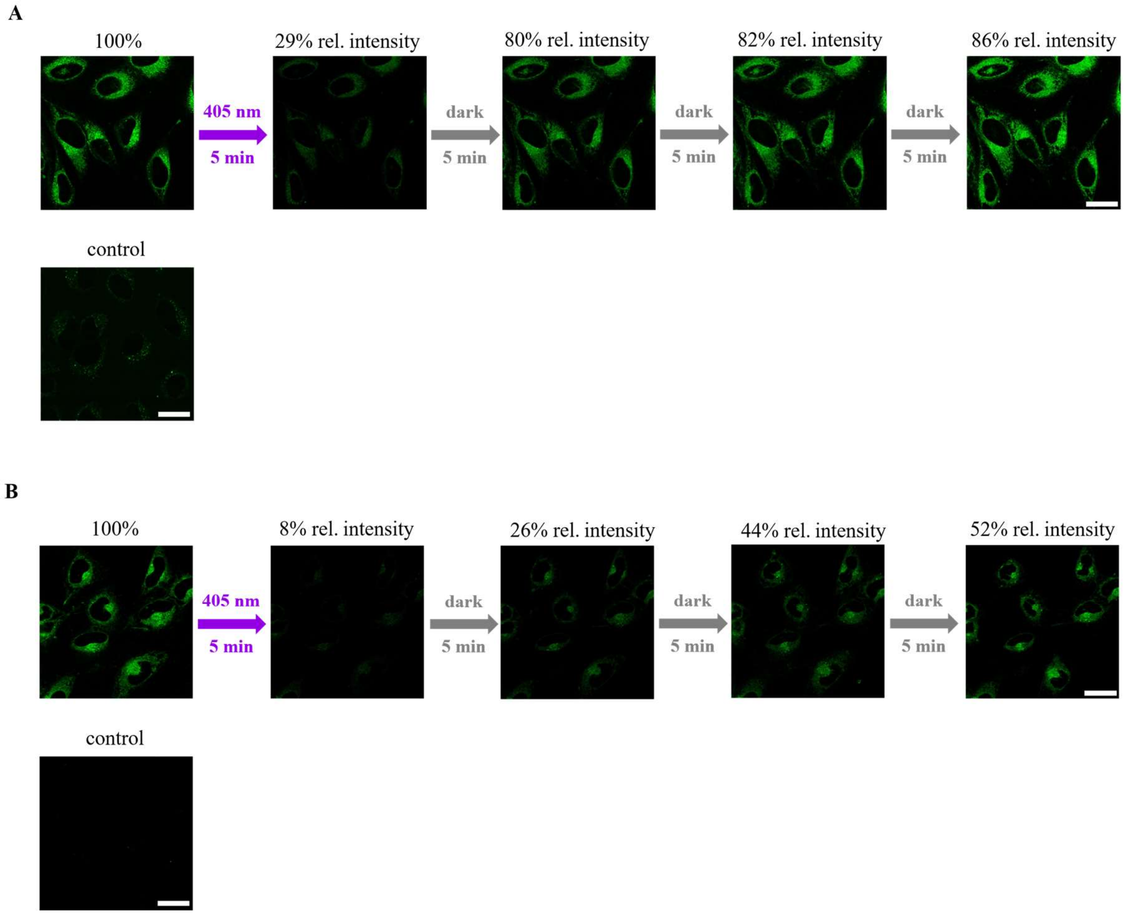

***Figure S32**: **Thermal relaxation of A: 4 and B: 8 inside living HeLa cells.** To examine the thermal relaxation of the E-isomers in living HeLa cells, fluorescence images of the treated cells (1 µM, 72 hours) were taken, where the stronger fluorescent Z-isomer predominates. The cells were subsequently irradiated with a 405 nm laser for 5 min to convert the Z-isomer to the less fluorescent E-isomer. Images were captured at 5, 10, and 15-minute intervals post-irradiation to monitor the relaxation process back to the more fluorescent Z-isomer. Fluorescence imaging was conducted using the Stellaris 5 confocal microscope (Leica) with a 63x oil objective (**A: 4**: λex: 405 nm, λem: 450 nm - 650 nm; laser intensity: 1%; **B: 8**: λex: 510 nm, λem: 545 nm - 695 nm; laser intensity: 6,4%). **Scale bar:** 25 µm. The relative fluorescence intensity of the recorded images was quantified using Fiji software.*

In Vitro Control Experiments for Thermal Relaxation

HPLC-Monitoring of Thermal Relaxation of *E*-4 in DMEM

To determine whether the observed regeneration of fluorescence is an effect of accelerated thermal relaxation in the cell culture medium, used to incubate the cells (DMEM), a 1 mM solution of **4** in DMSO was prepared, irradiated with light of 410 nm wavelength for 15 min, subsequently diluted to a concentration of 100 µM in DMEM (10% (v/v) DMSO) and kept at 37.5 °C for a total of 5.5 h. Small aliquots were taken after roughly every hour and analyzed *via* HPLC to determine the isomeric ratio. No change was observed over the course of the experiment.

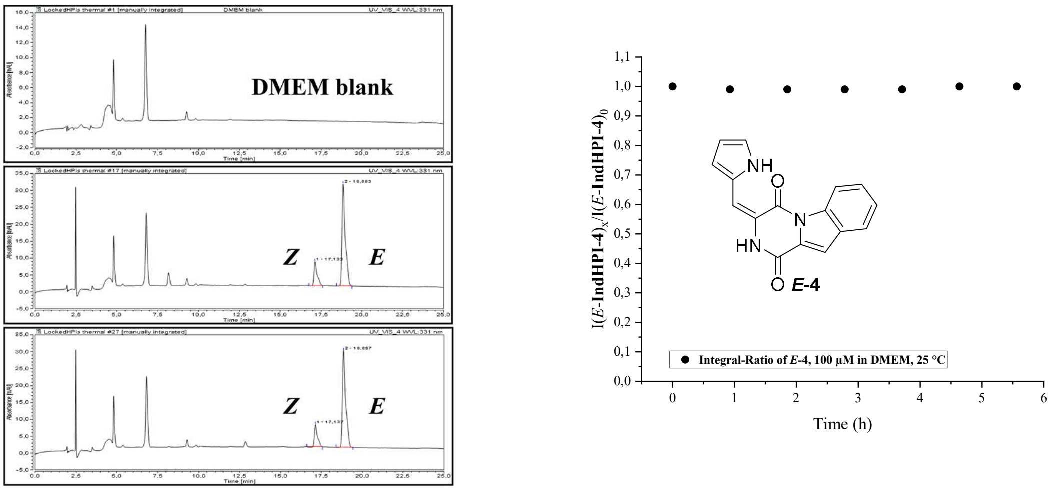

Figure S33: ***a*** *HPLC chromatograms of pure DMEM (top),* ***4*** *after irradiation with 410 nm for 15 min, dilution with DMEM and incubation at 37.5 °C for 1 h (middle),* ***4*** *in DMEM after another 5 h of incubation at 37.5 °C (bottom).* ***b*** *Ratio of integrals for* ***E-4*** *(I_x/I_0) over the course of 5.5 h of incubation.*

UV-Vis-Monitoring of Thermal Relaxation of *E*-4 in DMEM in Presence of GSH

In order to test the stability of the *E*-isomer of IndHPIs in respect to nucleophilic species, compound **4** was taken as a represantative example, and dissolved in DMEM (1 vol% DMSO) to a final concentration of 20 µM, with or without GSH as an additive with 2 mM concentration. The solutions were illuminated with 410 nm for 5 s and incubated at 37.5 °C and a UV-Vis spectrum was recorded every 30 s for up to 30 min.

To validate the regeneration of the thermally stable Z-isomer, a saturated solution (<200 µM) in DMEM (10 vol% DMSO) was prepared, illuminated with 410 nm for 10 s and subsequently aliquots were drawn after the indicated time intervals and subjected to HPLC-analysis. The sample was kept at room temperature.

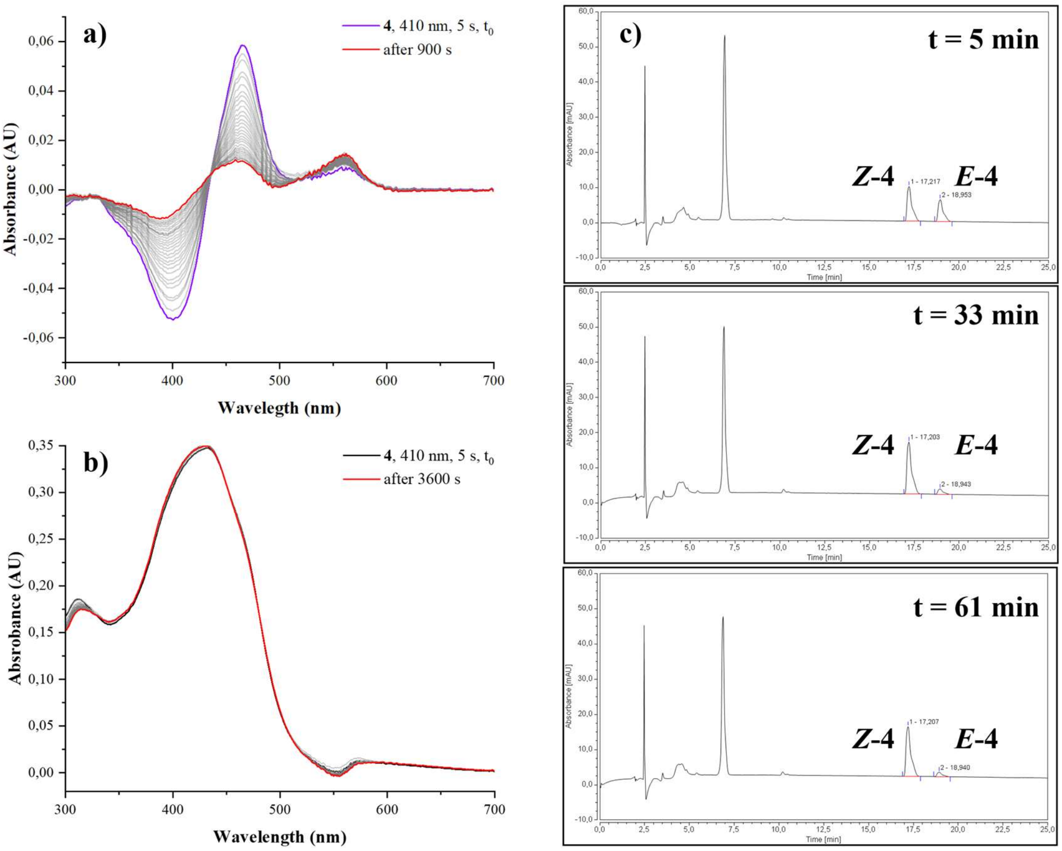

***Figure S34**: **a)** UV-Vis spectra of **4** (20 µM, DMEM, 1 vol% DMSO) in the presence of GSH (2 mM), illuminated with 410 nm for 5 s, blanked against the same, non-illuminated solution (violet). The solution was kept at 37.5 °C and a spectrum was recorded every 30 s for 15 min (grey and red, indicating the end-point of monitoring). **b)** UV-Vis spectra of **4** (20 µM, DMEM) without GSH as additive, illuminated with 410 nm for 5 s and incubated at 25 °C (black) and monitored every minute over the course of 30 min (grey and red). **c)** HPLC-traces of a saturated solution of compound **4** (<200 µM), dissolved in DMEM (10 vol% DMSO) in the presence of 2 mM GSH and after illumination with 410 nm. Aliquots were drawn and analyzed after the indicated time post irradiation. The solution was kept at room temperature.*

Normalized UV-Vis and Fluorescence Spectra

All compounds **1-11** were each dissolved in DMSO with 10.0 equiv. of $AscH_2$ to a resulting final concentration of 40 µM (d = 5 mm). The absorption, as well as the fluorescence spectra were measured and normalized.

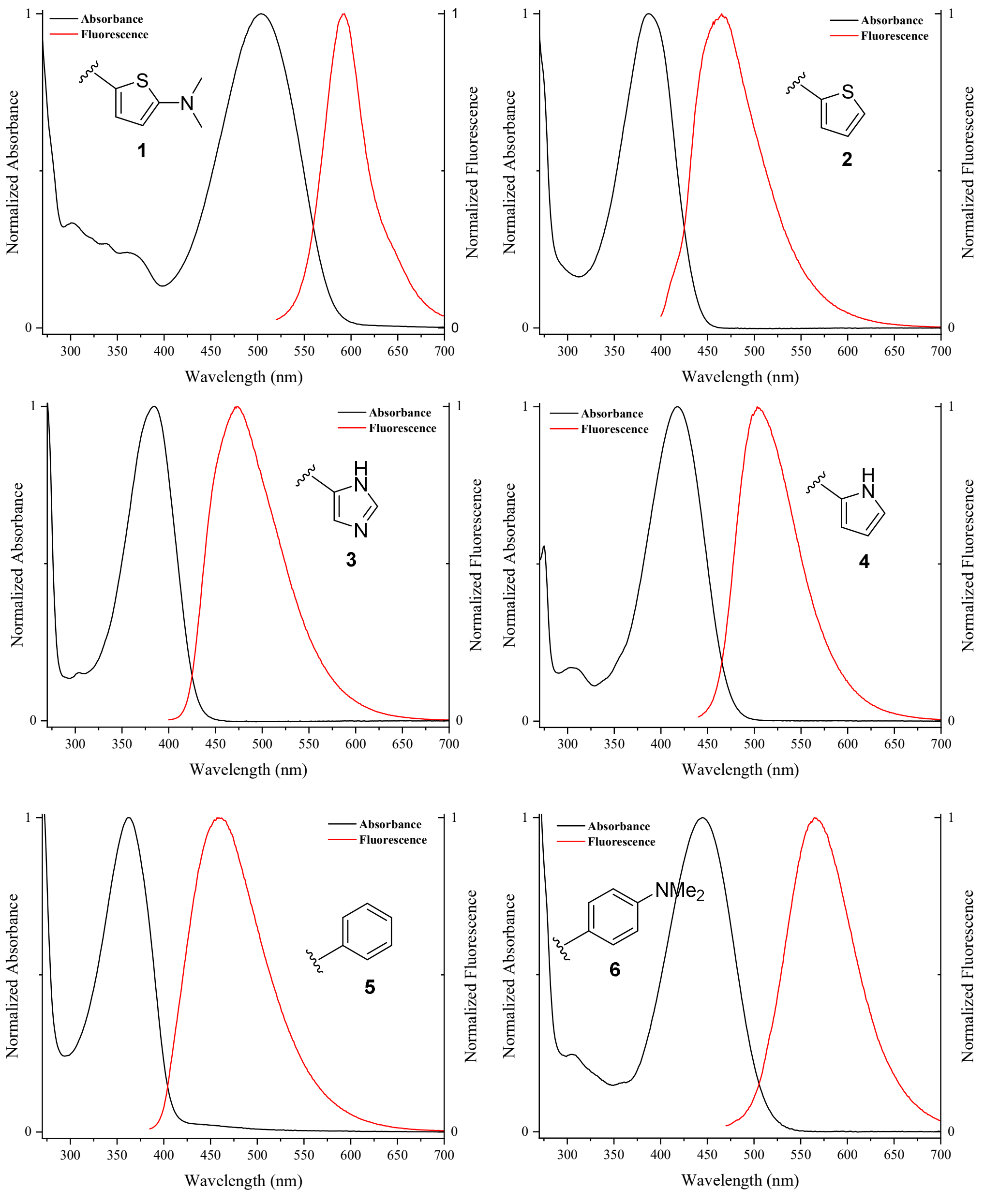

***Figure S35**: Normalized Absorbance and Fluorescence spectra of compound **1-6** in DMSO with 10.0 equiv. of $AscH_2$, at a concentration of 40 µM.*

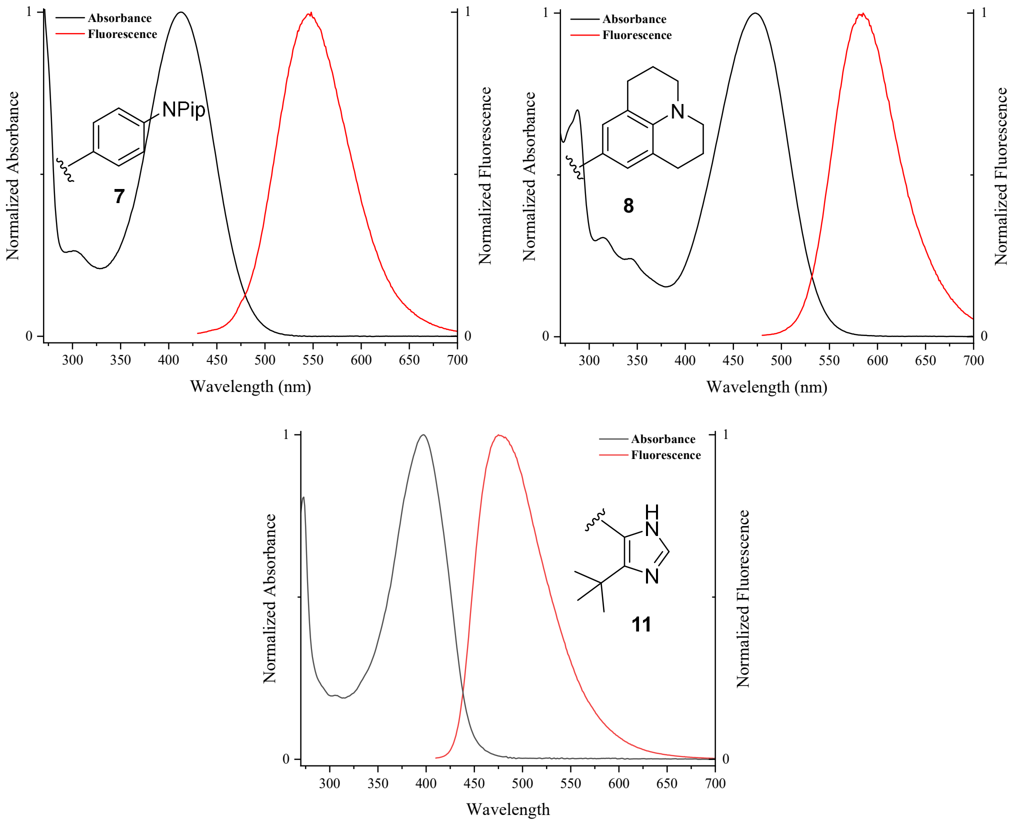

***Figure S36**: Normalized Absorbance and Fluorescence spectra of compound **7**, **8** and **11** in DMSO, with 10.0 equiv. of $AscH_2$ at a concentration of 40 µM.*

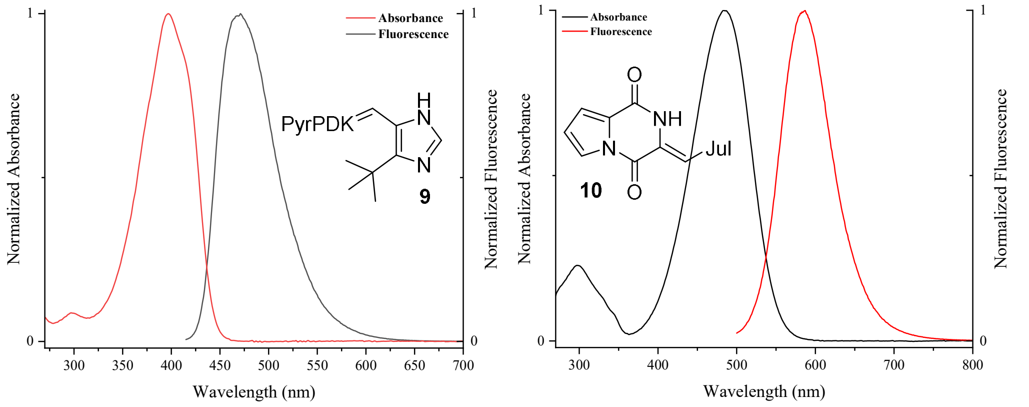

***Figure S37**: Normalized Absorbance and Fluorescence spectra of compound **9** and **10** in DMSO, with 10.0 equiv. of $AscH_2$ at a concentration of 40 µM.*

***Table S3**: Comparison of absorption- and emission maxima of compounds 1-11 in DMSO with an additional 10.0 equiv. of $AscH_2$ at 40 µM and their resulting Stokes-Shift.*

Compound	$\lambda_{max,abs.}$ (nm)	$\lambda_{max,em.}$ (nm)	Δ (nm)
**1**	504	593	89
**2**	388	465	77
**3**	385	473	88
**4**	418	503	85
**5**	363	462	99
**6**	446	549	103
**7**	415	550	135
**8**	471	577	103
**9**	397	471	74
**10**	483	587	104
**11**	397	475	78

Fluorescence Quantum Yield

To determine the fluorescence quantum yield (Φ_{fl}) of the compounds as their pure *Z*-isomers, solutions were prepared, with concentrations, such that their absorption falls below 0.1 AU (4 - 6 µM in DMSO), which is the threshold to eliminate inner filter effects, as recommended by IUPAC.[4] Depending on the excitation and emission properties of the respective compound, an appropriate refence was chosen ($Ru(bpy)_3$, Rhodamine 6G or Perylene). Both the compound and its reference were excited at the same wavelength and their fluorescence spectra were recorded with constant settings. Subsequently, the collected spectra were integrated *via* Origin and the thus obtained values were put into the following formula:

$$\phi_{fl}^{S} = \frac{\eta_S^2}{\eta_R^2} \cdot \frac{\int_0^\infty I_{fl}^S(\lambda_{ex}, \lambda_{em}) d\lambda_{em}}{\int_0^\infty I_{fl}^R(\lambda_{ex}, \lambda_{em}) d\lambda_{em}} \cdot \frac{(1\text{-}10^{A^{R\,(\lambda_{ex})}})}{(1\text{-}10^{A^{S\,(\lambda_{ex})}})} \cdot \phi_{fl}^{R}$$

Where η_x denotes the refractive index of the solvent that was used for either the sample S or reference R, $\int_0^\infty I_{fl}^X(\lambda_{ex}, \lambda_{em}) d\lambda_{em}$ represents the integrated area of the respective fluorescence spectra and $A^{X\,(\lambda_{ex})}$ the absorbance value at the excitation wavelength.

To determine the quantum yields of the isomeric mixtures, the initially determined ϕ_{fl} of the non-illuminated samples was used as a reference value. All solutions were irradiated with their most efficient isomerization wavelengths for switching to the *E*-, as well as back to the *Z*-isomer and their fluorescence spectra and evaluated as described above.

Table S4: *Ratios of E-isomer of the presented compounds after illumination with the denoted wavelengths. Φ_{fl} was determined for the pure Z-isomers and after enrichment of as much E-isomer as possible $\Phi_{fl,relative}$ was determined. Upon irradiation with a wavelength that provides the highest degree of back-isomerization $\Phi_{fl,recovered}$ was evaluated.*$\Phi_{fl,recovered}$ > 0.99 is the result of an artifact of the inner-filter effect.*

Compound	%*E* (forward)	%*E* (backward)	ϕ_{fl}(%)	$\phi_{fl,\,relative}$(%)	$\phi_{fl,\,recovered}$(%)
**1**	74 (410 nm)	22 (620 nm)	2.3	0.87	0.97
**3**	86 (365 nm)	28 (470 nm)	15	0.31	0.94
**2**	89 (365 nm)	59 (470 nm)	1.5	0.89	0.97
**4**	73 (410 nm)	26 (523 nm)	11	0.87	0.99
**5**	71 (365 nm)	17 (455 nm)	0.8	0.95	0.95
**6**	62 (410 nm)	23 (523 nm)	2.4	0.92	0.97
**7**	63 (410 nm)	13 (523 nm)	0.6	0.96	0.98
**8**	64 (410 nm)	8 (620 nm)	1.4	0.93	1.00
**9**	90 (365 nm)	1 (523 nm)	13	0.33	1.06*
**10**	70 (410 nm)	3 (620 nm)	n/d	n/d	n/d
**11**	88 (365 nm)	1 (523 nm)	22	0.46	0.99

NMR Spectra

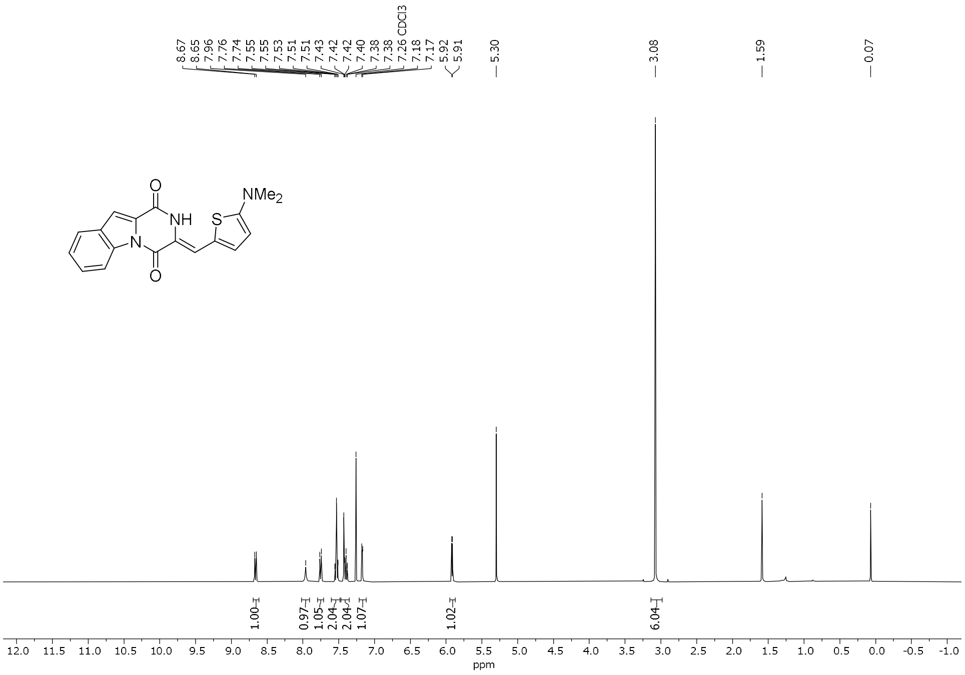

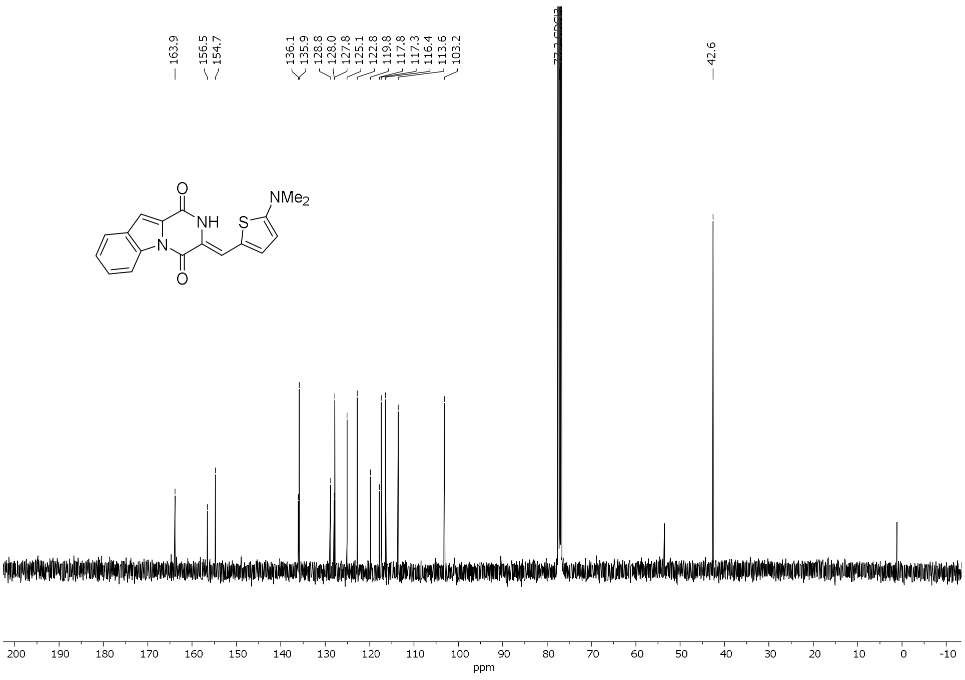

Figure S38: *^{1}H-NMR (400 MHz, top) and 13C-NMR (101 MHz, bottom) spectra in $CDCl_3$ of compound* ***Z-1***.

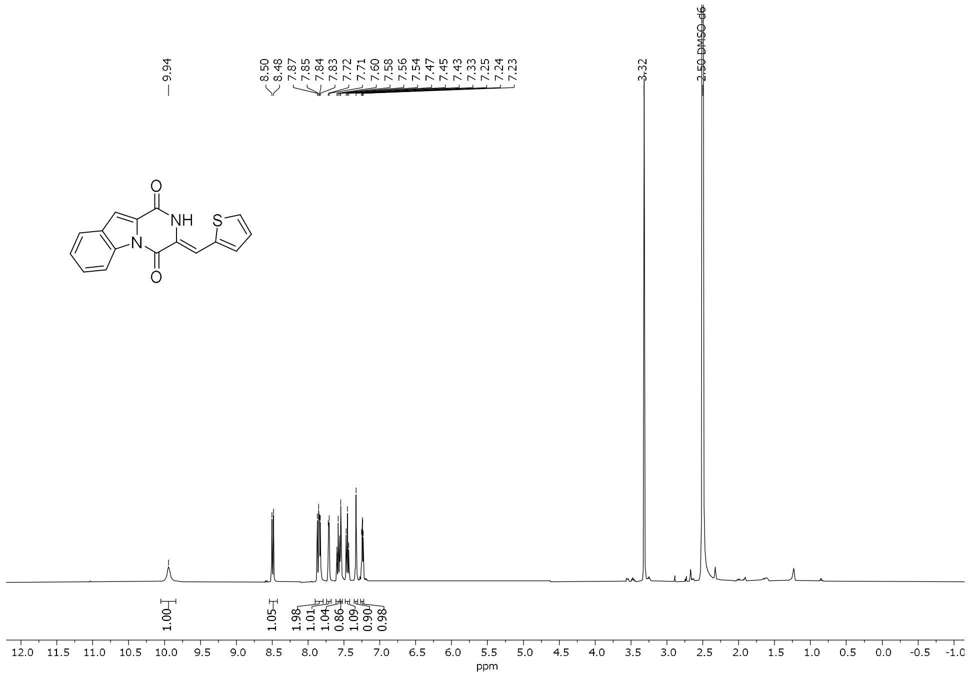

Figure S39 : *^{1}H-NMR spetrum (400 MHz, DMSO-d_6) of compound* ***Z-2***.

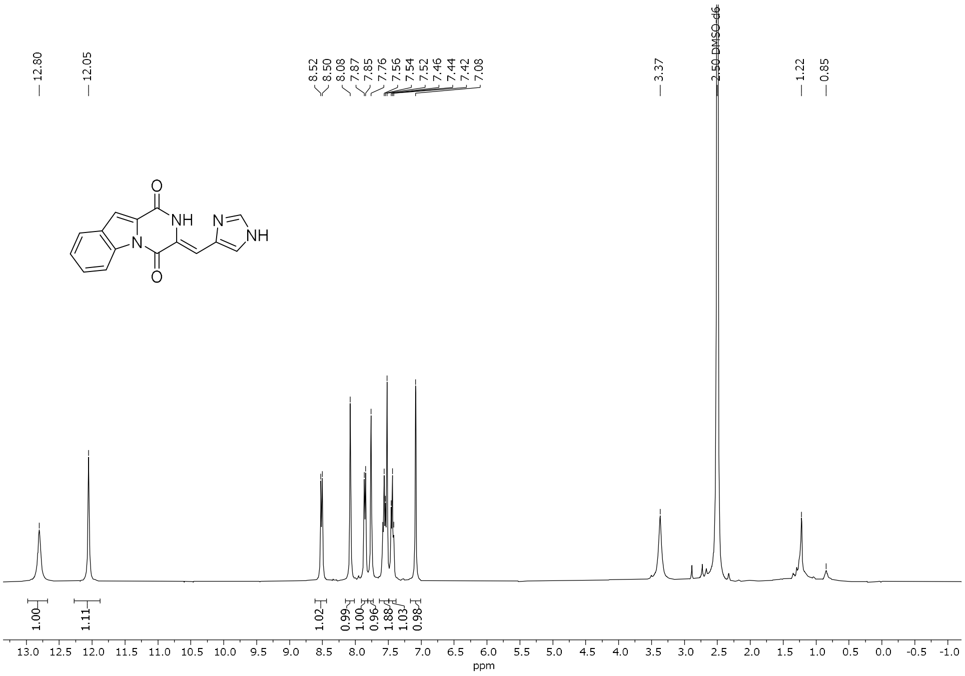

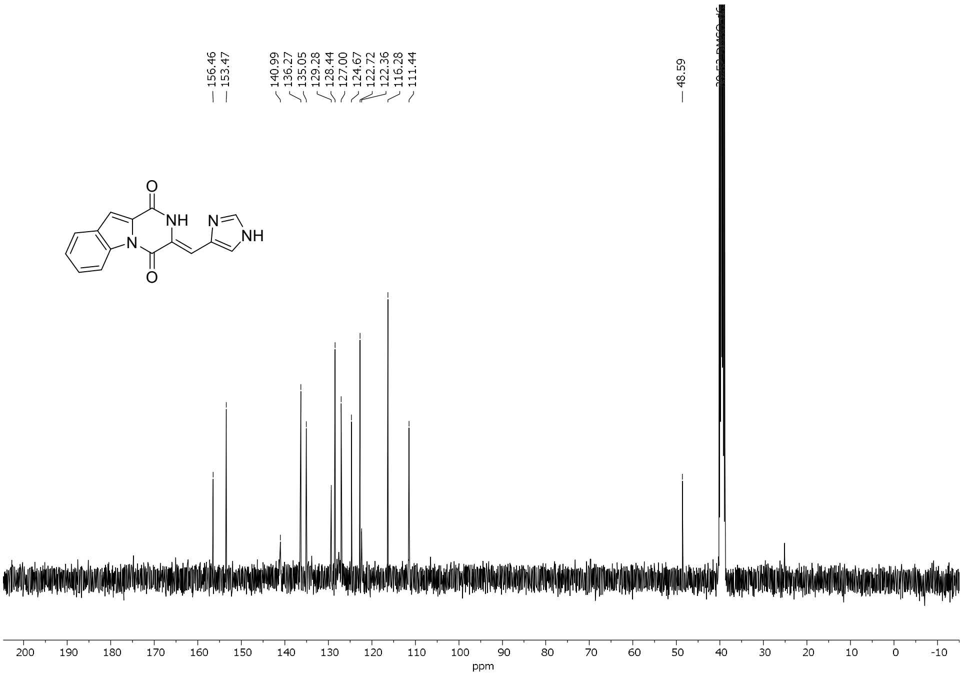

Figure S40: *^{1}H-NMR (400 MHz, top) and 13C-NMR (101 MHz, bottom) spectra in DMSO of compound **Z-3**.*

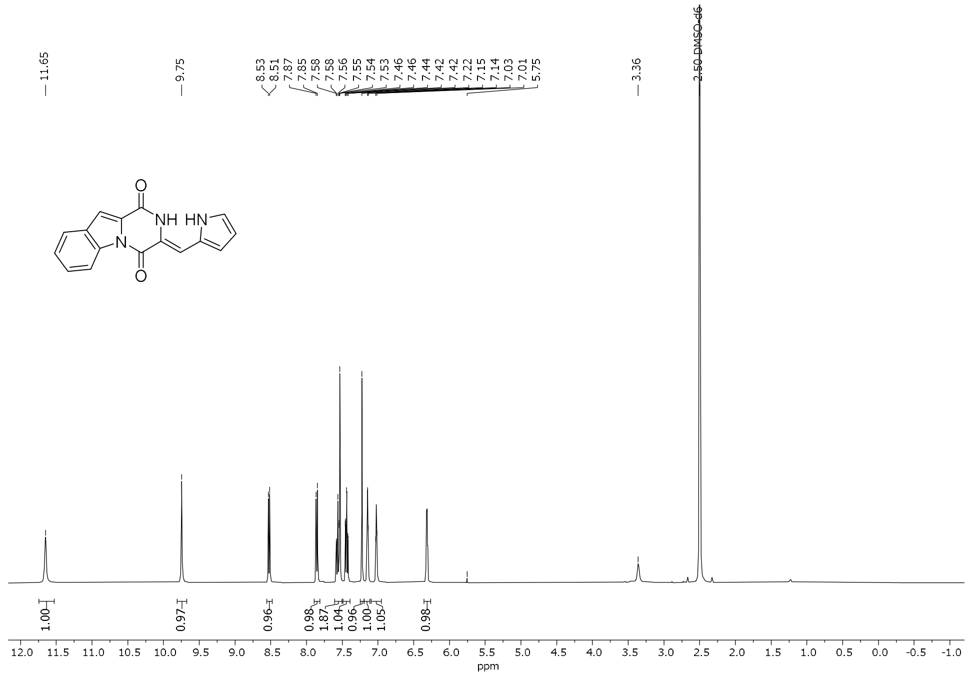

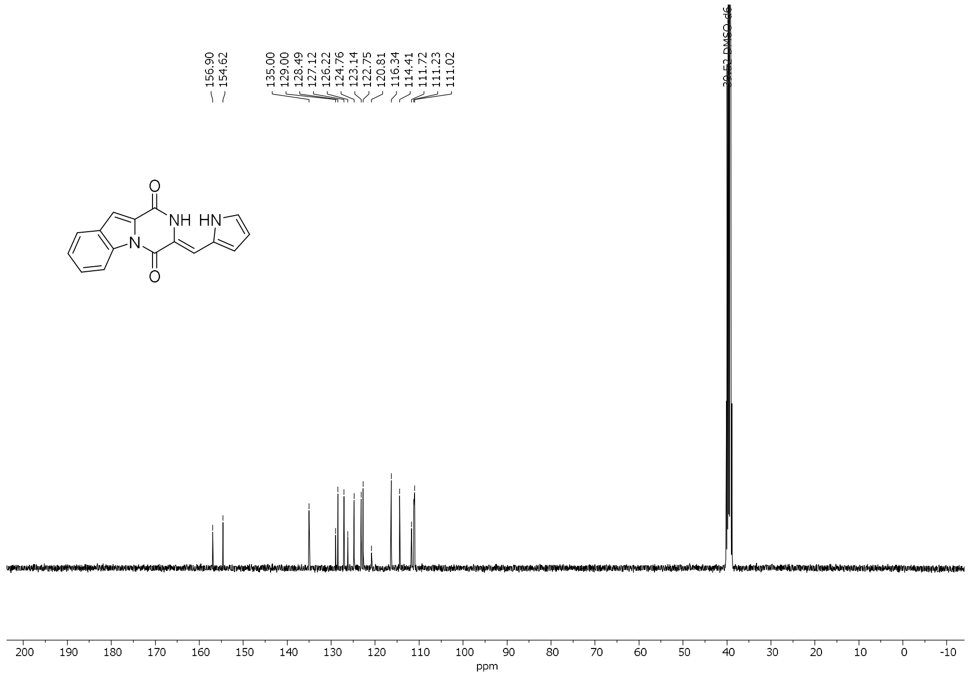

Figure S41: *^{1}H-NMR (400 MHz, top) and 13C-NMR (101 MHz, bottom) spectra in DMSO of compound* ***Z-4***.

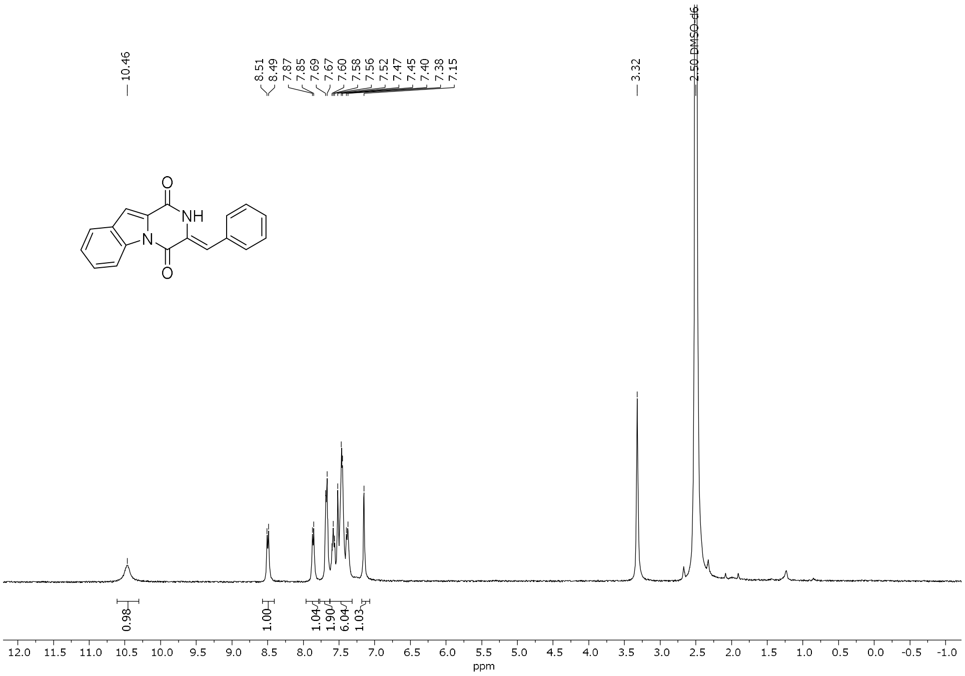

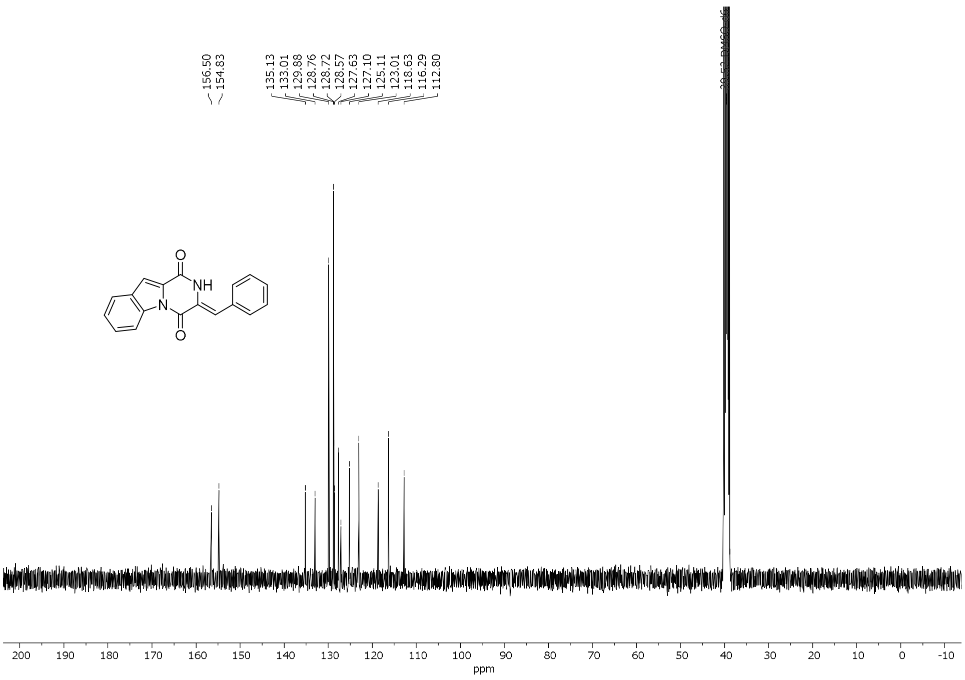

Figure S42: *^{1}H-NMR (400 MHz, top) and 13C-NMR (101 MHz, bottom) spectra in DMSO of compound* ***Z-6***.

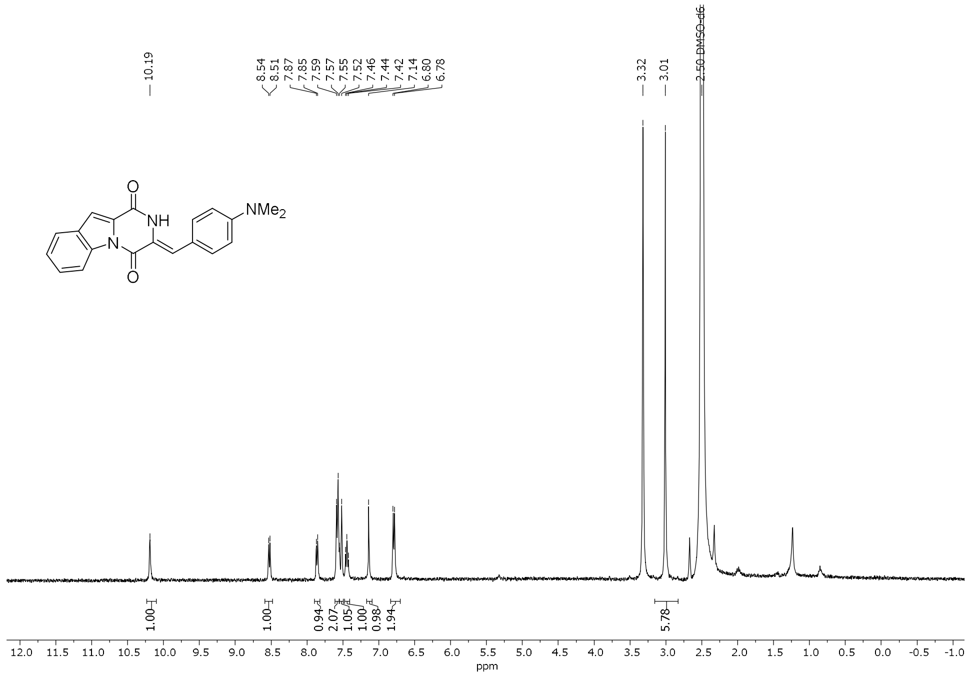

Figure S43: *^{1}H-NMR spectrum of compound* ***Z-5*** *(top) and ^{1}H-NMR spectrum of compound* ***Z-6*** *(bottom), (400 MHz, DMSO-d₆).*

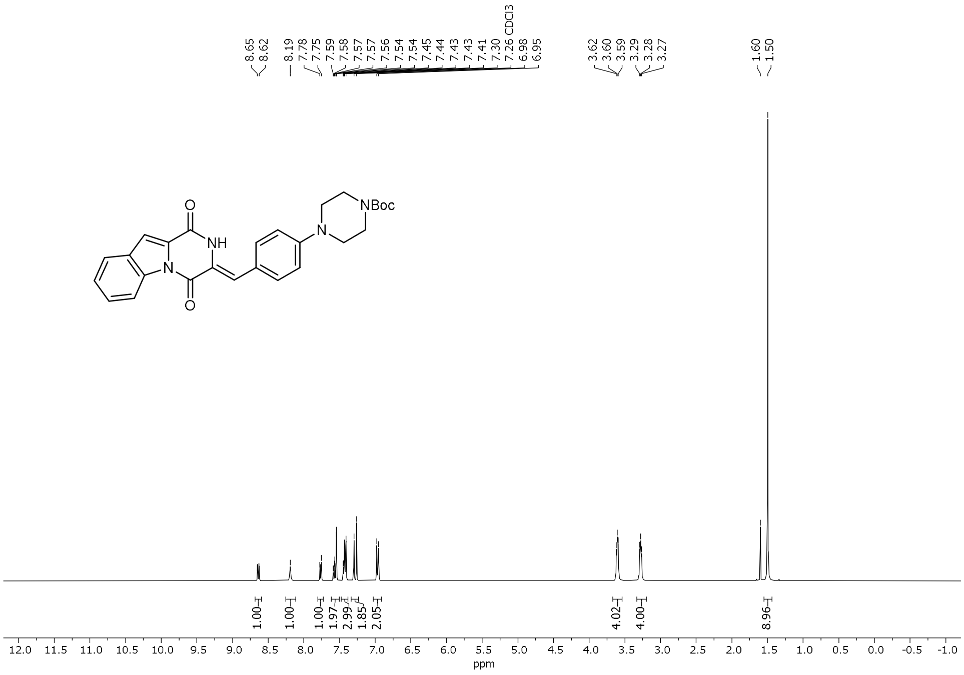

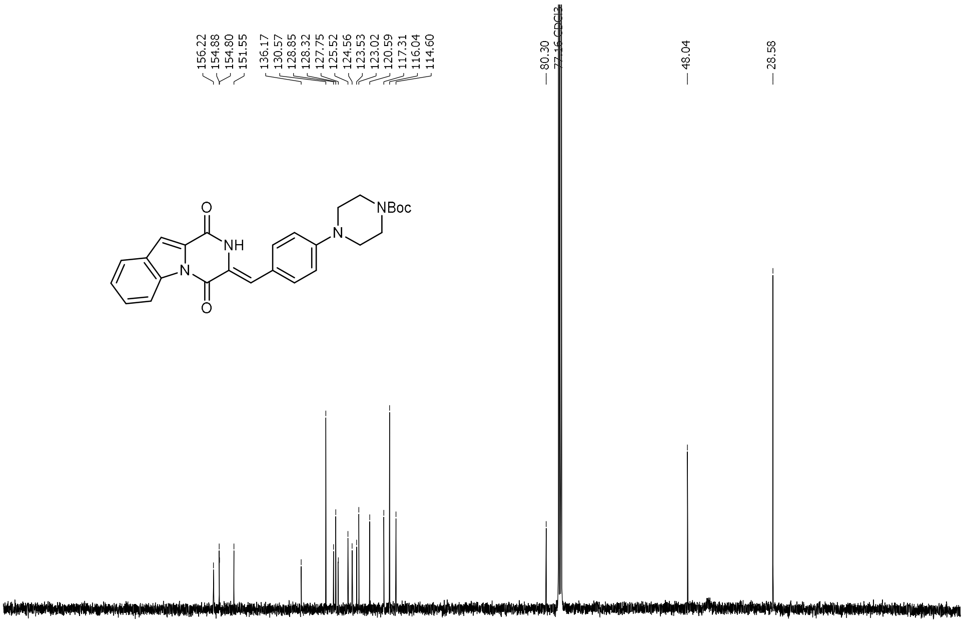

Figure S44: ^{1}H-NMR (top, 400 MHz) and ^{13}C-NMR (bottom, 101 MHz) spectra in DMSO-d_6 of compound **Boc-Z-7**.

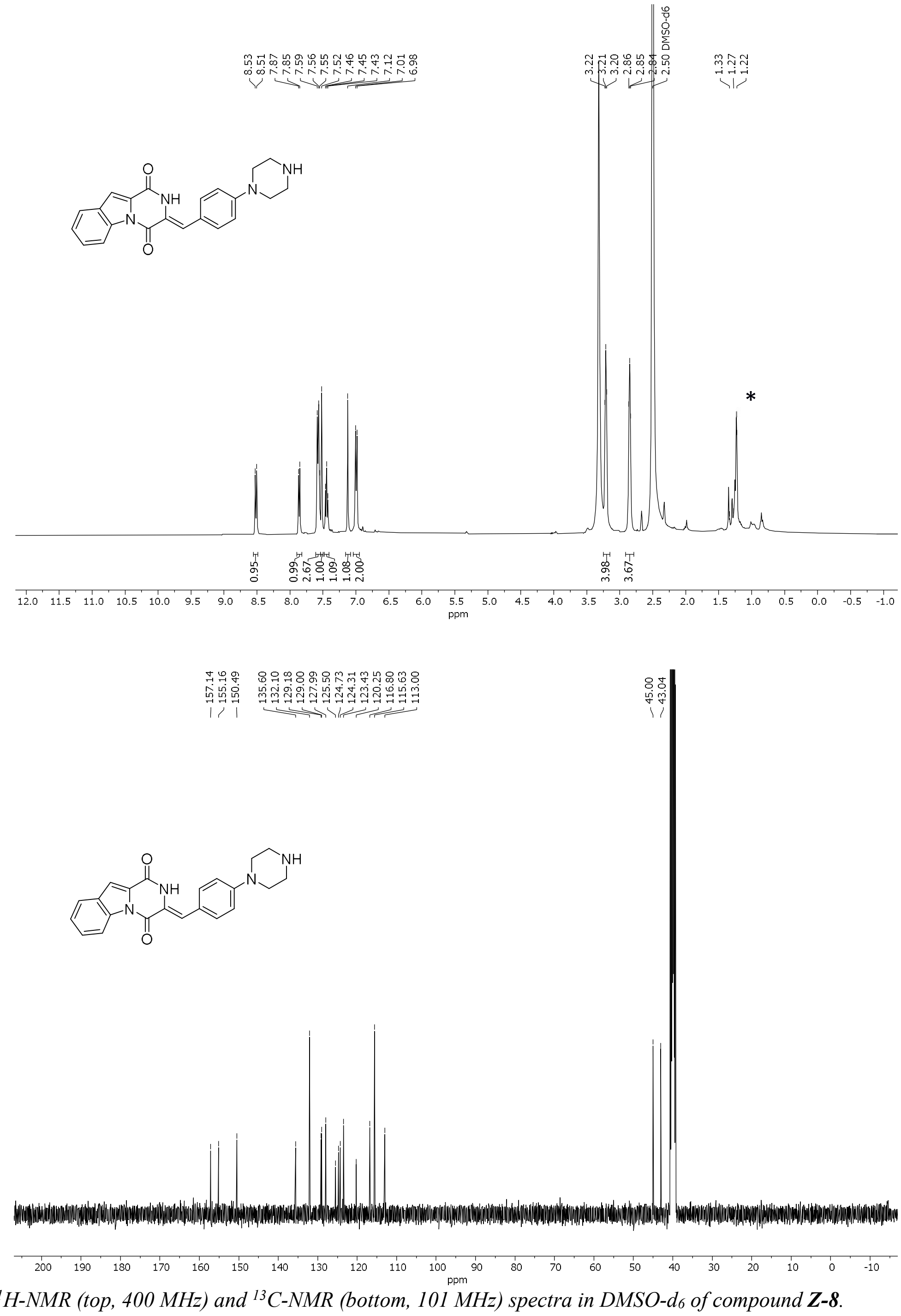

Figure S45: *^{1}H-NMR (top, 400 MHz) and ^{13}C-NMR (bottom, 101 MHz) spectra in DMSO-d$_6$ of compound **Z-8**.*

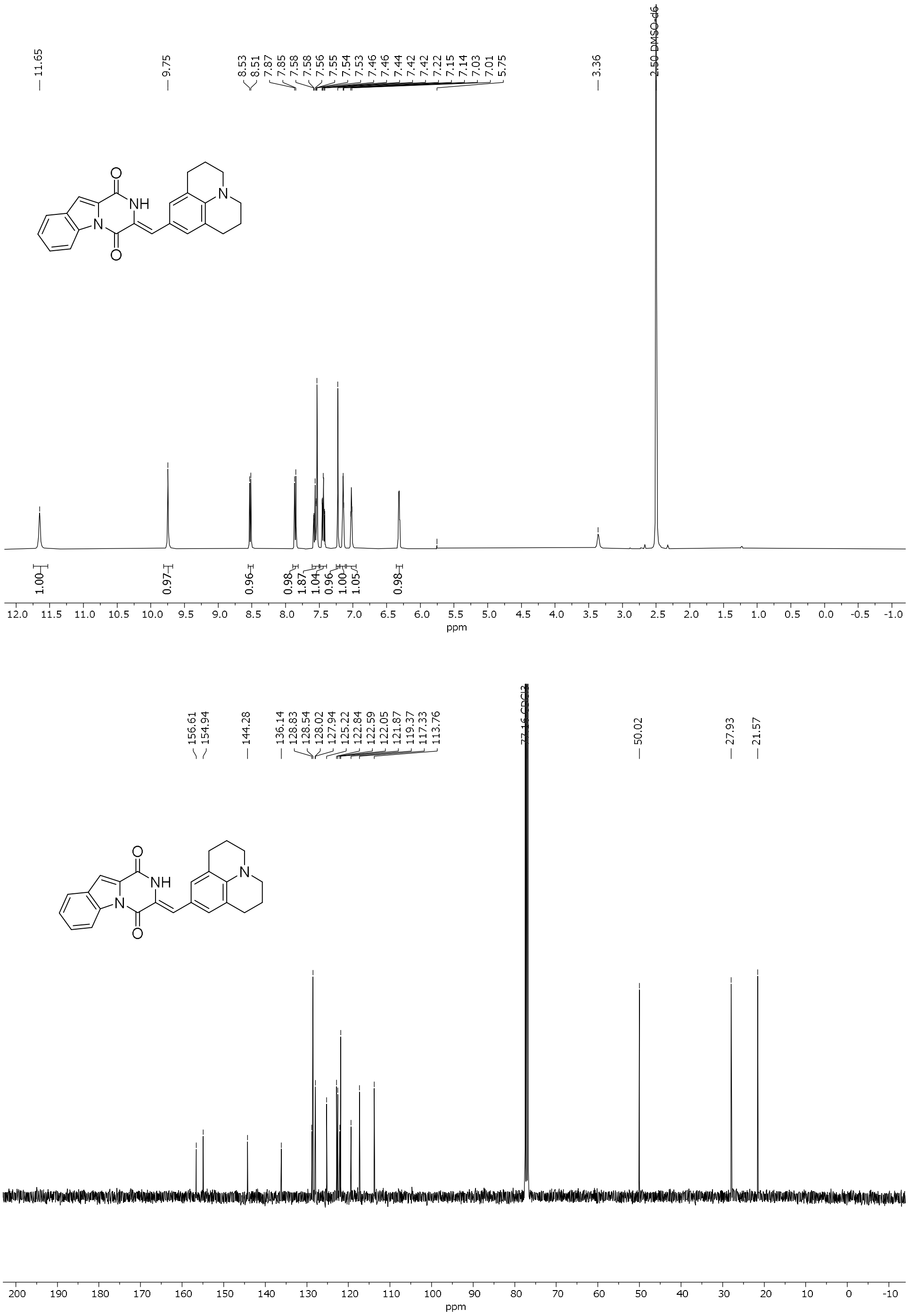

Figure S46: *^{1}H-NMR (400 MHz, top) and 13C-NMR (101 MHz, bottom) spectra in DMSO of compound* ***Z-8***.

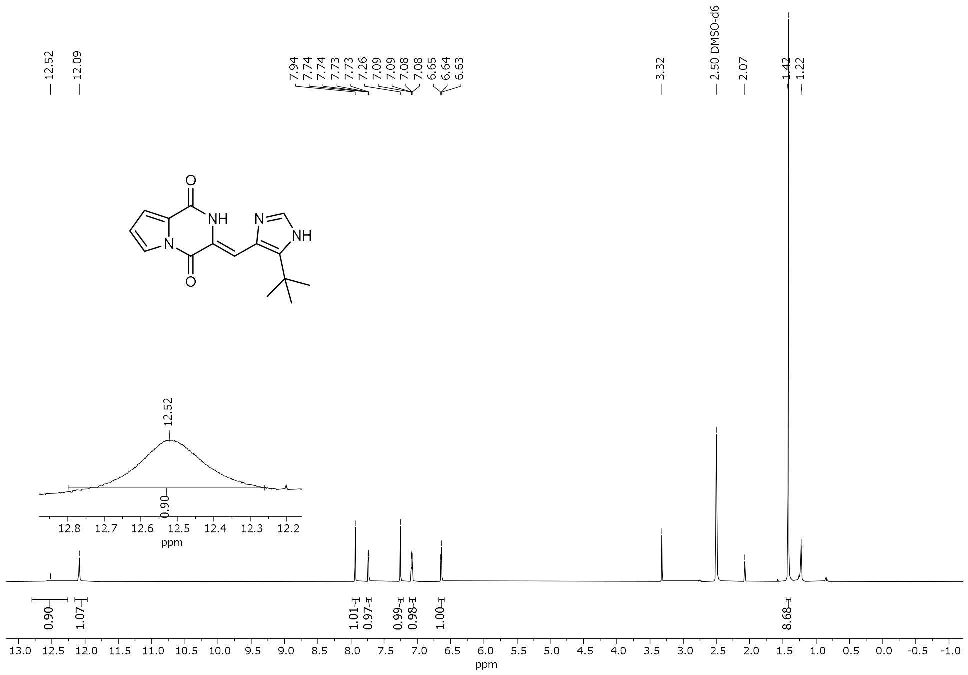

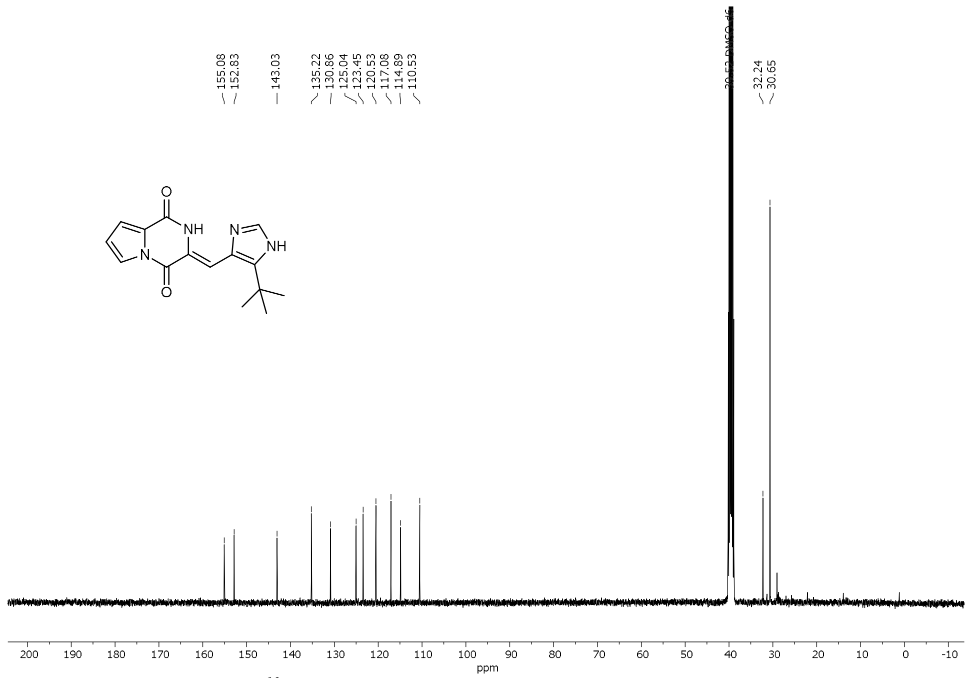

Figure S47: *^{1}H-NMR (top, 400 MHz) and ^{13}C-NMR (bottom, 101 MHz) spectra in DMSO-d₆ of compound **Z-10**.*

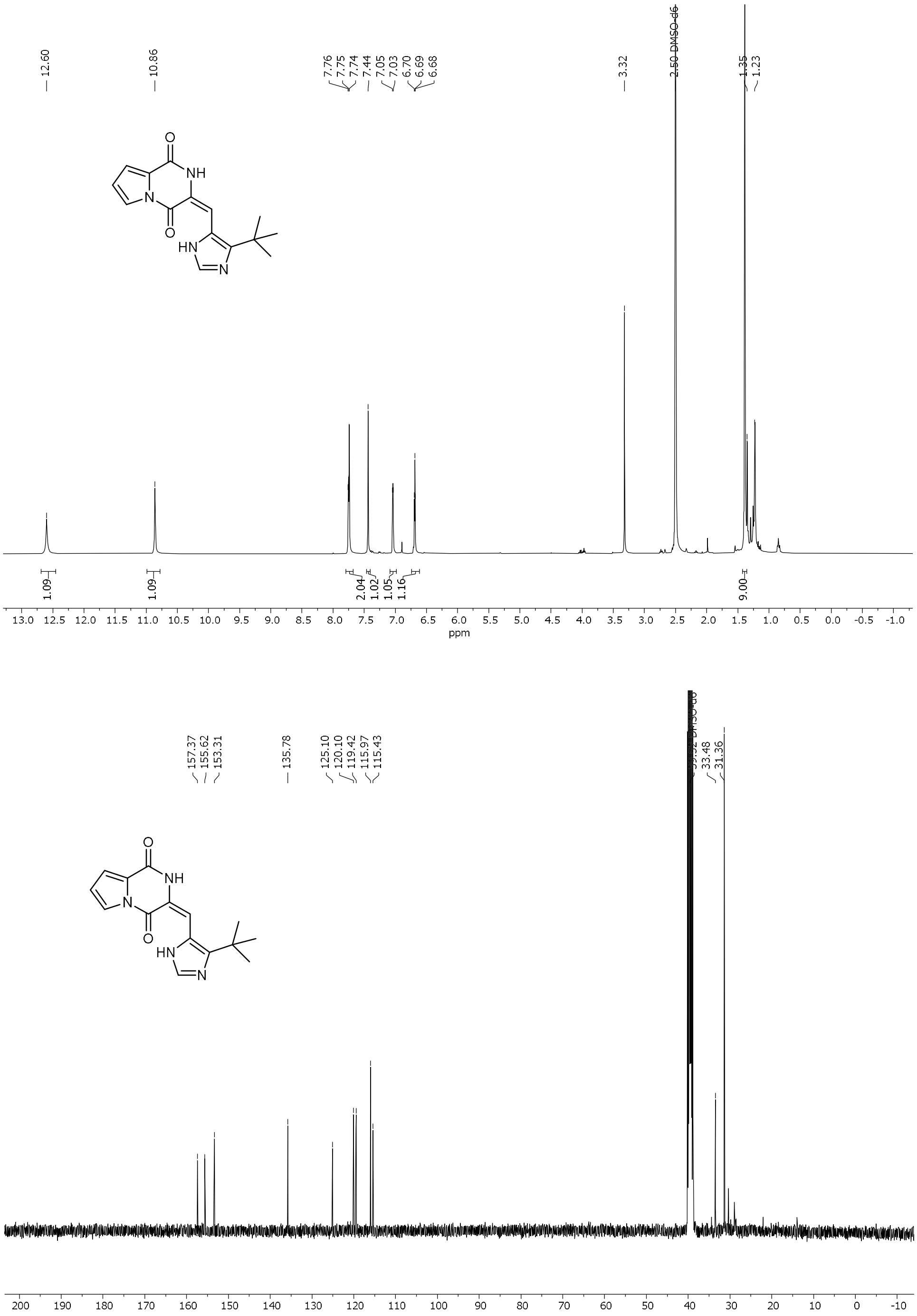

Figure S48: *^{1}H-NMR (top, 400 MHz) and ^{13}C-NMR (bottom, 101 MHz) spectra in DMSO-d_6 of compound* ***E-10***.

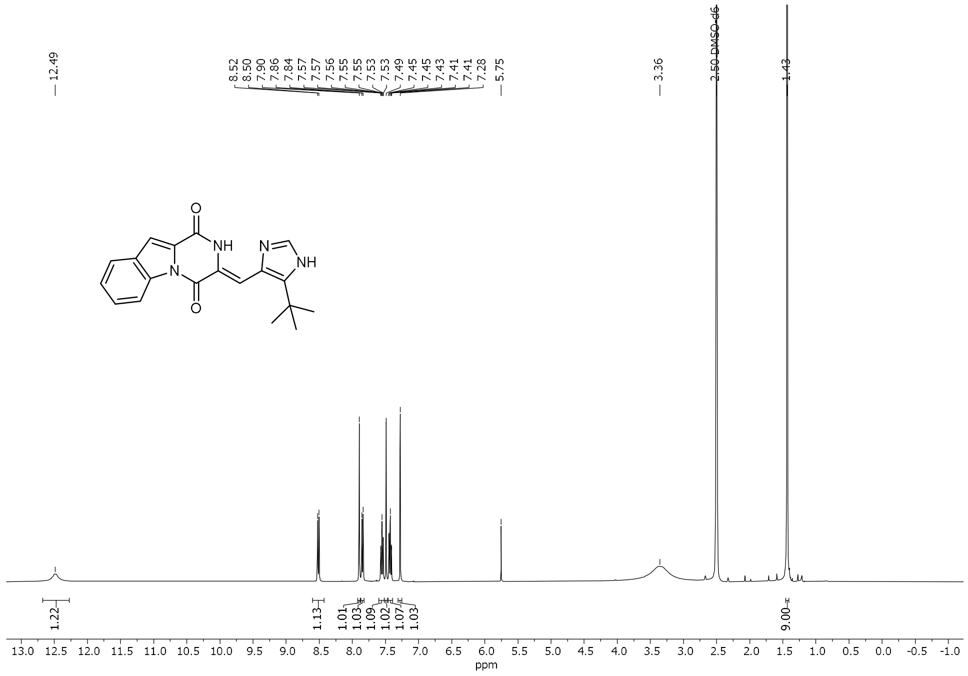

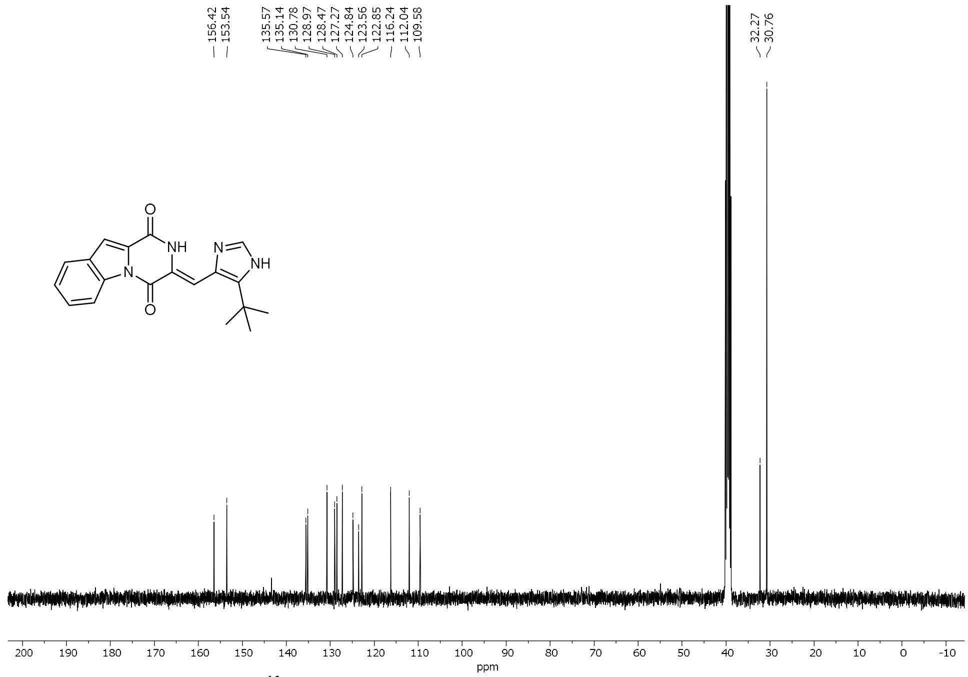

Figure S49: *^{1}H-NMR (top, 400 MHz) and ^{13}C-NMR (bottom, 101 MHz) spectra in DMSO-d₆ of compound* ***Z-11***.

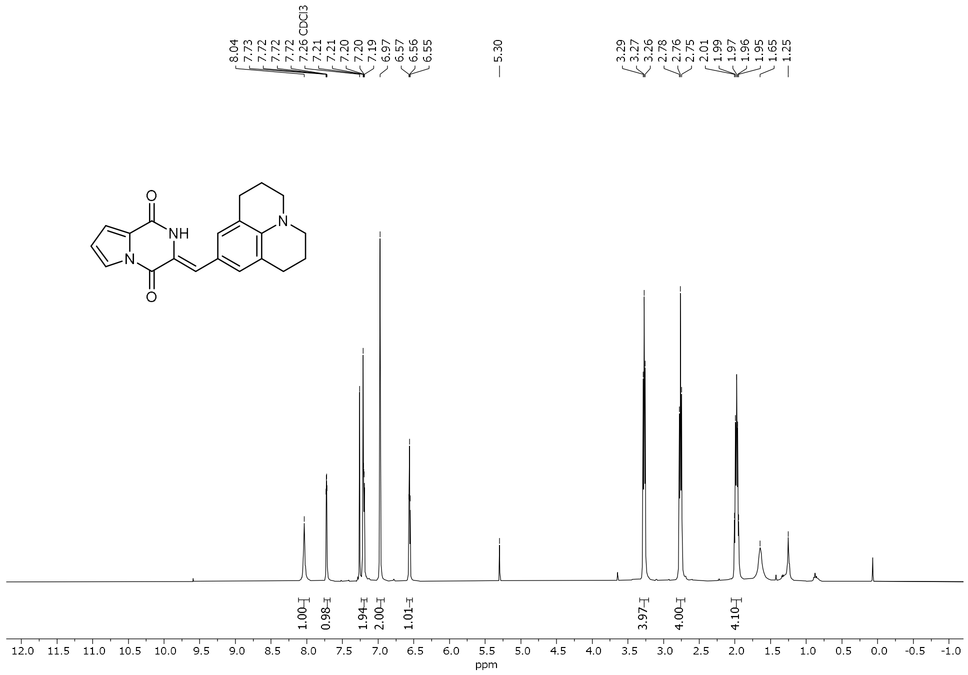

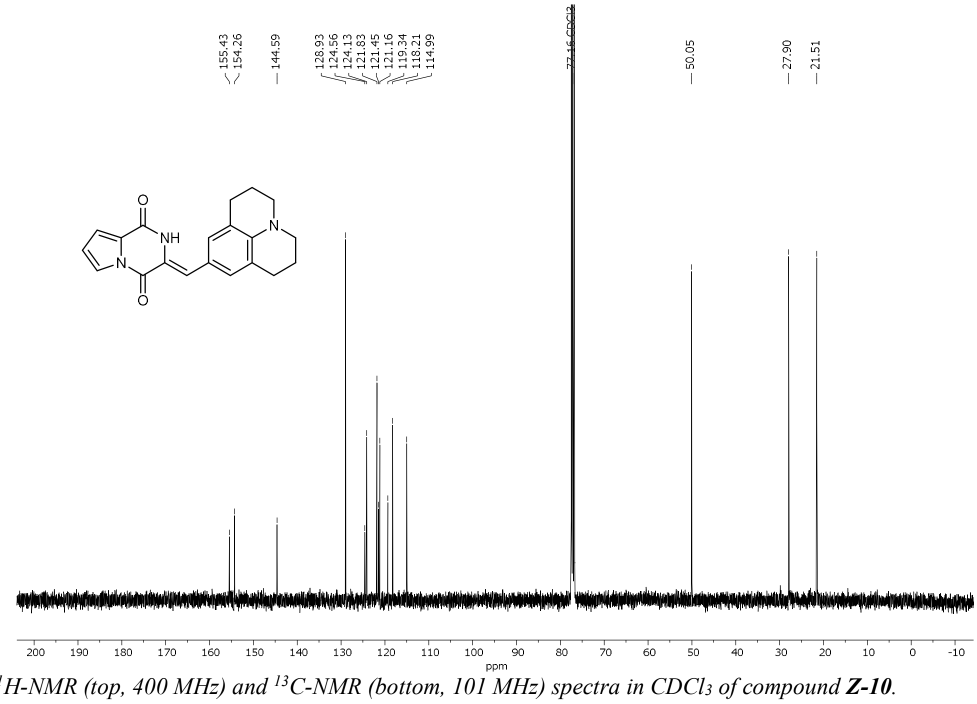

Figure S50: *^{1}H-NMR (top, 400 MHz) and ^{13}C-NMR (bottom, 101 MHz) spectra in $CDCl_3$ of compound* ***Z-10***.

Crystal Structure Determinations

A suitable crystal of each presented compound was selected and measured on a Stoe StadiVari diffractometer using Ga Kα (λ = 1.34143) radiation generated by an Excillium Metaljet X-ray source. The crystal was kept at 180 K during data collection. Using Olex2[5], the structure was solved with the SHELXT[6] structure solution program using Intrinsic Phasing and refined with the SHELXL[7] refinement package using Least Squares minimisation. Non-hydrogen atoms were refined with anisotropic displacement parameters; hydrogen atoms were modelled on idealized positions.

Crystallographic data for the compounds reported in this paper have been deposited with the Cambridge Crystallographic Data Centre as supplementary information no. CCDC-2446269 and 2446270. Copies of the data can be obtained free of charge from https://www.ccdc.cam.ac.uk/structures/.

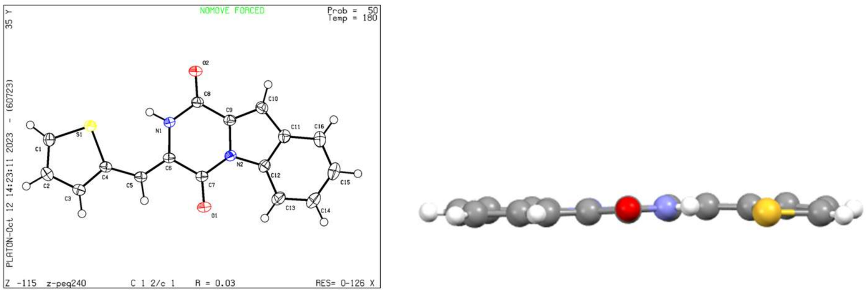

***Figure S51**: Structure refinement for **Z-2** (Z-PEG240), frontal view (left) and top view, visualized with Mercury v3.8 (right).*

***Table S5**: Crystal data of **Z-2**.*

Identification code	Z-PEG240
Empirical formula	$C_{16}H_{10}N_2O_2S$
Formula weight	294.32
Temperature/K	180
Crystal system	monoclinic
Space group	C2/c
a/Å	31.4068(14)
b/Å	5.2108(2)
c/Å	20.0470(10)
α/°	90
β/°	128.723(3)
γ/°	90
Volume/Å^3	2559.6(2)
Z	8
ρ_{calc}g/cm^3	1.528
μ/mm^{-1}	1.494
F(000)	1216.0
Crystal size/mm^3	0.14 × 0.05 × 0.04
Radiation	Ga Kα (λ = 1.34143)
2Θ range for data collection/°	7.674 to 125.002
Index ranges	$-41 \leq h \leq 39$, $-6 \leq k \leq 2$, $-26 \leq l \leq 26$
Reflections collected	15445
Independent reflections	3074 [R_{int} = 0.0147, R_{sigma} = 0.0101]
Data/restraints/parameters	3074/0/190
Goodness-of-fit on F^2	1.083
Final R indexes [I>=2σ (I)]	R_1 = 0.0310, wR_2 = 0.0865

Final R indexes [all data]	$R_1 = 0.0335$, $wR_2 = 0.0879$
Largest diff. peak/hole / e Å^{-3}	0.32/-0.34
CCDC-Number	2446269

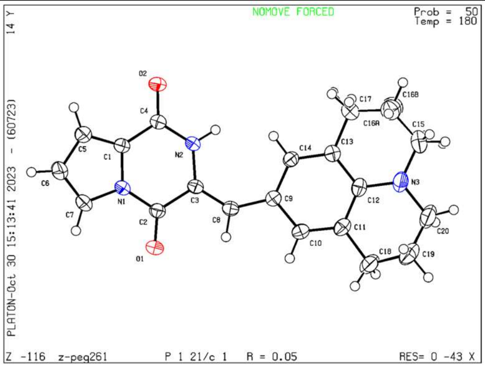

***Figure S52**: Structure refinement for **Z-10** (Z-PEG261).*

***Table S6**: Crystal data and structure refinement for **Z- 1** (Z-PEG261).*

Identification code	Z-PEG261
Empirical formula	$C_{20}H_{19}N_3O_2$
Formula weight	333.38
Temperature/K	180
Crystal system	monoclinic
Space group	$P2_1/c$
a/Å	8.1305(2)
b/Å	15.1242(5)
c/Å	14.0405(3)
α/°	90
β/°	106.456(2)
γ/°	90
Volume/Å^3	1655.80(8)
Z	4
ρ_{calc}g/cm^3	1.337
μ/mm^{-1}	0.456
F(000)	704.0
Crystal size/mm^3	0.16 × 0.05 × 0.04
Radiation	Ga Kα (λ = 1.34143)
2Θ range for data collection/°	7.648 to 124.994
Index ranges	$-3 \le h \le 10$, $-19 \le k \le 19$, $-18 \le l \le 17$
Reflections collected	22949
Independent reflections	3979 [$R_{int} = 0.0159$, $R_{sigma} = 0.0103$]
Data/restraints/parameters	3979/0/225
Goodness-of-fit on F^2	1.077
Final R indexes [I>=2σ (I)]	$R_1 = 0.0450$, $wR_2 = 0.1225$
Final R indexes [all data]	$R_1 = 0.0500$, $wR_2 = 0.1260$
Largest diff. peak/hole / e Å^{-3}	0.46/-0.35
CCDC-Number	2446270

Theoretically Obtained Geometries

The following calculations were performed with the GAUSSIAN09 program package, employing the B3LYP-GD3BJ/6-311G(d,p) PCM(DMSO) level of theory, with the RMS force criterion set to $1 \cdot 10^{-6}$ Hartrees (opt=verytight). (These parameters were successfully applied for calculations in another arylidene-substituted heterocyclic photochromic system – hemiindigos[8], as well as previously in carbo- and heterocyclic HPIs[2, 9]). The global minima geometries were first optimized on the MPW1K/6-311G(d,p) and subsequently on the B3LYP-GD3BJ/6-311G(d,p). Frequency analysis confirmed all structures to be minimum structures since no imaginary frequencies have been found. Visualization of minimized structures, molecular orbitals, and calculated extinction spectra were done with GaussView 6.1.1.

The presented data were obtained and visualized with Avogadro 1.2.0.

Cartesian Coordinates of the optimized structures:

***Z*-1**

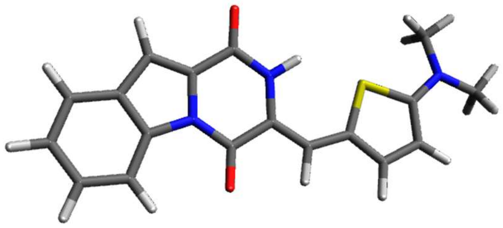

C	6.01467	-1.51182	0.17106
C	6.69183	-0.27864	0.18355
C	5.99498	0.91803	0.12277
C	4.59504	0.87842	0.04850
C	3.93017	-0.37701	0.03731
C	4.62850	-1.58373	0.09815
C	3.59167	1.90104	-0.02606
C	2.37766	1.27904	-0.07682
N	2.55755	-0.11656	-0.03990
C	1.04079	1.85290	-0.18407
N	0.03167	0.92683	-0.19535
C	0.16804	-0.47030	-0.12027
C	1.51636	-1.05063	-0.06350
O	1.74885	-2.25197	-0.02414
O	0.82549	3.05863	-0.26971
C	-0.88736	-1.33814	-0.09088

S	-3.09931	0.43788	0.08628
C	-2.28601	-1.13276	-0.07301
C	-4.68815	-0.31110	-0.01984
C	-4.57320	-1.69797	-0.12407
C	-3.24386	-2.13510	-0.15690
N	-5.81077	0.43968	0.00680
C	-5.74422	1.85400	0.35041
C	-7.09742	-0.24133	0.10904
H	6.58669	-2.43072	0.21923
H	7.77366	-0.26855	0.24124
H	6.51556	1.86812	0.13206
H	4.10530	-2.52667	0.08781
H	3.74380	2.96810	-0.04356
H	-0.88648	1.32341	-0.34492
H	-0.58892	-2.38045	-0.08484
H	-5.42511	-2.35813	-0.17212
H	-2.96194	-3.17661	-0.24526
H	-6.68506	2.32872	0.07932
H	-5.56351	2.00696	1.42125
H	-4.94567	2.33979	-0.21420
H	-7.18166	-1.00354	-0.66712
H	-7.22804	-0.71710	1.08795
H	-7.89050	0.48824	-0.03960

***E*-1**

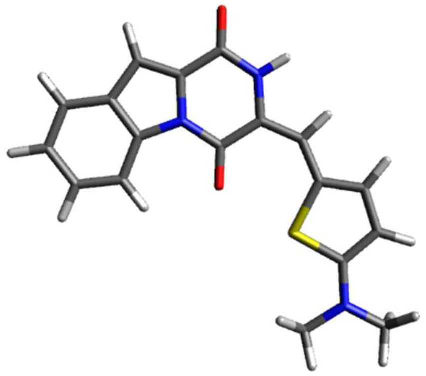

C	3.53952	-3.33234	-0.01388
C	4.90418	-2.99020	-0.01078
C	5.30658	-1.66379	-0.00358
C	4.32509	-0.66187	0.00069
C	2.95197	-1.02810	-0.00254
C	2.54353	-2.36294	-0.00987
C	4.39212	0.77109	0.00826
C	3.10987	1.23932	0.00915
N	2.20936	0.15793	0.00246
C	2.61397	2.61171	0.01713
N	1.25365	2.70742	0.01536
C	0.31532	1.64745	0.00521
C	0.81537	0.28557	0.00191
O	0.09938	-0.71219	-0.00082
O	3.34952	3.59797	0.02493
C	-1.01729	1.98559	-0.00078
S	-2.56417	-0.45463	-0.02513
C	-2.24887	1.29636	-0.01443
C	-4.30202	-0.17973	-0.05090
C	-4.60851	1.18549	-0.02932
C	-3.46592	1.98217	-0.01742
N	-5.16674	-1.21555	-0.09531
C	-4.69131	-2.58056	0.08853
C	-6.59341	-0.94722	0.05072
H	3.25525	-4.37787	-0.01951
H	5.64827	-3.77775	-0.01412
H	6.35739	-1.39986	-0.00124
H	1.49661	-2.62127	-0.01220
H	5.27765	1.38573	0.01284

H	0.89372	3.65304	0.02023
H	-1.18855	3.06044	0.00547
H	-5.61530	1.57349	-0.02018
H	-3.49739	3.06452	-0.00723
H	-4.42569	-2.78349	1.13305
H	-5.47280	-3.27282	-0.21816
H	-3.81437	-2.76030	-0.53685
H	-6.90855	-0.18313	-0.66184
H	-7.14493	-1.86076	-0.16062
H	-6.84086	-0.60843	1.06341

***Z*-2**

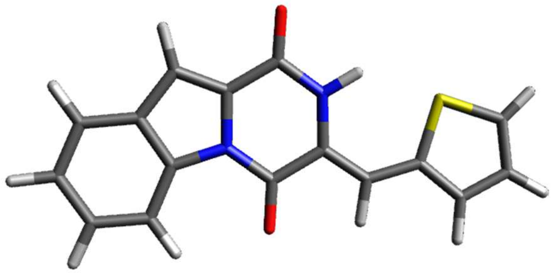

C	4.90603	-1.76524	0.07571
C	5.67054	-0.58546	0.09465
C	5.06127	0.66005	0.07047
C	3.66190	0.72118	0.02676
C	2.90906	-0.48149	0.00817
C	3.51625	-1.73607	0.03223
C	2.73310	1.81609	-0.00732
C	1.47713	1.28742	-0.04220
N	1.55521	-0.11972	-0.03402
N	-0.89703	1.11020	-0.11556
C	-0.86519	-0.28625	-0.07297
C	0.45548	-0.97001	-0.05546
O	0.58460	-2.18204	-0.04694
C	-1.96473	-1.08069	-0.04316
C	-3.36019	-0.76062	-0.00989
C	0.18482	1.95985	-0.10802
O	0.05393	3.17549	-0.16284
C	-4.38170	-1.68372	-0.14881

C	-5.67717	-1.11729	-0.07912
C	-5.65596	0.23661	0.11969
S	-4.04044	0.84230	0.23667
H	5.41047	-2.72377	0.09525
H	6.75106	-0.65457	0.12840
H	5.64909	1.56974	0.08475
H	2.92710	-2.63924	0.01716
H	2.96375	2.86916	-0.00892
H	-1.78570	1.57993	-0.23046
H	-1.74360	-2.14112	-0.05805
H	-4.18670	-2.73657	-0.30238
H	-6.59193	-1.68617	-0.17066
H	-6.48574	0.91864	0.21900

E-*2*

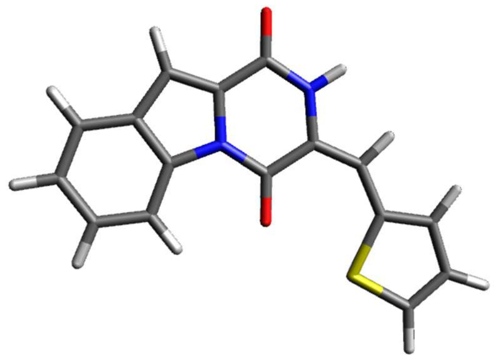

C	-3.44876	-2.84908	-0.00002
C	-4.68956	-2.18824	-0.00003
C	-4.76025	-0.80319	-0.00003
C	-3.56591	-0.07025	-0.00003
C	-2.32365	-0.75671	-0.00002
C	-2.24708	-2.14889	-0.00001
C	-3.28490	1.33839	-0.00003
C	-1.93017	1.48551	-0.00003
N	-1.31521	0.21786	-0.00001
N	0.22996	2.46728	-0.00001
C	0.88673	1.22151	-0.00000
C	0.05969	0.00630	-0.00000
O	0.52513	-1.12319	-0.00000
C	2.24953	1.22249	0.00001
C	3.28765	0.23562	0.00003
C	-1.11935	2.69826	-0.00003

O	-1.59188	3.82982	-0.00003
C	4.62520	0.61664	0.00004
C	5.53443	-0.46133	0.00006
C	4.89451	-1.67387	0.00005
S	3.17520	-1.52231	0.00004
H	-3.42506	-3.93216	-0.00001
H	-5.60153	-2.77292	-0.00003
H	-5.71568	-0.29273	-0.00004
H	-1.29422	-2.65365	-0.00000
H	-3.99714	2.14759	-0.00004
H	0.80357	3.30125	-0.00001
H	2.68031	2.22118	0.00001
H	4.92097	1.65755	0.00004
H	6.60976	-0.34883	0.00007
H	5.33799	-2.65839	0.00006

***Z*-3**

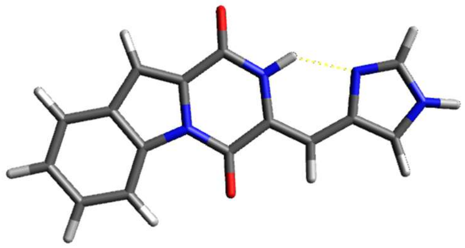

C	-4.68874	-1.68833	0.00000
C	-5.42112	-0.48836	0.00000
C	-4.77776	0.74055	0.00000
C	-3.37672	0.76483	-0.00000
C	-2.65653	-0.45788	-0.00000
C	-3.29785	-1.69568	0.00000
C	-2.41672	1.83402	-0.00000
C	-1.17510	1.27217	-0.00000
N	-1.29314	-0.13309	-0.00000
C	0.14077	1.91293	-0.00000
N	1.19242	1.04168	-0.00000
C	1.11417	-0.35366	-0.00000
C	-0.21338	-1.01177	-0.00000

O	-0.37029	-2.22140	-0.00000
O	0.29006	3.13116	-0.00001
C	2.22062	-1.13768	-0.00000
N	3.94211	0.66575	0.00000
C	3.58076	-0.68029	0.00000
C	4.72745	-1.45629	0.00000
N	5.77019	-0.57243	0.00001
C	5.25458	0.69025	0.00001
H	-5.21906	-2.63305	0.00001
H	-6.50374	-0.52803	0.00000
H	-5.34166	1.66553	0.00000
H	-2.73216	-2.61398	0.00000
H	-2.61813	2.89306	-0.00000
H	2.14058	1.42671	-0.00000
H	2.05039	-2.20585	-0.00000
H	4.87123	-2.52300	0.00000
H	6.75061	-0.81290	0.00001
H	5.87619	1.57099	0.00001

***E*-3**

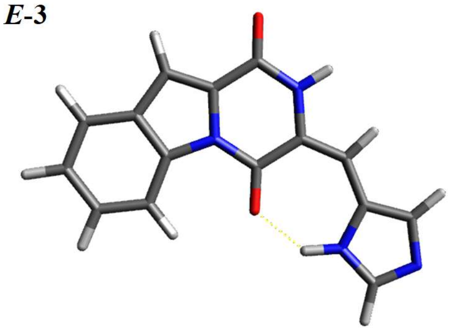

C	-3.37131	-2.76583	-0.00003
C	-4.57571	-2.04179	-0.00001
C	-4.57346	-0.65481	0.00002
C	-3.34259	0.01407	0.00003
C	-2.13736	-0.73504	0.00000
C	-2.13441	-2.12922	-0.00003
C	-2.99088	1.40683	0.00005
C	-1.63145	1.48618	0.00004
N	-1.07909	0.18876	0.00001
C	-0.76208	2.65662	0.00006

N	0.57200	2.35632	0.00005
C	1.16997	1.07911	0.00002
C	0.27996	-0.09420	-0.00000
O	0.66428	-1.25982	-0.00003
O	-1.17758	3.81056	0.00009
C	2.53461	1.02866	0.00000
N	3.32047	-1.38569	-0.00005
C	3.50275	-0.01556	-0.00003
C	4.53818	-1.96477	-0.00007
N	5.51789	-1.06379	-0.00007
C	4.89008	0.14361	-0.00004
H	-3.40359	-3.84868	-0.00006
H	-5.51706	-2.57783	-0.00002
H	-5.50054	-0.09459	0.00004
H	-1.21054	-2.68469	-0.00005
H	-3.66133	2.25096	0.00007
H	1.18756	3.15962	0.00006
H	3.01620	2.00277	0.00002
H	2.39522	-1.80663	-0.00005
H	4.66952	-3.03537	-0.00010
H	5.43419	1.07628	-0.00002

***Z*-4**

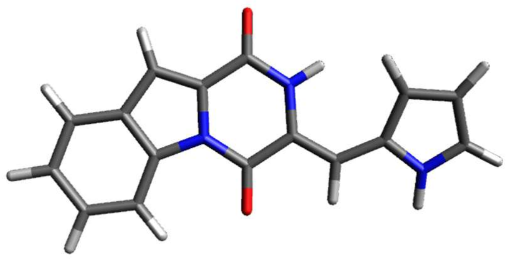

C	-4.62163	-1.81005	0.07885
C	-5.40902	-0.64510	0.10192
C	-4.82413	0.61188	0.07859
C	-3.42604	0.70109	0.03150
C	-2.64957	-0.48702	0.00904
C	-3.23308	-1.75342	0.03217
C	-2.51870	1.81341	-0.00290

C	-1.25250	1.30856	-0.04058
N	-1.30407	-0.09982	-0.03492
C	0.02724	2.00706	-0.11735
N	1.12075	1.17889	-0.11421
C	1.11169	-0.21953	-0.06507
C	-0.18473	-0.93018	-0.05553
O	-0.29242	-2.14614	-0.04787
O	0.12981	3.22611	-0.19041
C	2.24403	-0.96859	-0.02948
C	3.60087	-0.52955	0.04088
C	4.21473	0.68752	0.36813
C	5.61128	0.50296	0.29809
C	5.83745	-0.81399	-0.06996
N	4.62991	-1.42291	-0.21042
H	-5.10736	-2.77827	0.09762
H	-6.48797	-0.73523	0.13815
H	-5.42977	1.50984	0.09603
H	-2.62592	-2.64455	0.01386
H	-2.76909	2.86194	-0.00342
H	2.00736	1.64236	-0.25896
H	2.07478	-2.03804	-0.07008
H	3.72213	1.58984	0.69245
H	6.37056	1.24033	0.50383
H	6.75628	-1.35068	-0.23904

***E*-4**

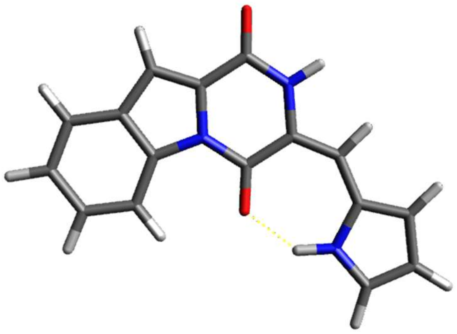

C	3.37989	-2.76641	-0.00006
C	4.58461	-2.04192	-0.00005
C	4.58200	-0.65530	-0.00001
C	3.35095	0.01447	0.00001
C	2.14500	-0.73499	-0.00001
C	2.14342	-2.12996	-0.00005
C	2.99913	1.40638	0.00004
C	1.63863	1.48421	0.00004
N	1.08744	0.18741	0.00001
C	0.76785	2.65382	0.00007
N	-0.56329	2.35114	0.00006
C	-1.16118	1.06970	0.00003
C	-0.27558	-0.09652	0.00000
O	-0.65362	-1.26686	-0.00003
O	1.18412	3.80929	0.00010
C	-2.53137	1.02588	0.00002
C	-3.51080	-0.00151	-0.00001
C	-4.90267	0.20411	-0.00002
C	-5.52395	-1.05423	-0.00005
C	-4.50958	-2.00921	-0.00006
N	-3.31660	-1.36890	-0.00004
H	3.41241	-3.84931	-0.00009
H	5.52610	-2.57788	-0.00006
H	5.50922	-0.09512	0.00000
H	1.21929	-2.68498	-0.00006
H	3.66879	2.25110	0.00006
H	-1.18076	3.15278	0.00008
H	-3.00063	2.00614	0.00004
H	-5.38233	1.17084	0.00000

H	-6.58257	-1.25961	-0.00006
H	-4.56294	-3.08591	-0.00008

***Z*-5**

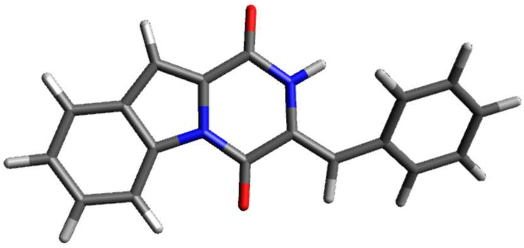

C	-4.90229	-1.77024	0.14699
C	-5.67425	-0.59680	0.09167
C	-5.07258	0.64985	0.00439
C	-3.67355	0.71794	-0.02788
C	-2.91322	-0.47837	0.02875
C	-3.51210	-1.73376	0.11660
C	-2.75084	1.81598	-0.11369
C	-1.49154	1.29596	-0.10534
N	-1.56069	-0.10987	-0.01901
N	0.88367	1.13561	-0.14030
C	0.86391	-0.25666	-0.01784
C	-0.45632	-0.95146	0.01524
O	-0.57352	-2.16131	0.08422
C	1.97242	-1.02713	0.04738
C	3.37236	-0.61768	0.07206
C	-0.20253	1.97409	-0.20058
O	-0.08321	3.18515	-0.33216
C	4.32674	-1.47649	-0.50290
C	5.67384	-1.13864	-0.51923
C	6.10368	0.05571	0.05938
C	5.17575	0.90151	0.66446
C	3.82469	0.57153	0.67290
H	-5.40071	-2.72971	0.21474
H	-6.75462	-0.67134	0.11762
H	-5.66629	1.55478	-0.03862
H	-2.91721	-2.63237	0.15809

H	-2.98843	2.86559	-0.17733
H	1.77841	1.58848	-0.27351
H	1.77744	-2.09246	0.03750
H	3.99683	-2.40841	-0.94745
H	6.39081	-1.80916	-0.97780
H	7.15476	0.31786	0.05123
H	5.50483	1.81690	1.14144
H	3.13212	1.21691	1.19850

***E*-5**

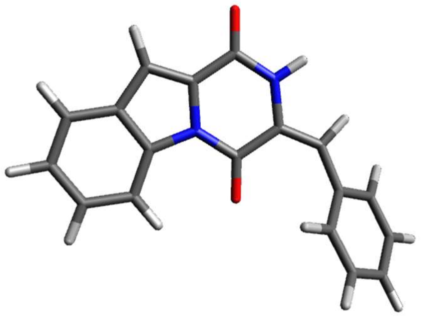

C	-3.44248	-2.90083	-0.18078
C	-4.68928	-2.25415	-0.13349
C	-4.77279	-0.87198	-0.05176
C	-3.58549	-0.12934	-0.01777
C	-2.33650	-0.80103	-0.06710
C	-2.24712	-2.18986	-0.14845
C	-3.31859	1.28007	0.06258
C	-1.96612	1.44096	0.06373
N	-1.33682	0.18321	-0.01279
N	0.18243	2.44587	0.08908
C	0.86455	1.21392	0.04820
C	0.04055	-0.02400	-0.03270
O	0.50177	-1.14430	-0.13778
C	2.21944	1.24724	-0.00690
C	3.25985	0.22035	0.01744
C	-1.16925	2.66094	0.11340
O	-1.65323	3.78546	0.17183
C	4.44965	0.50521	-0.67864
C	5.51384	-0.38856	-0.67916
C	5.42696	-1.57396	0.04857

C	4.26918	-1.85154	0.77618
C	3.19569	-0.96978	0.76116
H	-3.40806	-3.98173	-0.24484
H	-5.59563	-2.84678	-0.16150
H	-5.73272	-0.37151	-0.01444
H	-1.29005	-2.68475	-0.18686
H	-4.03894	2.08063	0.11126
H	0.74443	3.28664	0.13368
H	2.62908	2.25130	-0.09583
H	4.52929	1.43538	-1.23017
H	6.41297	-0.15475	-1.23675
H	6.25753	-2.26976	0.05948
H	4.20371	-2.76062	1.36254
H	2.30826	-1.19885	1.32991

***Z*-6**

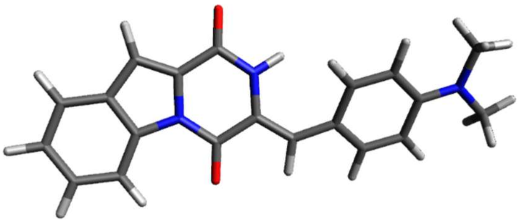

C	3.18659	-1.67359	-0.33779
C	2.23775	-0.70051	0.04295
C	2.75178	0.54212	0.47269
C	4.10644	0.81038	0.47943
C	5.04978	-0.15394	0.04094
C	4.54214	-1.41925	-0.35136
C	0.83976	-1.05866	0.00886
N	6.38693	0.11846	0.01280
C	7.33911	-0.92040	-0.35505
C	6.88662	1.39641	0.50108
C	-0.27165	-0.27993	-0.05982
N	-0.23847	1.11531	-0.20607
C	-1.31258	1.96386	-0.26313
C	-2.60671	1.30163	-0.13076

N	-2.68774	-0.10115	-0.02976
C	-1.58584	-0.95667	-0.00199
O	-1.72674	-2.16655	0.08412
O	-1.18525	3.17305	-0.42467
C	-3.86165	1.83553	-0.12249
C	-4.79201	0.74799	-0.00935
C	-4.04073	-0.45549	0.04555
C	-6.19153	0.69218	0.04883
C	-6.80305	-0.54728	0.15940
C	-6.04069	-1.72788	0.21269
C	-4.65161	-1.70446	0.15696
H	2.83556	-2.65397	-0.64101
H	2.09278	1.30028	0.87632
H	4.44062	1.77097	0.84305
H	5.21673	-2.20431	-0.66012
H	0.62226	-2.12043	0.00571
H	7.13446	-1.30800	-1.35740
H	8.34056	-0.49740	-0.35590
H	7.32007	-1.76054	0.34896
H	6.43715	2.23112	-0.04467
H	6.68312	1.53450	1.56946
H	7.96252	1.43612	0.35001
H	0.65477	1.55103	-0.38986
H	-4.08967	2.88676	-0.19351
H	-6.77786	1.60215	0.00748
H	-7.88353	-0.61141	0.20547
H	-6.54678	-2.68194	0.29898
H	-4.06360	-2.60759	0.19676

***E*-6**

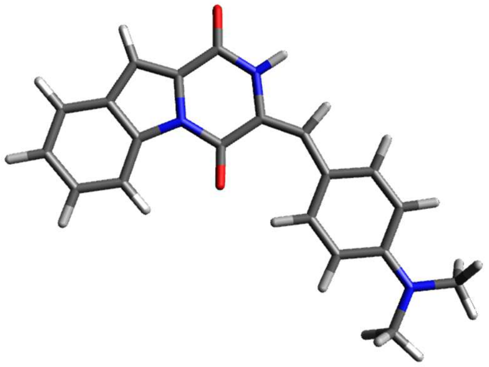

C	2.36662	-0.53955	0.00295
C	2.18945	0.86394	0.00018
C	3.37697	1.63999	-0.00238
C	4.63547	1.08177	-0.00252
C	4.80242	-0.32741	0.00012
C	3.62059	-1.11347	0.00299
C	0.95887	1.61207	0.00013
N	6.03921	-0.89808	-0.00002
C	6.18184	-2.34818	0.00320
C	7.23444	-0.06479	-0.00249
C	-0.39160	1.37512	0.00051
N	-1.21788	2.52861	0.00083
C	-2.57965	2.59623	0.00087
C	-3.23389	1.29416	0.00038
N	-2.46322	0.11733	-0.00010
C	-1.06326	0.06930	-0.00007
O	-0.49485	-1.01314	-0.00069
O	-3.18982	3.66348	0.00128
C	-4.56066	0.97970	0.00023
C	-4.66146	-0.45174	-0.00037
C	-3.34110	-0.97605	-0.00059
C	-5.75515	-1.32867	-0.00075
C	-5.51357	-2.69385	-0.00132
C	-4.19954	-3.19431	-0.00152
C	-3.09496	-2.34983	-0.00116
H	1.49663	-1.17373	0.00507
H	3.29426	2.72164	-0.00436
H	5.49642	1.73411	-0.00461
H	3.69093	-2.19179	0.00529
H	1.16861	2.68009	-0.00038

H	5.72218	-2.79868	-0.88253
H	7.23939	-2.59929	0.00221
H	5.72478	-2.79456	0.89238
H	7.27694	0.57357	-0.89111
H	7.27863	0.57652	0.88394
H	8.11232	-0.70571	-0.00227
H	-0.75278	3.42720	0.00121
H	-5.36848	1.69346	0.00051
H	-6.76663	-0.94062	-0.00058
H	-6.34580	-3.38750	-0.00161
H	-4.04047	-4.26602	-0.00198
H	-2.08743	-2.73292	-0.00134

***Z*-7**

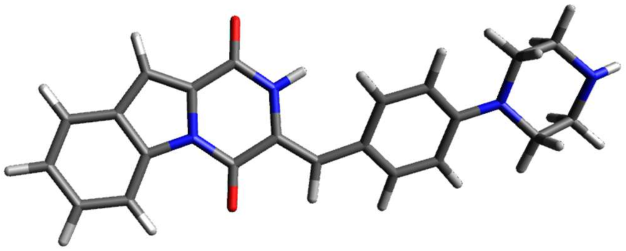

C	2.14637	-1.80072	-0.37303
C	1.21847	-0.82404	0.04558
C	1.75884	0.38813	0.51686
C	3.12211	0.62512	0.52897
C	4.04546	-0.33388	0.05020
C	3.50644	-1.57055	-0.38057
C	-0.18993	-1.14920	0.00575
N	5.40346	-0.07459	-0.00956
C	5.90896	1.21839	0.45824
C	7.28456	1.51012	-0.13150
N	8.20259	0.42004	0.18864
C	7.72170	-0.81691	-0.42288
C	6.36301	-1.17427	0.16461
C	-1.27546	-0.33674	-0.05631
N	-1.19963	1.05764	-0.18288
C	-2.24800	1.93917	-0.23319

C	-3.56218	1.31465	-0.11628
N	-3.68642	-0.08657	-0.03412
C	-2.61229	-0.97485	-0.01341
O	-2.78755	-2.18057	0.05525
O	-2.08294	3.14560	-0.37621
C	-4.80008	1.88613	-0.10618
C	-5.76397	0.82608	-0.01132
C	-5.05039	-0.40036	0.03066
C	-7.16461	0.81281	0.04047
C	-7.81433	-0.40875	0.13201
C	-7.08884	-1.61269	0.17250
C	-5.69933	-1.63129	0.12275
H	1.77689	-2.76011	-0.71799
H	1.11873	1.14176	0.95793
H	3.46978	1.55783	0.94635
H	4.15442	-2.35455	-0.74355
H	-0.43572	-2.20446	-0.01099
H	5.22855	2.00470	0.13610
H	5.96526	1.23856	1.55689
H	7.17306	1.65714	-1.21937
H	7.65925	2.44130	0.29878
H	9.12909	0.63864	-0.16144
H	8.42229	-1.62658	-0.20775
H	7.62102	-0.73252	-1.51842
H	6.47661	-1.39515	1.23604
H	5.99577	-2.07049	-0.32632
H	-0.29177	1.46930	-0.35104
H	-4.99553	2.94471	-0.16395
H	-7.72250	1.74087	0.00896
H	-8.89643	-0.44029	0.17286
H	-7.62432	-2.55180	0.24391

H	-5.13965	-2.55263	0.15273

***E*-7**

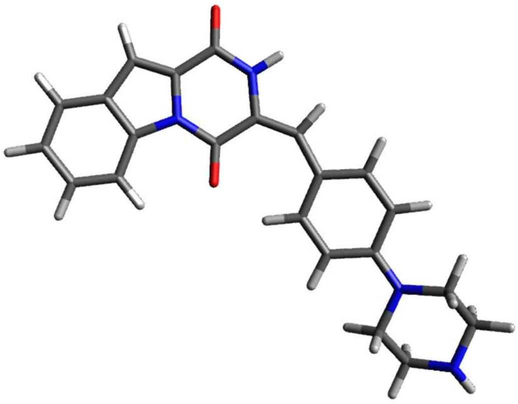

C	1.44899	0.18999	0.06486
C	1.14954	-1.18814	0.01424
C	2.26534	-2.06032	-0.05211
C	3.56592	-1.60958	-0.07383
C	3.86118	-0.22520	-0.01361
C	2.75150	0.65185	0.04918
C	-0.14162	-1.83130	0.03385
N	5.16207	0.23489	0.01436
C	6.22386	-0.51241	-0.67390
C	7.59009	-0.19995	-0.07852
N	7.82237	1.24166	-0.13797
C	6.81955	1.93246	0.66899
C	5.42779	1.67293	0.10113
C	-1.46504	-1.47996	0.04775
N	-2.38853	-2.55445	0.09548
C	-3.75179	-2.50081	0.10334
C	-4.28887	-1.14707	0.05307
N	-3.41762	-0.04325	0.00560
C	-2.02032	-0.11885	-0.00212
O	-1.35683	0.90574	-0.05475
O	-4.45288	-3.50888	0.14970
C	-5.58247	-0.71700	0.04238
C	-5.55714	0.71693	-0.01458
C	-4.19610	1.12263	-0.03744
C	-6.56947	1.68584	-0.04790
C	-6.20862	3.02344	-0.10234
C	-4.85586	3.40583	-0.12413
C	-3.82970	2.46799	-0.09233

H	0.64069	0.90014	0.10166
H	2.09198	-3.13059	-0.08255
H	4.35993	-2.34095	-0.10723
H	2.90466	1.72062	0.06356
H	-0.02200	-2.91297	0.04131
H	6.04066	-1.57947	-0.59610
H	6.22332	-0.24417	-1.74034
H	7.62295	-0.58947	0.95328
H	8.35546	-0.71683	-0.66139
H	8.75001	1.45516	0.21210
H	7.01145	3.00732	0.64747
H	6.83158	1.60435	1.72240
H	5.33733	2.14262	-0.88929
H	4.69931	2.13241	0.76597
H	-2.00485	-3.48998	0.13580
H	-6.45000	-1.35620	0.07196
H	-7.61123	1.38896	-0.03101
H	-6.97657	3.78718	-0.12865
H	-4.60318	4.45852	-0.16701
H	-2.79241	2.76078	-0.10993

***Z*-8**

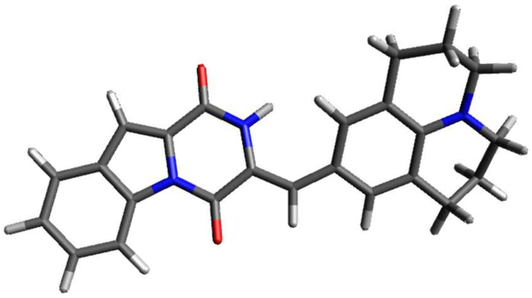

C	5.59621	0.68880	0.13506
C	4.83120	-0.48292	-0.10757
N	3.48291	-0.13117	0.02799
C	3.41746	1.23811	0.35049
C	4.67845	1.75397	0.42285
C	2.36977	-0.96049	-0.13416
C	1.06726	-0.28861	0.03226
N	1.04897	1.06697	0.39982
C	2.13074	1.88393	0.59091
O	2.01703	3.05326	0.94631

O	2.50139	-2.14337	-0.41310
C	-0.05682	-1.03291	-0.15870
C	-1.44788	-0.66284	-0.14186
C	-2.40475	-1.68371	0.04688
C	-3.76239	-1.44186	0.08188
C	-4.24216	-0.11004	-0.06943
C	-3.29963	0.93342	-0.29224
C	-1.94965	0.64273	-0.33546
N	-5.58221	0.15815	-0.02154
C	-6.13042	1.48044	-0.32409
C	-5.17537	2.31716	-1.16235
C	-3.79990	2.34200	-0.49792
C	-4.75781	-2.55660	0.28602
C	-5.96236	-2.03492	1.06895
C	-6.57756	-0.85476	0.33167
C	6.99515	0.62783	0.06309
C	7.59274	-0.58447	-0.24550
C	6.81717	-1.73371	-0.48279
C	5.42875	-1.70469	-0.41862
H	4.91766	2.77822	0.65906
H	0.16032	1.47379	0.65697
H	0.15001	-2.08453	-0.32111
H	-2.05780	-2.70425	0.17514
H	-1.28130	1.45727	-0.58684
H	-6.36874	2.00184	0.61311
H	-7.07350	1.33317	-0.85832
H	-5.58122	3.32516	-1.26875
H	-5.09262	1.88512	-2.16459
H	-3.87899	2.85303	0.46978
H	-3.08256	2.90863	-1.09581
H	-4.28199	-3.39235	0.80328

H	-5.09895	-2.93592	-0.68545
H	-5.64233	-1.71911	2.06684
H	-6.71994	-2.81103	1.19488
H	-7.08427	-1.20020	-0.57976
H	-7.33519	-0.37206	0.95626
H	7.59162	1.51349	0.24607
H	8.67236	-0.65182	-0.30555
H	7.31260	-2.66716	-0.72169
H	4.83038	-2.58346	-0.59937

***E*-8**

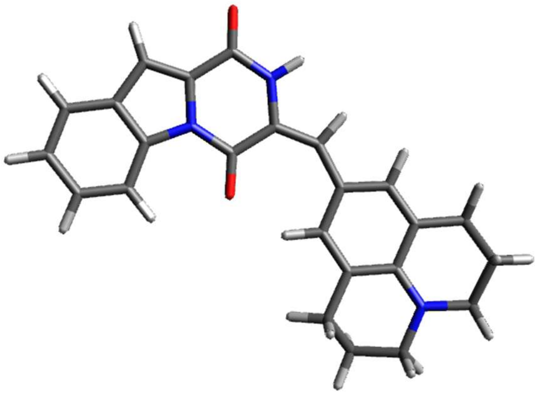

C	5.41582	0.38721	0.03391
C	4.09316	0.90691	0.04596
N	3.21934	-0.18786	0.00288
C	3.99342	-1.36117	-0.03483
C	5.31992	-1.04314	-0.01710
C	1.81673	-0.14391	-0.00144
C	1.15173	-1.44728	-0.05064
N	1.98203	-2.59924	-0.08711
C	3.34287	-2.66410	-0.08365
O	3.95688	-3.72970	-0.11867
O	1.24803	0.93933	0.03496
C	-0.20046	-1.69242	-0.06533
C	-1.43210	-0.95463	-0.04342
C	-2.61509	-1.73823	-0.07270
C	-3.88137	-1.19813	-0.07114
C	-4.03645	0.21630	-0.00617
C	-2.86547	1.02683	0.03715
C	-1.61448	0.44726	0.00341
N	-5.27892	0.78047	0.01420
C	-5.48931	2.22758	-0.05405

C	-4.28537	2.95583	-0.63240
C	-3.02205	2.52620	0.11190
C	-5.11258	-2.06837	-0.12606
C	-6.24687	-1.40874	0.65720
C	-6.50472	-0.01475	0.10462
C	6.50639	1.26783	0.07035
C	6.26021	2.63117	0.11742
C	4.94421	3.12712	0.12880
C	3.84285	2.27956	0.09345
H	6.12994	-1.75409	-0.03805
H	1.51874	-3.49807	-0.12055
H	-0.40255	-2.76138	-0.10374
H	-2.52299	-2.81955	-0.10784
H	-0.74496	1.08318	0.02385
H	-5.71742	2.60923	0.95035
H	-6.37362	2.40395	-0.67321
H	-4.44709	4.03270	-0.55177
H	-4.18637	2.71409	-1.69537
H	-3.09988	2.84224	1.15994
H	-2.13566	3.01308	-0.30002
H	-4.88622	-3.06141	0.26805
H	-5.42896	-2.20415	-1.16790
H	-5.97187	-1.33934	1.71434
H	-7.16647	-1.99383	0.59226
H	-6.96931	-0.08001	-0.88852
H	-7.20240	0.52611	0.75052
H	7.51933	0.88352	0.06153
H	7.09006	3.32716	0.14606
H	4.78147	4.19767	0.16609
H	2.83386	2.65864	0.10236

Z-9

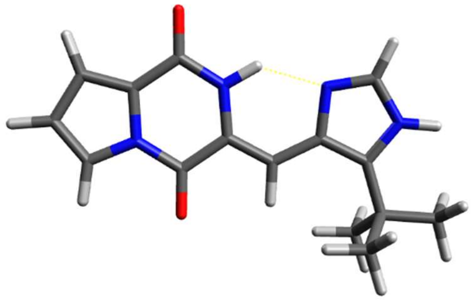

C	3.59449	0.4124	-0.00006
N	3.10583	-0.90204	0.00004
C	4.97455	0.34294	-0.00007
C	5.33458	-1.03858	0.00002
C	4.17364	-1.78493	0.00009
C	2.68254	1.55575	-0.00012
O	3.04678	2.72992	-0.00022
N	1.33576	1.20096	-0.00009
C	1.73738	-1.25518	0.00008
C	0.81473	-0.08836	0.00002
O	1.387	-2.42812	0.00017
C	-0.53067	-0.35687	0.00007
C	-1.59092	0.61167	0.0001
C	-2.98158	0.39461	0.00005
N	-3.5198	1.66672	0.00009
N	-1.32783	1.98334	0.00027
C	-2.49854	2.58148	0.00025
C	-3.83802	-0.86101	-0.00009
C	-3.53847	-1.69879	-1.26859
C	-5.33927	-0.49808	-0.0003
C	-3.53884	-1.69884	1.26846
H	5.63474	1.20351	-0.00014
H	6.34172	-1.4429	0.00004
H	3.98808	-2.852	0.00016
H	0.63742	1.95955	-0.00018
H	-0.7799	-1.41591	0.00009
H	-4.51157	1.87835	0.00003
H	-2.67015	3.65313	0.00034
H	-4.15803	-2.60792	-1.27338
H	-2.48642	-2.00638	-1.31488
H	-3.76153	-1.12534	-2.17944
H	-5.62418	0.07347	-0.89736
H	-5.62441	0.07351	0.89667
H	-5.94002	-1.4178	-0.00036
H	-3.76213	-1.12541	2.17927
H	-2.48681	-2.00645	1.31503
H	-4.15842	-2.60795	1.27305

***E*-9**

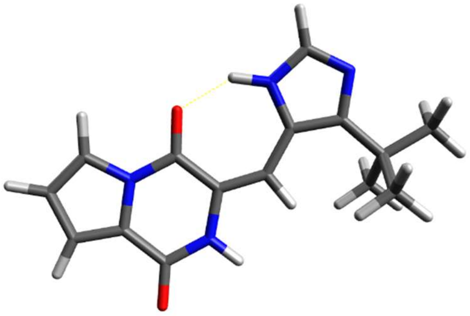

C	-3.6469	0.59146	0.00023
N	-3.02132	-0.66485	-0.00012
C	-5.01177	0.38069	0.00059
C	-5.22546	-1.03031	0.00048
C	-3.99435	-1.65342	0.00004
C	-2.85011	1.80939	-0.00012
O	-3.29985	2.95358	-0.00016
N	-1.47819	1.56362	-0.00055
C	-1.6311	-0.89096	-0.00028
C	-0.80622	0.32358	-0.00027
O	-1.22147	-2.06086	-0.00049
C	0.58085	0.35757	-0.00011
C	1.61078	-0.61954	0.00002
C	3.02846	-0.44986	0.00008
N	3.63232	-1.6723	0.00004
N	1.42606	-1.99605	-0.00001
C	2.64529	-2.57312	0.00003
C	3.86173	0.82652	0.00015
C	5.36048	0.46047	0.00036
C	3.56807	1.66486	-1.26986
C	3.56777	1.66501	1.26997
H	-5.75762	1.16802	0.00088
H	-6.18492	-1.53728	0.00073
H	-3.70175	-2.69575	-0.00013
H	-0.91429	2.41114	-0.00084
H	0.98332	1.37254	-0.00006
H	0.47397	-2.39746	-0.00024
H	2.78454	-3.65004	0.00005
H	5.96175	1.38167	0.00035
H	5.62463	-0.13402	0.88408
H	5.62485	-0.13417	-0.88319
H	4.20668	2.5607	-1.28277
H	2.52316	1.99999	-1.32519
H	3.78014	1.0823	-2.17734
H	3.77954	1.08255	2.17758
H	4.20645	2.5608	1.28294
H	2.52287	2.00025	1.32499

***Z*-10**

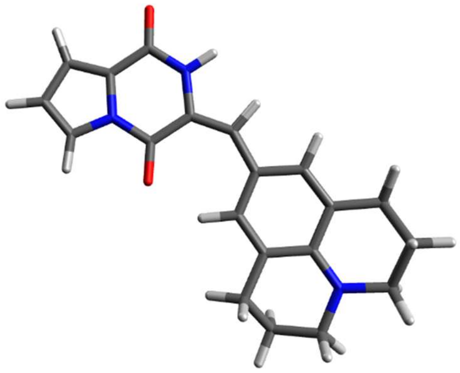

C	4.53702	0.76424	0.26029
N	4.48201	-0.57806	-0.13771
C	3.29231	-1.33011	-0.32453
C	2.04715	-0.56129	-0.08848
N	2.15092	0.76845	0.33923
C	3.31773	1.5037	0.55905
O	3.27734	2.66064	0.97599
O	3.34665	-2.50388	-0.66975
C	5.87067	1.12756	0.32601
C	6.64136	-0.01358	-0.04182
C	5.76927	-1.0488	-0.32101
C	0.85939	-1.22076	-0.28775
C	-0.50447	-0.753	-0.21403
C	-4.04406	2.54121	-1.01249
C	-2.25004	0.98507	-0.24572
C	-3.26233	0.00029	-0.04467
C	-2.87813	-1.37165	0.03651
C	-5.09668	-1.84842	1.06205
N	-4.59083	0.36495	0.05005
C	-5.64602	-0.58851	0.398
C	-1.53545	-1.71064	-0.04365
C	-3.95114	-2.42013	0.21924
C	-2.65348	2.43621	-0.37783
C	-0.91831	0.59735	-0.33748
C	-5.04687	1.73485	-0.19126
H	1.30353	1.24052	0.6463
H	6.22937	2.11007	0.61316
H	7.72368	-0.07141	-0.09698
H	5.92518	-2.07406	-0.63359
H	0.99665	-2.28131	-0.50964
H	-4.38115	3.58606	-1.06765
H	-4.01096	2.15023	-2.04147
H	-5.9119	-2.57654	1.17943
H	-4.72522	-1.60396	2.06947
H	-6.21827	-0.85746	-0.51167
H	-6.3515	-0.08	1.07685
H	-1.25916	-2.76523	0.03304
H	-3.52519	-3.31827	0.68854
H	-4.34528	-2.73454	-0.76385

H	-2.67213	2.91448	0.61789
H	-1.90622	2.98223	-0.97154
H	-0.19134	1.37567	-0.57646
H	-5.24126	2.24043	0.77512
H	-6.01307	1.68009	-0.72036

***E*-10**

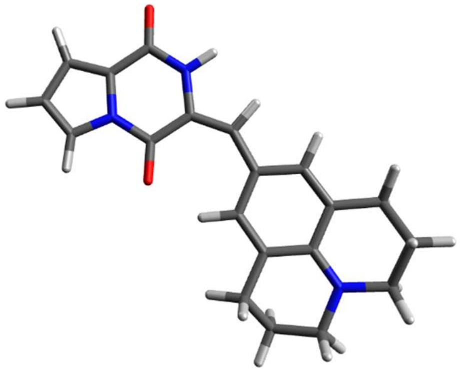

	4.89853	-0.16375	-0.00142
N	3.93572	0.84196	0.07511
C	2.53578	0.65082	0.06248
C	2.11278	-0.74132	-0.04233
N	3.13379	-1.72655	-0.11557
C	4.49022	-1.55207	-0.10482
O	5.27351	-2.49953	-0.1773
O	1.81017	1.63171	0.13991
C	6.13729	0.43282	0.04235
C	5.92655	1.83766	0.14838
C	4.57058	2.06394	0.16673
C	0.82202	-1.21884	-0.07258
C	-0.51404	-0.70137	-0.04215
C	-0.92835	0.65049	0.02211
C	-2.25812	1.01191	0.05304
C	-3.27688	0.01664	-0.00941
C	-2.88718	-1.35184	-0.08989
C	-1.5492	-1.67216	-0.09005
N	-4.59508	0.36433	0.00765
C	-5.67162	-0.62572	0.08034
C	-5.18765	-1.96371	0.61884
C	-3.95534	-2.41457	-0.16423
C	-2.66515	2.46217	0.14609
C	-3.97661	2.68406	-0.60578
C	-5.04576	1.75648	-0.04862
H	2.83678	-2.69046	-0.18889
H	7.08039	-0.088	0.00227
H	6.68911	2.59831	0.20483
H	3.99259	2.96853	0.23554
H	0.80733	-2.30516	-0.14172
H	-0.17735	1.42254	0.05855
H	-1.27653	-2.72187	-0.13952
H	-6.45287	-0.21617	0.72699
H	-6.11289	-0.7545	-0.91707

H	-5.99632	-2.69311	0.54048
H	-4.93353	-1.86268	1.67862
H	-3.56792	-3.36085	0.21919
H	-4.23932	-2.58718	-1.20989
H	-1.86997	3.09717	-0.24978
H	-2.80357	2.74444	1.19748
H	-4.31753	3.71725	-0.51354
H	-3.82991	2.47718	-1.67055
H	-5.34457	2.08109	0.95711
H	-5.94128	1.78785	-0.67583

Molecular Orbitals

The orbital energies and the 3D representations (left: HOMO, right: LUMO) of the electron probability distributions were extracted with Avogadro 1.2.0.

Table S7: HOMO-1 (H-1), HOMO (H), LUMO (L) and LUMO+1 (L+1) Kohn-Sham orbital energies (in eV) of the E-isomers and Z-isomers of the compounds **1-11** calculated on the TD-B3LYP-GD3BJ/6-311G(d,p)level of theory.

Compound		ε_{H-1}	ε_{H}	ε_{L}	ε_{L+1}
**1**	Z	-6,422	-5,176	-2,394	-1,355
	E	-6,379	-5,080	-2,341	-1,321
**2**	Z	-6,591	-6,022	-2,676	-1,515
	E	-6,569	-5,899	-2,635	-1,473
**3**	Z	-6,510	-5,905	-2,425	-1,304
	E	-6,598	-5,877	-2,645	-1,465
**4**	Z	-6,520	-5,698	-2,474	-1,381
	E	-6,524	-5,599	-2,518	-1,402
**5**	Z	-6,626	-6,251	-2,592	-1,484
	E	-6,595	-6,167	-2,627	-1,456
**6**	Z	-6,451	-5,341	-2,406	-1,361
	E	-6,410	-5,287	-2,425	-1,342
**7**	Z	-6,448	-5,446	-2,451	-1,390
	E	-6,418	-5,374	-2,475	-1,367
**8**	Z	-6,408	-5,105	-2,351	-1,328
	E	-6,359	-5,072	-2,358	-1,307
**9**	Z	-6,706	-5,792	-2,445	-0,595
	E	-6,786	-5,799	-2,713	-0,816
**10**	Z	-6,612	-5,134	-2,377	-0,731
	E	-6,550	-5,108	-2,403	-0,653
**11**	Z	-6,484	-5,760	-2,403	-1,285
	E	-6,570	-5,743	-2,631	-1,457

***Z*-1**

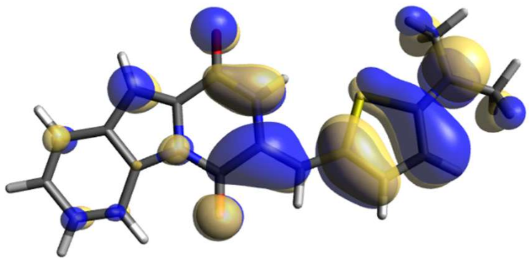

HOMO

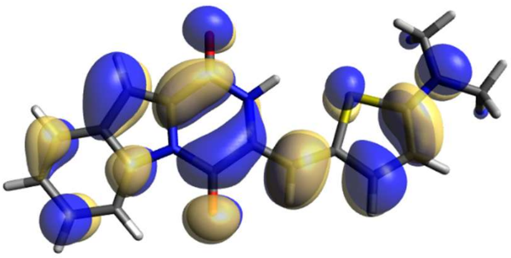

LUMO

***Figure S53**: HOMO and LUMO orbital geometries of compound **Z-1**, calculated on the TD-B3LYP-GD3BJ/6-311G(d,p) level of theory.*

***E*-1**

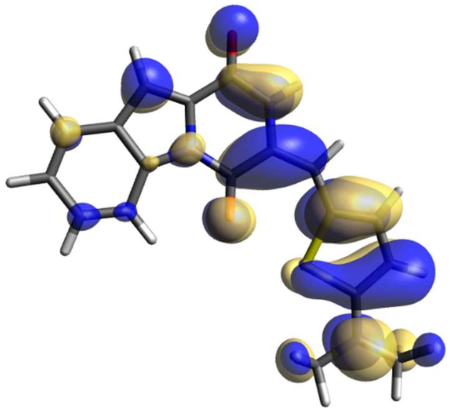

HOMO

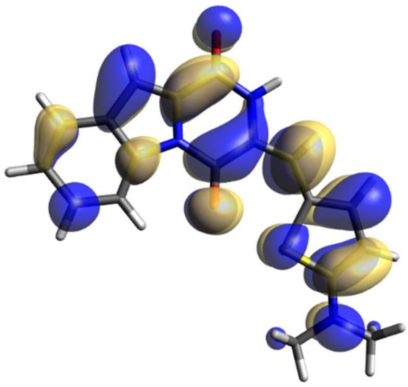

LUMO

***Figure S54**: HOMO and LUMO orbital geometries of compound **E-1**, calculated on the TD-B3LYP-GD3BJ/6-311G(d,p) level of theory.*

***Z*-2**

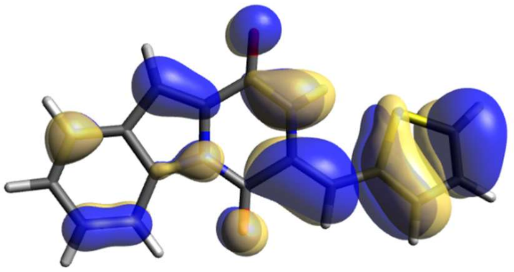

HOMO

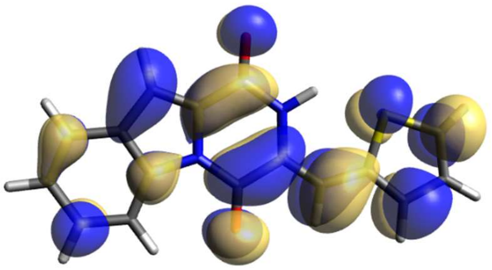

LUMO

***Figure S55**: HOMO and LUMO orbital geometries of compound **Z-2**, calculated on the TD-B3LYP-GD3BJ/6-311G(d,p) level of theory.*

***E*-2**

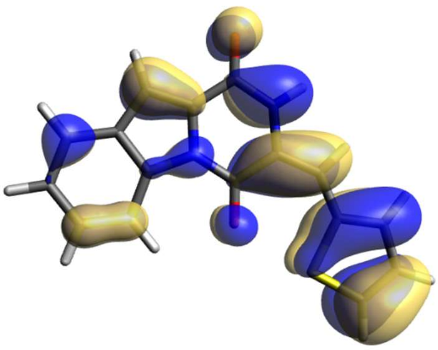

HOMO

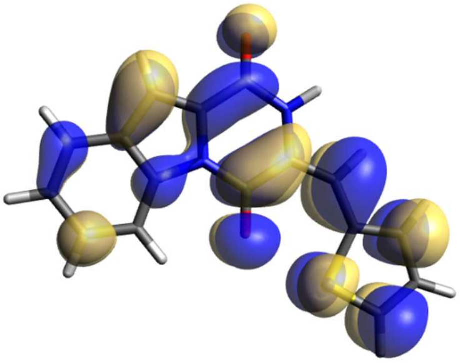

LUMO

***Figure S56**: HOMO and LUMO orbital geometries of compound **E-2**, calculated on the TD-B3LYP-GD3BJ/6-311G(d,p) level of theory.*

Z-3

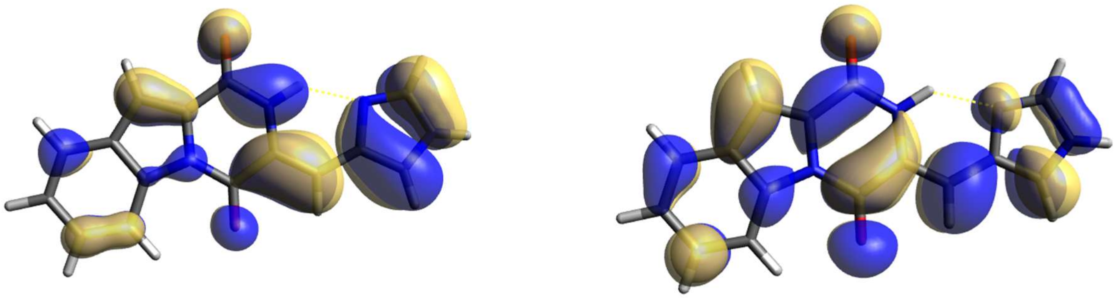

HOMO LUMO

Figure S57: *HOMO and LUMO orbital geometries of compound* ***Z-3****, calculated on the TD-B3LYP-GD3BJ/6-311G(d,p) level of theory.*

***E*-3**

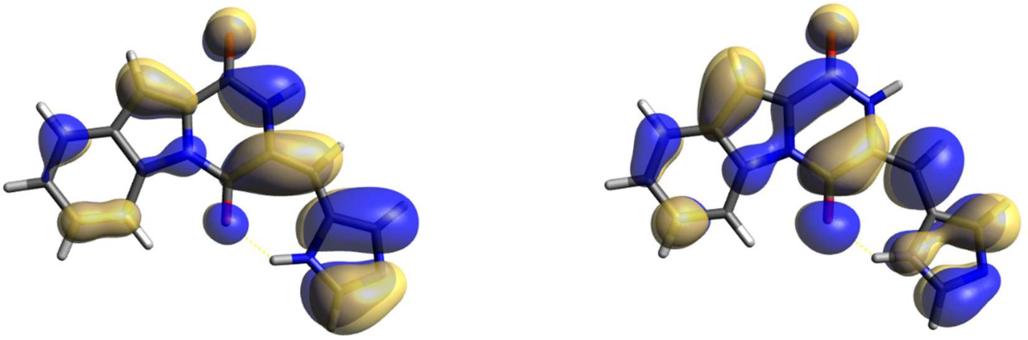

HOMO LUMO

Figure S58: *HOMO and LUMO orbital geometries of compound* ***E-3****, calculated on the TD-B3LYP-GD3BJ/6-311G(d,p) level of theory.*

***Z*-4**

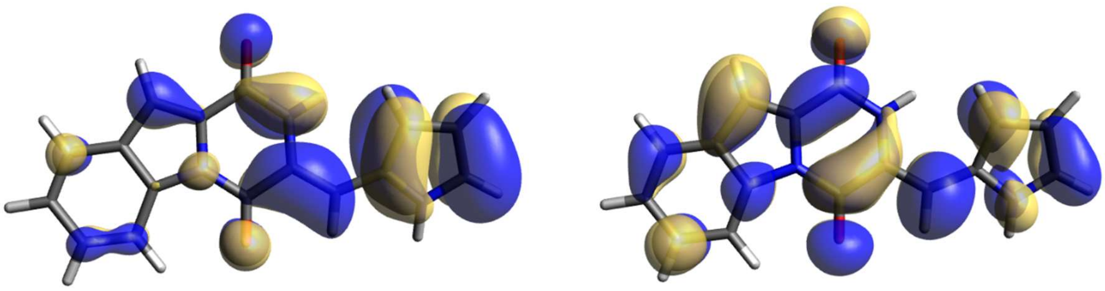

HOMO LUMO

***Figure S59**: HOMO and LUMO orbital geometries of compound **E-4**, calculated on the TD-B3LYP-GD3BJ/6-311G(d,p) level of theory.*

***E*-4**

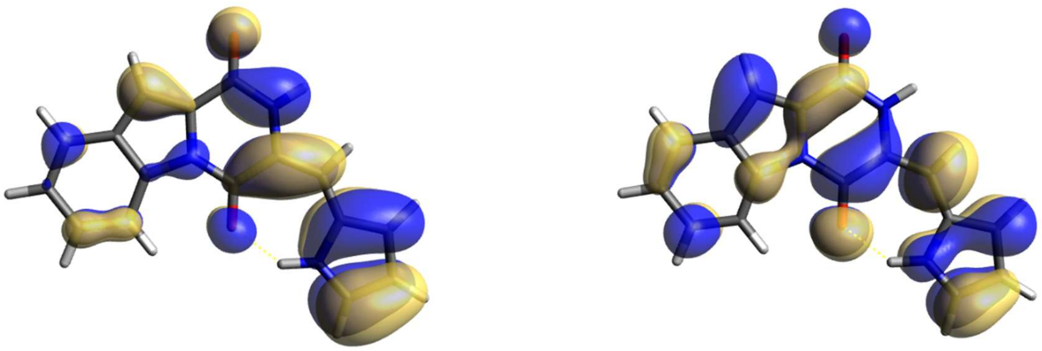

HOMO LUMO

***Figure S60**: HOMO and LUMO orbital geometries of compound **E-4**, calculated on the TD-B3LYP-GD3BJ/6-311G(d,p) level of theory.*

***Z*-5**

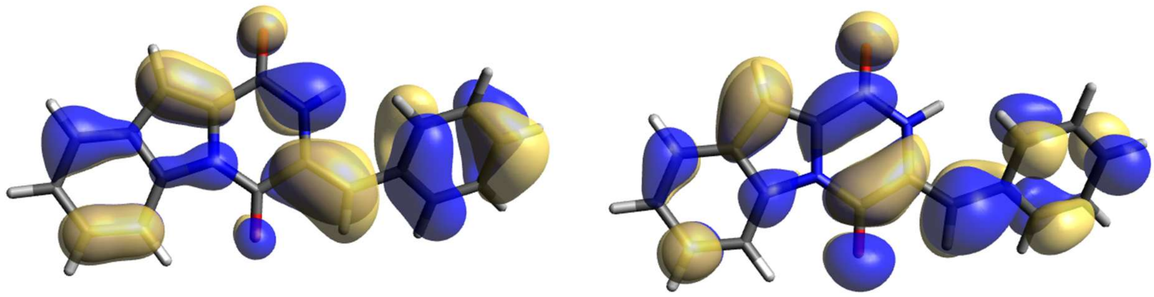

HOMO LUMO

Figure S61*: HOMO and LUMO orbital geometries of compound* ***Z-5****, calculated on the TD-B3LYP-GD3BJ/6-311G(d,p) level of theory.*

***E*-5**

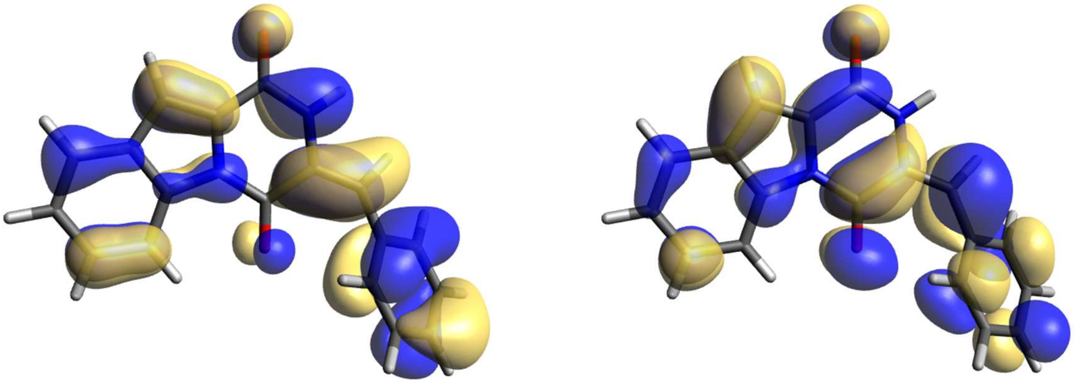

HOMO LUMO

Figure S62*: HOMO and LUMO orbital geometries of compound* ***E-5****, calculated on the TD-B3LYP-GD3BJ/6-311G(d,p) level of theory.*

***Z*-6**

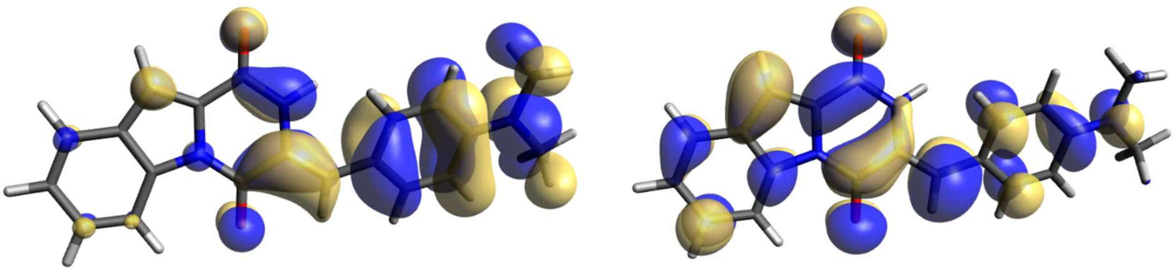

HOMO LUMO

Figure S63*: HOMO and LUMO orbital geometries of compound* ***Z-6****, calculated on the TD-B3LYP-GD3BJ/6-311G(d,p) level of theory.*

***E*-6**

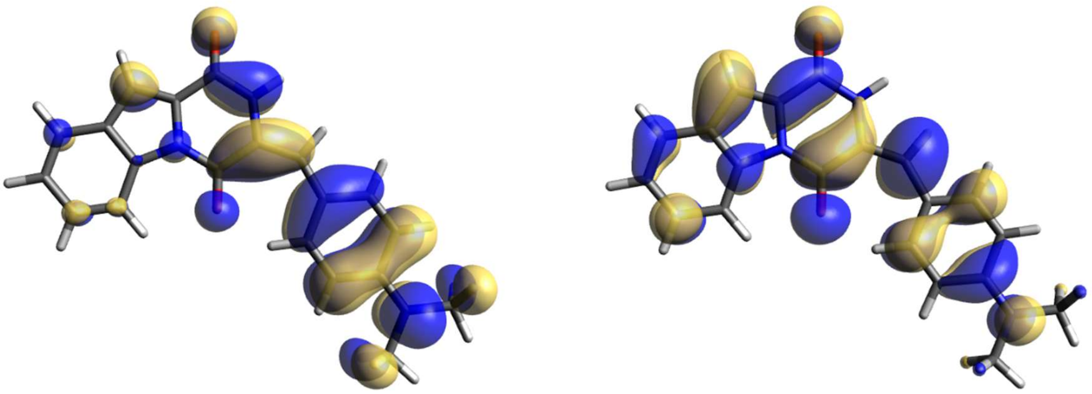

HOMO LUMO

Figure S64*: HOMO and LUMO orbital geometries of compound* ***E-6****, calculated on the TD-B3LYP-GD3BJ/6-311G(d,p) level of theory.*

***Z*-7**

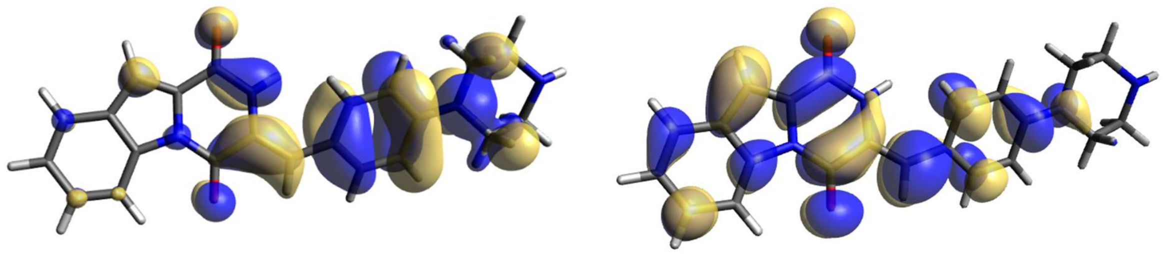

HOMO LUMO

Figure S65: *HOMO and LUMO orbital geometries of compound* ***Z*-7**, *calculated on the TD-B3LYP-GD3BJ/6-311G(d,p) level of theory.*

***E*-7**

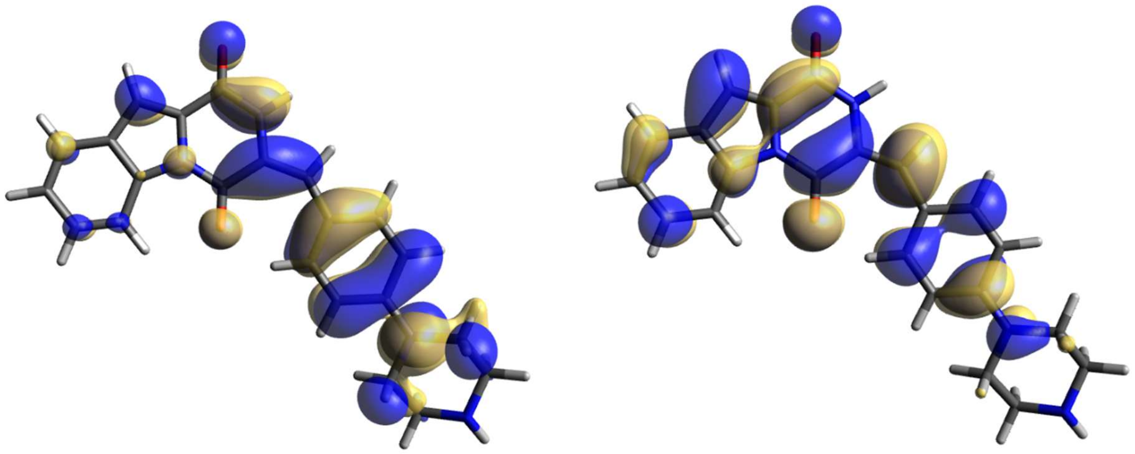

HOMO LUMO

Figure S66: *HOMO and LUMO orbital geometries of compound* ***E*-7**, *calculated on the TD-B3LYP-GD3BJ/6-311G(d,p) level of theory.*

***Z*-8**

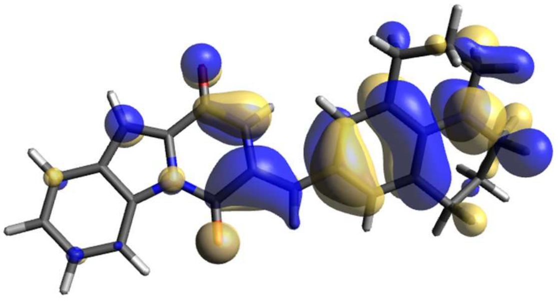

HOMO

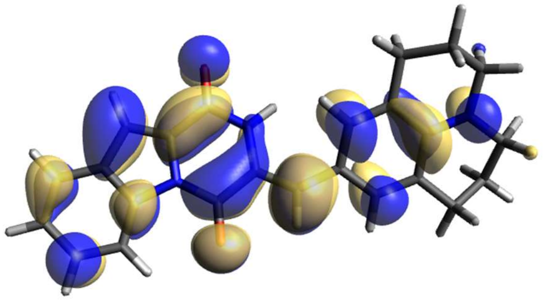

LUMO

Figure S67*: HOMO and LUMO orbital geometries of compound* ***Z-8****, calculated on the TD-B3LYP-GD3BJ/6-311G(d,p) level of theory.*

***E*-8**

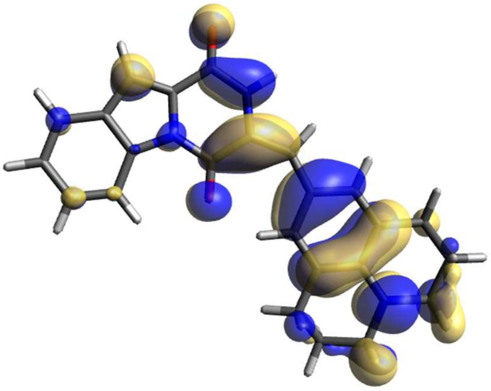

HOMO

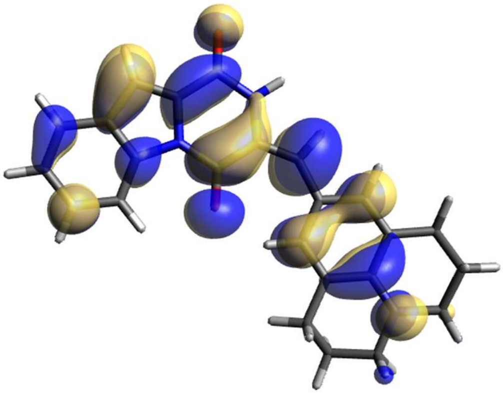

LUMO

Figure S68*: HOMO and LUMO orbital geometries of compound* ***E-8****, calculated on the TD-B3LYP-GD3BJ/6-311G(d,p) level of theory.*

***Z*-9**

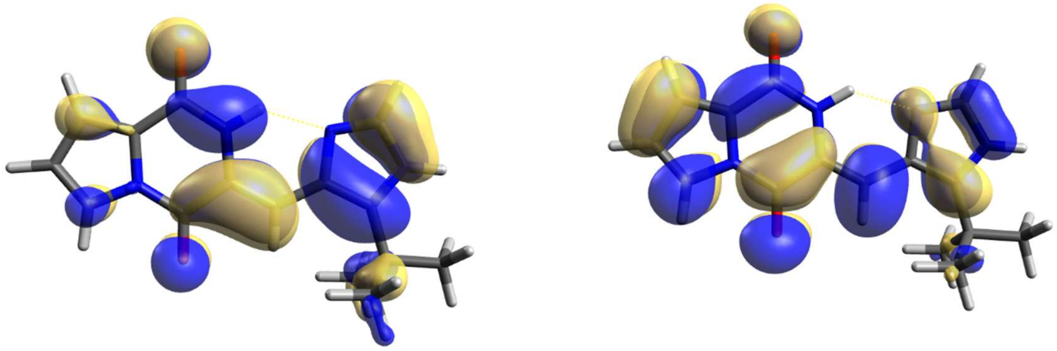

***Figure S69**: HOMO and LUMO orbital geometries of compound **Z-9**, calculated on the TD-B3LYP-GD3BJ/6-311G(d,p) level of theory.*

***E*-9**

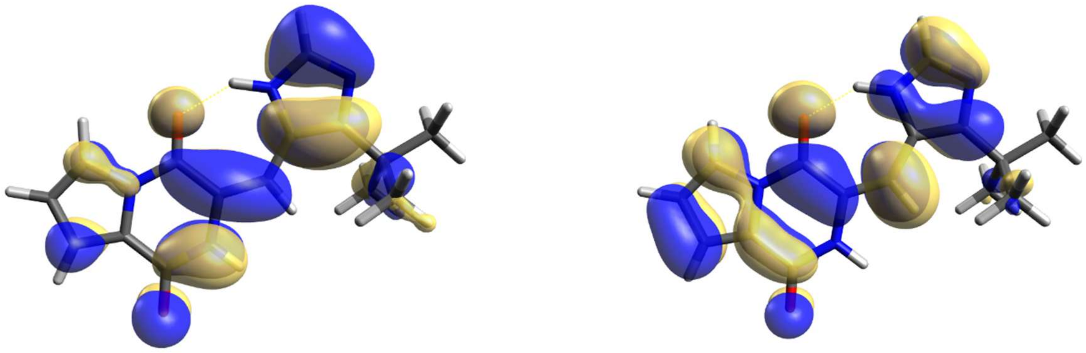

***Figure S70**: HOMO and LUMO orbital geometries of compound **Z-9**, calculated on the TD-B3LYP-GD3BJ/6-311G(d,p) level of theory.*

***Z*-10**

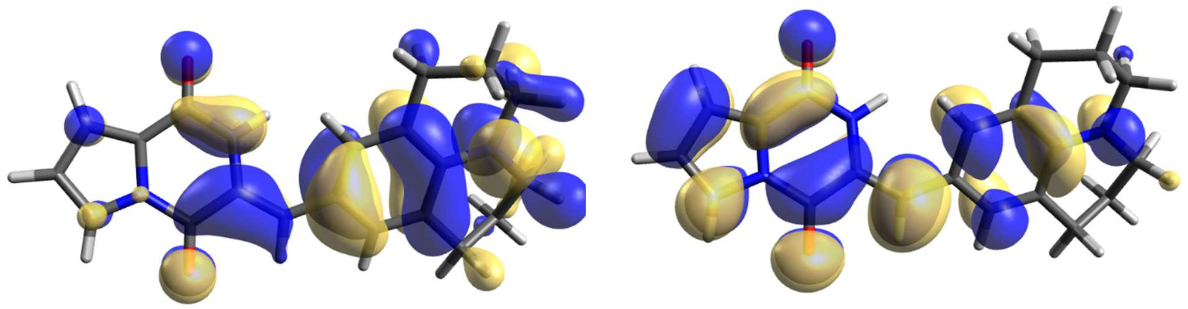

Figure S71: *HOMO and LUMO orbital geometries of compound* ***Z-10****, calculated on the TD-B3LYP-GD3BJ/6-311G(d,p) level of theory.*

***E*-10**

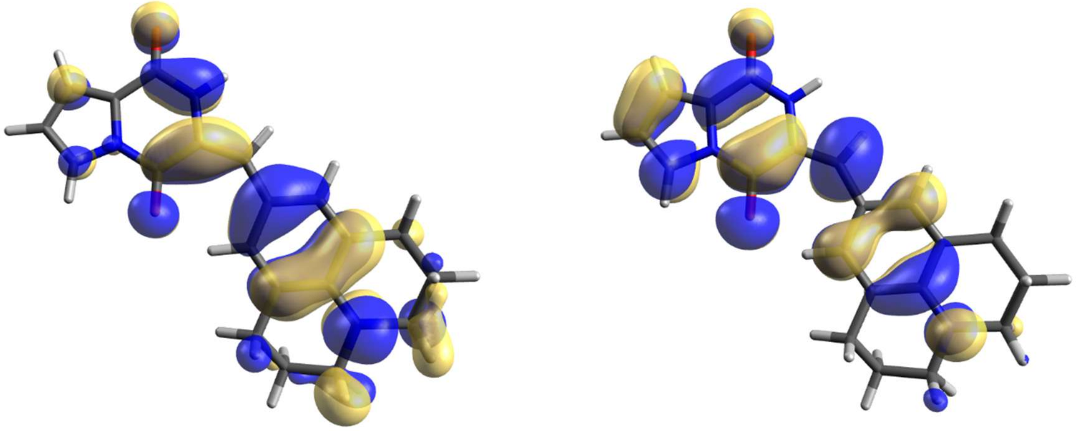

Figure S72: *HOMO and LUMO orbital geometries of compound* ***E-10****, calculated on the TD-B3LYP-GD3BJ/6-311G(d,p) level of theory.*

***Z*-11**

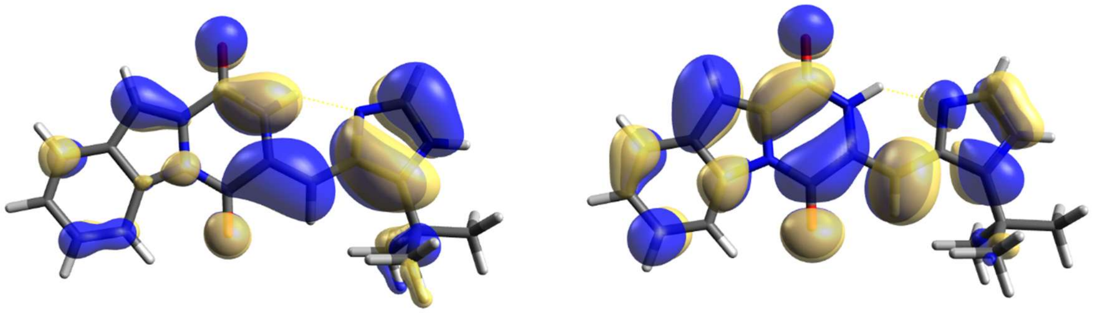

Figure S73: *HOMO and LUMO orbital geometries of compound **Z-11**, calculated on the TD-B3LYP-GD3BJ/6-311G(d,p) level of theory.*

***E*-11**

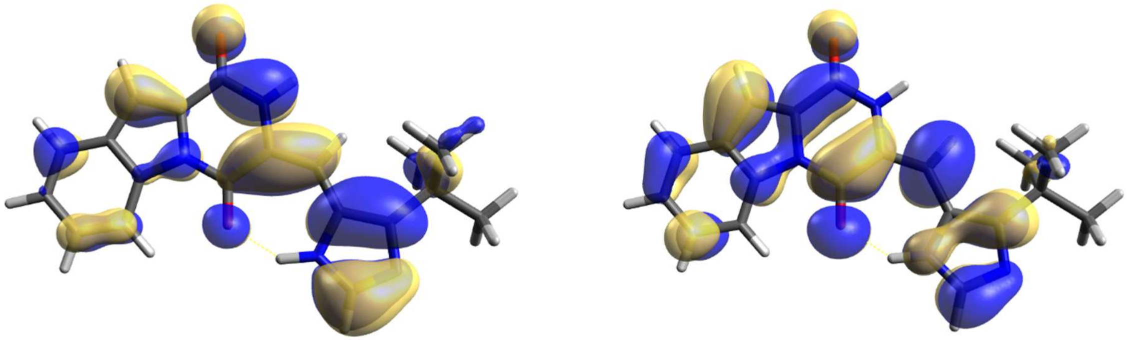

Figure S74: *HOMO and LUMO orbital geometries of compound **E-11**, calculated on the TD-B3LYP-GD3BJ/6-311G(d,p) level of theory.*

Excitation Energies and Calculated Absorption Spectra

Excitation Energies

Excitation energies were then calculated on TD-B3LYP-GD3BJ/6-311G(d,p) level of theory, using previously optimized structures. Solvent interactions were simulated using a polarizable continuum model (PCM), with parameters taken for DMSO. The five lowest transitions computed in the TD calculations (10 states in total) are reported. For each excited state the energy (in eV), the associated wavelength (in nm) and the oscillator strength of the transition is given. The numbers of the orbitals involved in the transition (with general formula occupied → unoccupied) and the associated (largest) coefficient in the CI expansion is presented.

***Z*-1**

Excited State 1: 2.5698 eV 482.48 nm f=1.1295

88 -> 89 0.70427

Excited State 2: 3.3883 eV 365.92 nm f=0.1860

87 -> 89 0.24154

88 -> 90 0.65096

Excited State 3: 3.6328 eV 341.30 nm f=0.0228

86 -> 89 0.12695

87 -> 89 0.64626

88 -> 90 -0.21639

Excited State 4: 3.8742 eV 320.03 nm f=0.0260

86 -> 89 0.66514

88 -> 90 0.12931

88 -> 91 0.11057

Excited State 5: 4.1676 eV 297.49 nm f=0.0307

85 -> 89 0.43615

86 -> 89 -0.10861

88 -> 91 0.35989

88 -> 92 -0.38426

***E*-1**

Excited State 1: 2.5244 eV 491.13 nm f=0.9815

88 -> 89 0.70546

Excited State 2: 3.3206 eV 373.38 nm f=0.0387

87 -> 89 0.18862

88 -> 90 0.67053

Excited State 3; 3.6115 eV 343.31 nm f=0.0840

86 -> 89 0.11152

87 -> 89 0.66143

88 -> 90 -0.16517

Excited State 4: 3.8999 eV 317.92 nm f=0.0132

86 -> 89 0.65529

88 -> 90 0.11834

88 -> 91 -0.14847

Excited State 5: 4.0964 eV 302.67 nm f=0.0957

84 -> 89 -0.20426

85 -> 89 0.48518

86 -> 89 -0.17691

88 -> 91 -0.41710

***Z*-2**

Excited State 1: 3.0429 eV 407.46 nm f=0.9912

75 -> 77 -0.10302

76 -> 77 0.69769

Excited State 2: 3.4944 eV 354.81 nm f=0.0097

75 -> 77 0.68032

76 -> 78 0.12309

Excited State 3: 3.6882 eV 336.16 nm f=0.0422

74 -> 77 0.68616

76 -> 78 -0.12655

Excited State 4: 4.0877 eV 303.31 nm f=0.0271

74 -> 77 0.12072

75 -> 77 -0.11075

76 -> 78 0.66789

Excited State 5: 4.2027 eV 295.01 nm f=0.0364

72 -> 77 -0.20003

73 -> 77 0.66262

***E*-2**

Excited State 1: 2.9682 eV 417.71 nm f=0.8602

75 -> 77 -0.10119

76 -> 77 0.69807

Excited State 2: 3.4923 eV 355.02 nm f=0.0508

75 -> 77 0.67800

76 -> 78 0.12617

Excited State 3: 3.6966 eV 335.40 nm f=0.0525

74 -> 77 0.67344

76 -> 78 -0.17994

Excited State 4: 4.0158 eV 308.74 nm f=0.0043

74 -> 77 0.17344

75 -> 77 -0.12347

76 -> 78 0.65737

Excited State 5: 4.0307 eV 307.60 nm f=0.0248

73 -> 77 0.69708

***Z*-3**

Excited State 1: 3.1712 eV 390.97 nm f=0.8883

72 -> 73 0.69956

Excited State 2: 3.6409 eV 340.53 nm f=0.0283

71 -> 73 0.67636

72 -> 74 0.14441

Excited State 3: 3.8383 eV 323.02 nm f=0.0585

70 -> 73 0.67456

72 -> 74 -0.17274

Excited State 4: 4.1662 eV 297.59 nm f=0.0174

70 -> 73 0.17444

71 -> 73 -0.12660

72 -> 74 0.66043

Excited State 5: 4.2382 eV 292.54 nm f=0.0001

69 -> 73 0.68529

69 -> 74 0.13025

***E*-3**

Excited State 1: 2.9648 eV 418.19 nm f=0.8504

72 -> 73 0.70053

Excited State 2: 3.4912 eV 355.13 nm f=0.0487

71 -> 73 0.67675

72 -> 74 0.11868

Excited State 3: 3.7093 eV 334.25 nm f=0.0518

70 -> 73 0.64892

72 -> 74 -0.25899

Excited State 4: 3.9914 eV 310.63 nm f=0.0067

70 -> 73 0.24459

71 -> 73 -0.12905

72 -> 74 0.63861

Excited State 5: 4.2390 eV 292.48 nm f=0.0001

67 -> 73 0.19287

69 -> 73 0.65257

69 -> 74 0.14345

***Z*-4**

Excited State 1: 2.9402 eV 421.69 nm f=0.9225

72 -> 73 0.70359

Excited State 2: 3.5875 eV 345.60 nm f=0.0907

71 -> 73 0.65970

72 -> 74 -0.22174

Excited State 3: 3.7775 eV 328.22 nm f=0.0417

70 -> 73 0.58189

71 -> 73 0.13161

72 -> 74 0.36215

Excited State 4: 3.9558 eV 313.42 nm f=0.0468

70 -> 73 -0.38227

71 -> 73 0.16445

72 -> 74 0.55425

Excited State 5: 4.2812 eV 289.60 nm f=0.0090

66 -> 73 0.10560

67 -> 73 -0.22462

69 -> 73 0.65384

***E*-4**

Excited State 1: 2.8466 eV 435.55 nm f=0.8969

72 -> 73 0.70459

Excited State 2: 3.5240 eV 351.82 nm f=0.0468

71 -> 73 0.64979

72 -> 74 -0.25014

Excited State 3: 3.6886 eV 336.13 nm f=0.0348

70 -> 73 0.43825

71 -> 73 0.18100

72 -> 74 0.51386

Excited State 4: 3.8920 eV 318.56 nm f=0.0209

70 -> 73 0.54188

71 -> 73 -0.15668

72 -> 74 -0.40506

Excited State 5: 4.0840 eV 303.58 nm f=0.0144

69 -> 73 0.69993

***Z*-5**

Excited State 1: 3.2462 eV 381.93 nm f=0.9162

75 -> 76 0.69761

Excited State 2: 3.6131 eV 343.15 nm f=0.0118

74 -> 76 0.68194

75 -> 77 0.11145

Excited State 3: 3.7701 eV 328.86 nm f=0.0666

73 -> 76 0.69047

Excited State 4: 4.1714 eV 297.23 nm f=0.0055

72 -> 76 0.66290

75 -> 78 -0.15069

Excited State 5: 4.2903 eV 288.99 nm f=0.0029

69 -> 76 0.26844

70 -> 76 0.36605

75 -> 77 0.50690

***E*-5**

Excited State 1: 3.1091 eV 398.78 nm f=0.7200

75 -> 76 0.69825

Excited State 2: 3.5079 eV 353.44 nm f=0.0555

74 -> 76 0.68308

Excited State 3: 3.7152 eV 333.73 nm f=0.0803

73 -> 76 0.68830

75 -> 77 -0.10732

Excited State 4: 3.8884 eV 318.86 nm f=0.0469

69 -> 76 0.14000

71 -> 76 -0.10040

72 -> 76 0.65942

Excited State 5: 4.2124 eV 294.33 nm f=0.0188

69 -> 76 0.24953

70 -> 76 0.40421

71 -> 76 -0.36644

72 -> 76 -0.16426

75 -> 77 -0.26985

***Z*-6**

Excited State 1: 2.6432 eV 469.07 nm f=1.0397

87 -> 88 0.70537

Excited State 2: 3.5208 eV 352.15 nm f=0.2658

86 -> 88 0.42252

87 -> 89 0.55293

Excited State 3: 3.6600 eV 338.75 nm f=0.0053

84 -> 88 0.16831

85 -> 88 -0.10958

86 -> 88 0.54047

87 -> 89 -0.38843

Excited State 4: 3.8827 eV 319.33 nm f=0.0280

84 -> 88 -0.34352

85 -> 88 0.56455

87 -> 89 -0.17885

Excited State 5: 4.0705 eV 304.59 nm f=0.0131

83 -> 88 0.22993

84 -> 88 -0.40666

85 -> 88 -0.22126

87 -> 90 0.46163

***E*-6**

Excited State 1: 2.6118 eV 474.72 nm f=1.1351

87 -> 88 0.70562

Excited State 2: 3.4668 eV 357.64 nm f=0.0747

86 -> 88 0.47191

87 -> 89 0.51485

Excited State 3: 3.5975 eV 344.64 nm f=0.0582

84 -> 88 -0.16816

85 -> 88 -0.14486

86 -> 88 0.49535

87 -> 89 -0.43585

Excited State 4: 3.8568 eV 321.47 nm f=0.0101

84 -> 88 0.26980

85 -> 88 0.60104

86 -> 88 0.10011

87 -> 89 -0.18647

Excited State 5: 4.0672 eV 304.84 nm f=0.0562

84 -> 88 0.61692

85 -> 88 -0.28681

***Z*-7**

Excited State 1: 2.6822 eV 462.24 nm f=1.0718

98 -> 99 0.70534

Excited State 2: 3.5402 eV 350.22 nm f=0.2391

96 -> 99 -0.37794

97 -> 99 0.44474

98 ->100 -0.38236

Excited State 3: 3.6588 eV 338.87 nm f=0.0047

94 -> 99 0.13123

95 -> 99 0.16145

96 -> 99 0.14301

97 -> 99 0.49844

98 ->100 0.42467

Excited State 4: 3.6934 eV 335.69 nm f=0.0069

94 -> 99 -0.11943

95 -> 99 -0.13294

96 -> 99 0.56327

97 -> 99 0.19683

98 ->100 -0.32242

Excited State 5: 3.8673 eV 320.60 nm f=0.0324

94 -> 99 0.22984

95 -> 99 0.59727

98 ->100 -0.23998

***E*-7**

Excited State 1: 2.6304 eV 471.36 nm f=1.1695

98 -> 99 0.70532

Excited State 2: 3.4726 eV 357.03 nm f=0.0870

96 -> 99 -0.20616

97 -> 99 0.57427

98 ->100 -0.34065

Excited State 3: 3.6029 eV 344.13 nm f=0.0310

94 -> 99 0.13337

95 -> 99 -0.19649

96 -> 99 0.22928

97 -> 99 0.37259

98 ->100 0.49359

Excited State 4: 3.6379 eV 340.81 nm f=0.0245

95 -> 99 0.12919

96 -> 99 0.62788

98 ->100 -0.26435

Excited State 5: 3.8335 eV 323.42 nm f=0.0080

94 -> 99 -0.16402

95 -> 99 0.61706

98 ->100 0.24410

***Z*-8**

Excited State 1: 2.5001 eV 495.91 nm f=1.0465

101 ->102 0.70510

Excited State 2: 3.3756 eV 367.30 nm f=0.2533

100 ->102 0.19873

101 ->103 0.6680

Excited State 3: 3.6443 eV 340.21 nm f=0.0806

98 ->102 0.11473

100 ->102 0.65795

101 ->103 -0.17816

Excited State 4: 3.8645 eV 320.83 nm f=0.0091

97 ->102 0.43310

99 ->102 -0.38840

101 ->104 0.36479

Excited State 5: 3.8989 eV 318.00 nm f=0.0120

97 ->102 -0.27284

98 ->102 0.49129

99 ->102 -0.33291

101 ->104 -0.18501

101 ->105 0.10463

***E*-8**

Excited State 1: 2.5014 eV 495.66 nm f=1.1570

101 ->102 0.7059

Excited State 2: 3.3451 eV 370.65 nm f=0.0622

100 ->102 0.22522

101 ->103 0.66070

Excited State 3: 3.5829 eV 346.05 nm f=0.0844

97 ->102 0.13454

100 ->102 0.64799

101 ->103 -0.20239

Excited State 4: 3.7848 eV 327.59 nm f=0.0007

97 ->102 -0.13595

98 ->102 0.60666

99 ->102 -0.24955

101 ->104 0.15622

101 ->105 -0.13879

Excited State 5: 3.8762 eV 319.86 nm f=0.0060

97 ->102 0.50062

99 ->102 -0.44491

101 ->103 0.11032

***Z*-9**

Excited State 1: 3.0764 eV 403.02 nm f=0.7110

75 -> 76 0.70435

Excited State 2: 3.6709 eV 337.75 nm f=0.0218

74 -> 76 0.69698

Excited State 3: 4.1623 eV 297.88 nm f=0.0001

72 -> 76 0.69030

72 -> 77 -0.11855

Excited State 4: 4.3391 eV 285.73 nm f=0.0000

69 -> 76 0.21175

70 -> 76 0.66135

Excited State 5: 4.4464 eV 278.84 nm f=0.0504

71 -> 76 -0.17251

73 -> 76 0.65245

74 -> 77 0.10519

75 -> 77 -0.13196

***E*-9**

Excited State 1: 2.8605 eV 433.44 nm f=0.7183

75 -> 76 0.70618

Excited State 2: 3.4667 eV 357.64 nm f=0.0344

74 -> 76 0.69929

Excited State 3: 4.1254 eV 300.54 nm f=0.0000

72 -> 76 0.69211

72 -> 77 -0.10491

Excited State 4: 4.1634 eV 297.79 nm f=0.0001

68 -> 76 0.19790

70 -> 76 0.66913

Excited State 5: 4.3202 eV 286.99 nm f=0.0148

73 -> 76 0.69010

***Z*-10**

Excited State 1: 2.5591 eV 484.49 nm f=0.9953

88 -> 89 0.70736

Excited State 2: 3.6802 eV 336.89 nm f=0.0366

86 -> 89 -0.16902

87 -> 89 0.67433

Excited State 3: 3.8577 eV 321.40 nm f=0.0008

85 -> 89 0.55185

86 -> 89 0.23240

88 -> 91 0.36604

Excited State 4: 3.9447 eV 314.30 nm f=0.1025

85 -> 89 0.19573

86 -> 89 -0.41822

88 -> 90 0.51693

Excited State 5: 4.0813 eV 303.78 nm f=0.0587

84 -> 89 0.12411

85 -> 89 -0.13576

86 -> 89 0.46562

87 -> 89 0.13878

88 -> 90 0.44436

88 -> 92 -0.11471

***E*-10**

Excited State 1: 2.5412 eV 487.89 nm f=1.0745

88 -> 89 0.70843

Excited State 2: 3.5828 eV 346.06 nm f=0.0267

86 -> 89 -0.14698

87 -> 89 0.68321

Excited State 3: 3.7499 eV 330.63 nm f=0.0008

85 -> 89 0.67363

88 -> 91 -0.18069

Excited State 4: 3.9262 eV 315.79 nm f=0.0341

85 -> 89 -0.10823

86 -> 89 0.57744

87 -> 89 0.10358

88 -> 90 -0.36425

Excited State 5: 4.0849 eV 303.52 nm f=0.0150

83 -> 89 0.15966

86 -> 89 0.33739

88 -> 90 0.56348

88 -> 92 -0.13545

***Z*-11**

Excited State 1: 3.0435 eV 407.37 nm f=0.9145

88 -> 89 0.70304

Excited State 2: 3.6270 eV 341.84 nm f=0.0618

87 -> 89 0.67511

88 -> 90 0.16270

Excited State 3: 3.8308 eV 323.65 nm f=0.0535

86 -> 89 0.64417

88 -> 90 -0.26142

Excited State 4: 4.0623 eV 305.21 nm f=0.0140

86 -> 89 0.26470

87 -> 89 -0.13972

88 -> 90 0.62804

Excited State 5: 4.2294 eV 293.15 nm f=0.0001

84 -> 89 0.68499

84 -> 90 0.12503

***E*-11**

Excited State 1: 2.8439 eV 435.97 nm f=0.9138

88 -> 89 0.70395

Excited State 2: 3.4668 eV 357.64 nm f=0.0389

87 -> 89 0.67713

88 -> 90 0.14030

Excited State 3: 3.6865 eV 336.32 nm f=0.0376

86 -> 89 0.58751

88 -> 90 -0.38062

Excited State 4: 3.8998 eV 317.93 nm f=0.0152

86 -> 89 0.36920

87 -> 89 -0.14784

88 -> 90 0.57089

Excited State 5: 4.2114 eV 294.40 nm f=0.0001

80 -> 89 0.20677

83 -> 89 0.40202

84 -> 89 0.52980

Calculated Absorption Spectra

The absorption spectra were simulated based on TD calculations (10 states in total) with Gaussview assuming a gaussian band shape (characterized by a standard deviation s = 0.2 eV) and the extracted data was plotted using OriginPro 2020 9.7.188 with the peaks, furnished by the calculation. Solvent interactions were simulated using a polarizable continuum model (PCM), with parameters taken for DMSO.

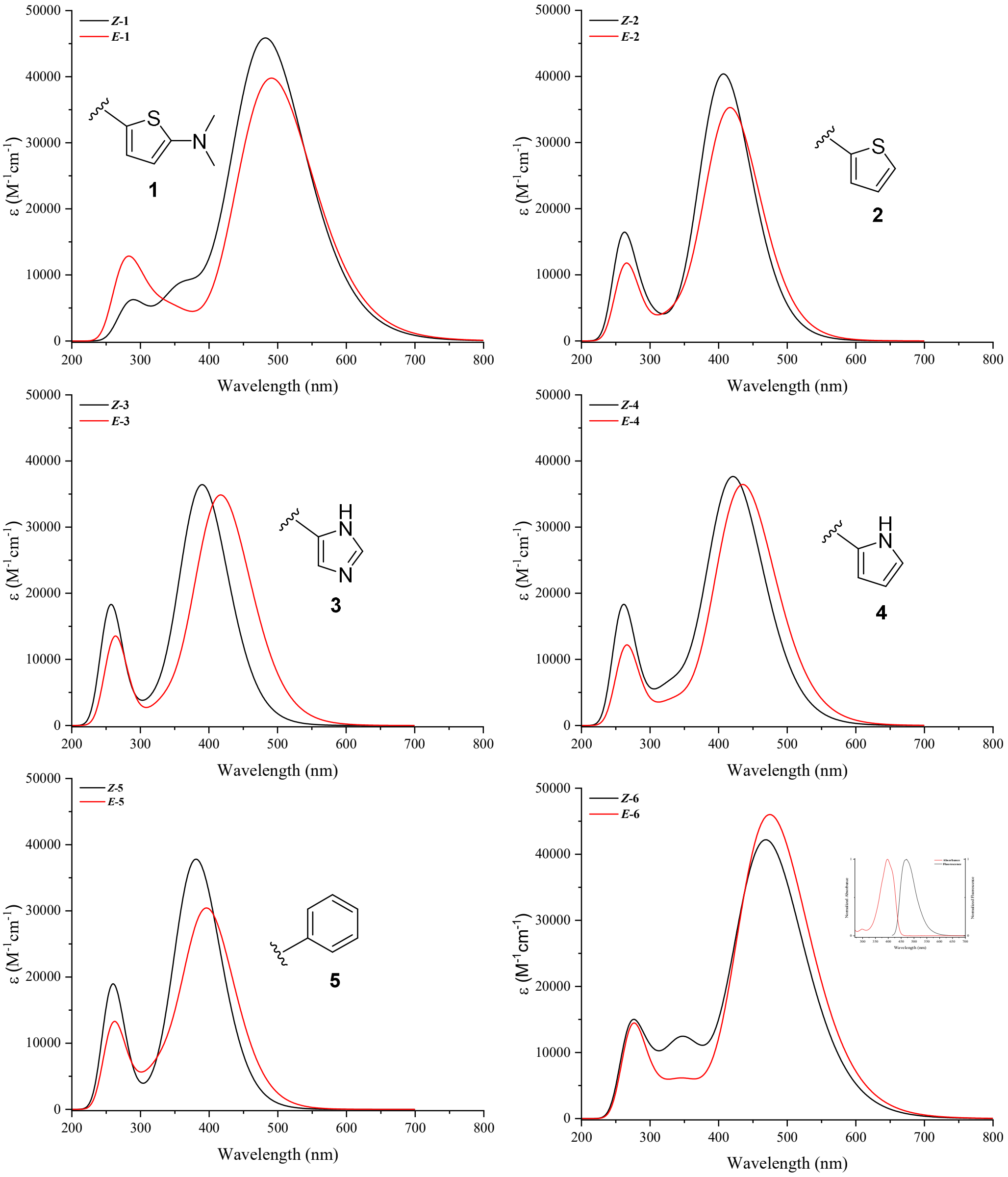

Figure S75: *Simulated absorption spectra of compounds **1-6**, based on TD-DFT calculations on the B3LYP/6-311G(d,p) level.*

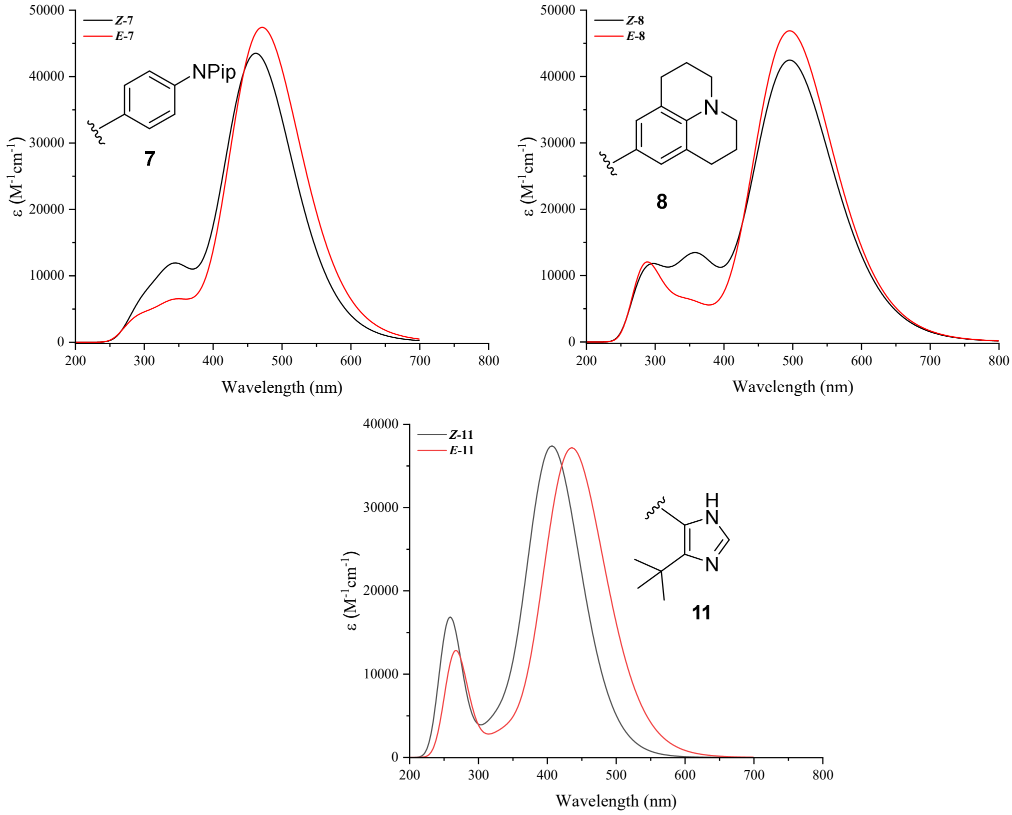

Figure S76: *Simulated absorption spectra of compounds 7,**8** and **11**, based on TD-DFT calculations on the B3LYP/6-311G(d,p) level.*

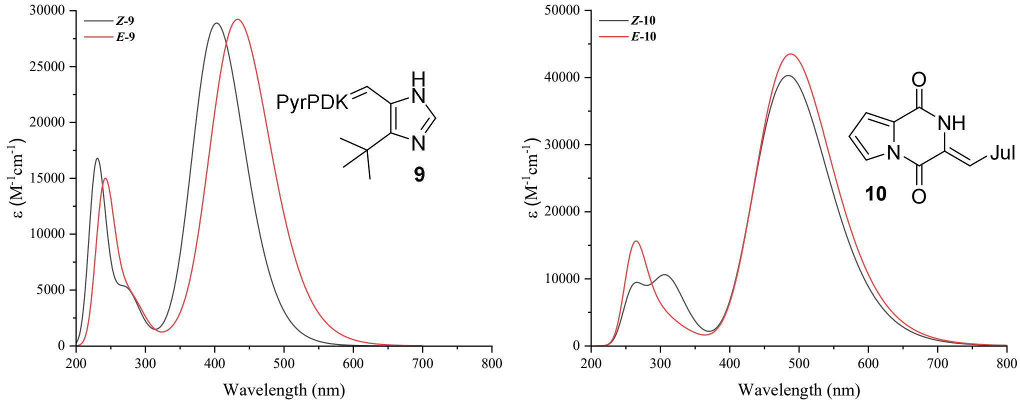

Figure S77: *Simulated absorption spectra of compounds **9** and **10**, based on TD-DFT calculations on the B3LYP/6-311G(d,p) level.*

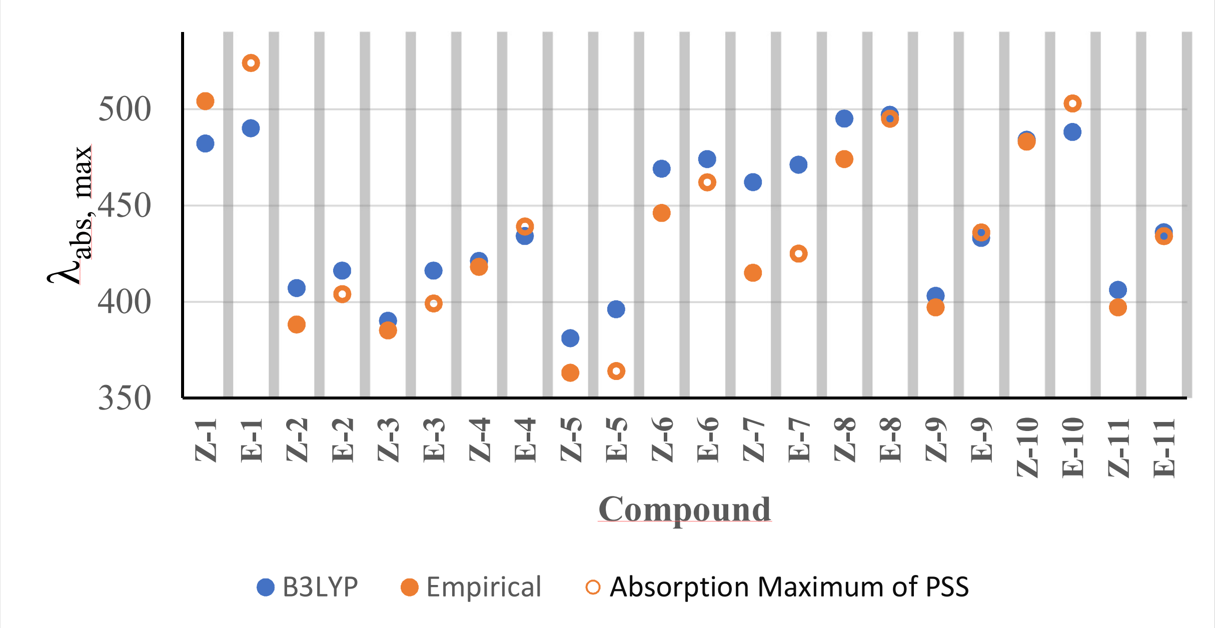

***Figure S78**: Comparison of λ_{max}, as theoretically predicted by TD-B3LYP-GD3BJ/6-311G(d,p) (blue), to experimental data (orange). To compare the calculated values of the respective E-isomers to empirical data, the absorption maxima of the PSS with the highest E-isomer-content were taken as reference values (hollow orange points).*

Natural Bond Orbital (NBO) Analysis

An NBO analysis and a second order perturbation theory deletion analysis of the Fock Matrix was performed on the optimized structures, provided in section “Theoretically Obtained Geometries”. These analyses provided the Wiberg bond indices as well as the total energy change upon exclusion of the indicated intramolecular interactions. The presented data were visualized with GaussView 6.1.1.

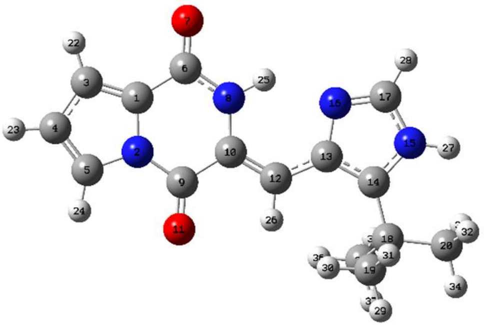

Excerpt from the Wiberg bond index matrix in the NAO basis for ***Z*-9**:

**Atom**		**10**	**11**	**12**	**13**	**14**	**15**	**16**	**17**	**18**
**21.**	C	0.0022	0.0005	0.0014	0.0068	0.0110	0.0068	0.0008	0.0029	0.9822
**22.**	H	0.0000	0.0009	0.0000	0.0000	0.0000	0.0000	0.0000	0.0000	0.0000
**23.**	H	0.0004	0.0002	0.0000	0.0000	0.0000	0.0000	0.0000	0.0000	0.0000
**24.**	H	0.0006	0.0028	0.0000	0.0000	0.0000	0.0000	0.0000	0.0000	0.0000
**25.**	H	0.0035	0.0017	0.0010	0.0002	0.0008	0.0009	**0.0441**	0.0006	0.0000
**26.**	H	0.0068	0.0069	0.8913	0.0043	0.0006	0.0002	0.0077	0.0003	0.0002
**27.**	H	0.0002	0.0000	0.0002	0.0103	0.0042	0.7744	0.0121	0.0022	0.0007
**28.**	H	0.0000	0.0000	0.0002	0.0126	0.0081	0.0009	0.0109	0.9140	0.0001

Second Order Perturbation Theory Analysis of Fock Matrix in NBO Basis:

Donor NBO	Acceptor NBO	kcal/mol
75. LP (1) N 16	/318. BD*(1) N 8 - H 25	13.41

Deletion of the following NBO Fock matrix elements: (75, 318)

Energy change: 0.025857 a.u., 16.226 kcal/mol

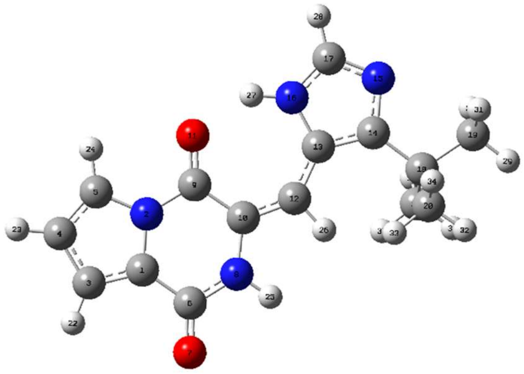

Excerpt from the Wiberg bond index matrix in the NAO basis for ***Z*-9**:

**Atom**		**10**	**11**	**12**	**13**	**14**	**15**	**16**	**17**	**18**
**24.**	H	0.0005	0.0027	0.0000	0.0000	0.0000	0.0000	0.0000	0.0000	0.0000
**25.**	H	0.0034	0.0020	0.0015	0.0002	0.0000	0.0001	0.0000	0.0000	0.0000
**26.**	H	0.0043	0.0002	0.9010	0.0059	0.0016	0.0010	0.0087	0.0002	0.0003
**27.**	H	0.0002	**0.0577**	0.0002	0.0034	0.0079	0.0103	0.6988	0.0038	0.0002
**28.**	H	0.0000	0.0001	0.0001	0.0097	0.0112	0.0106	0.0012	0.9147	0.0001
**29.**	H	0.0000	0.0000	0.0000	0.0001	0.0095	0.0021	0.0004	0.0001	0.0016
**30.**	H	0.0000	0.0000	0.0000	0.0002	0.0006	0.0011	0.0000	0.0001	0.0017
**31.**	H	0.0000	0.0000	0.0000	0.0002	0.0006	0.0011	0.0000	0.0001	0.0017

Second Order Perturbation Theory Analysis of Fock Matrix in NBO Basis:

Donor NBO	Acceptor NBO	kcal/mol
73. LP (2) O 11	/334. BD*(1) N 16 - H 27	11.32

Deletion of the following NBO Fock matrix elements: (73, 334)

Energy change : 0.019086 a.u., 11.977 kcal/mol

Supplementary References

(1) Kelley, E. W.; Norman, S. G.; Scheerer, J. R. Synthesis of monoalkylidene diketopiperazines and application to the synthesis of barettin. *Organic & Biomolecular Chemistry* **2017**, *15* (40), 8634-8640, 10.1039/C7OB02297B. DOI: 10.1039/C7OB02297B.
(2) Kirchner, S.; Leistner, A.-L.; Gödtel, P.; Seliwjorstow, A.; Weber, S.; Karcher, J.; Nieger, M.; Pianowski, Z. Hemipiperazines as peptide-derived molecular photoswitches with low-nanomolar cytotoxicity. *Nature Communications* **2022**, *13* (1), 6066. DOI: 10.1038/s41467-022-33750-7.
(3) Megerle, U.; Lechner, R.; König, B.; Riedle, E. Laboratory apparatus for the accurate, facile and rapid determination of visible light photoreaction quantum yields. *Photochemical & Photobiological Sciences* **2010**, *9* (10), 1400-1406, 10.1039/C0PP00195C. DOI: 10.1039/C0PP00195C.
(4) Brouwer, A. M. Standards for photoluminescence quantum yield measurements in solution (IUPAC Technical Report). *Pure and Applied Chemistry* **2011**, *83* (12), 2213-2228.
(5) Dolomanov, O. V.; Bourhis, L. J.; Gildea, R. J.; Howard, J. A.; Puschmann, H. OLEX2: a complete structure solution, refinement and analysis program. *Journal of applied crystallography* **2009**, *42* (2), 339-341.
(6) Sheldrick, G. M. Crystal structure refinement with SHELXL. *Acta Crystallographica Section C: Structural Chemistry* **2015**, *71* (1), 3-8.
(7) Sheldrick, G. M. SHELXT–Integrated space-group and crystal-structure determination. *Acta Crystallographica Section A: Foundations and Advances* **2015**, *71* (1), 3-8.
(8) Petermayer, C.; Thumser, S.; Kink, F.; Mayer, P.; Dube, H. Hemiindigo: Highly Bistable Photoswitching at the Biooptical Window. *Journal of the American Chemical Society* **2017**, *139* (42), 15060-15067. DOI: 10.1021/jacs.7b07531.
(9) Gödtel, P.; Starrett, J.; Pianowski, Z. L. Heterocyclic Hemipiperazines: Water-Compatible Peptide-Derived Photoswitches. *Chemistry – A European Journal* **2023**, *29* (26), e202204009. DOI: https://doi.org/10.1002/chem.202204009.
